# Identification and characterization of *NF-Y* gene family in walnut (*Juglans regia* L.)

**DOI:** 10.1186/s12870-018-1459-2

**Published:** 2018-10-23

**Authors:** Shaowen Quan, Jianxin Niu, Li Zhou, Hang Xu, Li Ma, Yang Qin

**Affiliations:** 1 Department of Horticulture, College of Agriculture, Shihezi University, Shihezi, Xinjiang, 832003 China; 2 Xinjiang Production and Construction Corps Key Laboratory of Special Fruits and Vegetables Cultivation Physiology and Germplasm Resources Utilization, Shihezi, Xinjiang, 832003 China

**Keywords:** Walnut (*Juglans regia*), NF-Y transcription factor, Phylogenetic analysis, Expression profiles, Transcriptome sequencing

## Abstract

**Background:**

The eukaryotic transcription factor NF-Y (which consists of NF-YA, NF-YB and NF-YC subunits) is involved in many important plant development processes. There are many reports about the NF-Y family in Arabidopsis and other plant species. However, there are no reports about the NF-Y family in walnut (*Juglans regia* L.).

**Results:**

Thirty-three walnut *NF-Y* genes (*JrNF-Ys*) were identified and mapped on the walnut genome. The *JrNF-Y* gene family consisted of 17 *NF-YA* genes, 9 *NF-YB* genes, and 7 *NF-YC* genes. The structural features of the *JrNF-Y* genes were investigated by comparing their evolutionary relationship and motif distributions. The comparisons indicated the *NF-Y* gene structure was both conserved and altered during evolution. Functional prediction and protein interaction analysis were performed by comparing the *Jr*NF-Y protein structure with that in Arabidopsis. Two differentially expressed *JrNF-Y* genes were identified. Their expression was compared with that of three *JrCOs* and two *JrFTs* using quantitative real-time PCR (qPCR). The results revealed that the expression of *JrCO2* was positively correlated with the expression of *JrNF-YA11* and *JrNF-YA12*. In contrast, *JrNF-CO1* and *JrNF-YA12* were negatively correlated.

**Conclusions:**

Thirty-three *JrNF-Ys* were identified and their evolutionary, structure, biological function and expression pattern were analyzed. Two of the *JrNF-Ys* were screened out, their expression was differentially expressed in different development periods of female flower buds, and in different tissues (female flower buds and leaf buds). Based on prediction and experimental data, *JrNF-Ys* may be involved in flowering regulation by co-regulate the expression of flowering genes with other transcription factors (TFs). The results of this study may make contribution to the further investigation of *JrNF-Y* family.

**Electronic supplementary material:**

The online version of this article (10.1186/s12870-018-1459-2) contains supplementary material, which is available to authorized users.

## Background

Nuclear factor Y (NF-Y), which was previously known as heme activator protein (HAP) or CCAAT binding factor (CBF), is a trimeric transcription factor that is present in nearly all eukaryotes. A conserved NF-Y transcription factor has three subunits, NF-YA/B/C (also called HAP2/3/5 or CBF-B/A/C) [1], which can specifically bind to a cis-element, CCAAT-box, in eukaryotic promoters [2]. A single NF-Y subunit cannot regulate transcription. The subunits can only function in the form of a dimer or trimer [3–7]. Initially, NF-YB and NF-YC form a dimer in the cytoplasm. They then bind with NF-YA protein to form a trimer in the nucleus [8, 9]. A recent study suggests that some transcription factors can combine with the NF-YB-YC dimer to form a NF-YB-YC-TF trimer instead of the traditional NF-YB-YC-YA trimer. Both trimers can bind to the promoter region of the target gene and regulate its expression [3].

In many mammals and yeast, a single *NF-Y* gene encodes each NF-Y subfamily [10]. For example, each NF-Y subfamily in mice and humans is encoded by only one *NF-Y* gene. In plants, however, many *NF-Y* genes encode each NF-Y subfamily. For example, it has been reported that in *Arabidopsis thaliana*, the NF-YA subfamily is encoded by ten *NF-YA* genes, the NF-YB subfamily is encoded by thirteen *NF-YB* genes, and the NF-YC subfamily is encoded by thirteen *NF-YC* genes [11]. Other studies indicated that each NF-Y subfamily in *Arabidopsis thaliana* is encoded by ten genes [3, 12].

In recent years, more and more plant *NF-Y* genes have been isolated and identified, including *Triticum aestivum* L. [13], *Arabidopsis thaliana* (L.) Heynh. [11], *populus euphratica* Olivier. [14], *Glycine max* (L.) Merr. [15], *Brassica napus* L. [16], *Phaseolus vulgaris* L. [17], *Physcomitrella patens* (Hedw.) Bruch & Schimp. [18], *Vitis vinifera* L. [19], *Solanum lycopersicum* L. [20], *Citrullus lanatus* (Thunb.) Matsum. & Nakai. [21], *Citrus sinensis* (L.) Osbeck and *citrus clementina* Hort ex Tan [22]. These *NF-Y* genes are involved in many plant developmental processes, such as flowering time regulation [3, 11, 23–33], root growth [34, 35], embryo development [36–42], seed germination [43], meristem formation [44] and fruit maturation [20]. The *NF-Y* genes also participate in plant physiological processes including photosynthesis [45–48] and stress response of endoplasmic reticulum (ER) [49, 50]. In addition, *NF-Y* genes are also involved in plant responses to abiotic stresses [14, 15, 51–58] and in processes related to plant-microbe interactions [59].

The wood and fruit of walnut (*J. regia* L.) are highly valuable, and the research of walnut focus on molecular breeding and flowering in recent years [60–65]. However, less attention were paid to walnut compare with other plants, because it must grow for many years before it becomes productive. The walnut genome was only published recently [66]. The purpose of this study was to identify *NF-Y* gene family in walnut *(JrNF-Y*) and to characterize their structure and function. Flower transition is an important time in plant growth [30], therefore, we focused on this period. Reverse genetic analysis makes it easier to predict the function of the same structural proteins among different species by constructing phylogenetic trees [67] and by analyzing gene expression patterns [28, 68]. The NF-Y family in Arabidopsis has been well characterized and annotated [11]. Therefore, sequencing results from walnut flower buds and leaf buds were searched with Arabidopsis NF-Y protein sequences to identify candidate NF-Y transcription factors in walnut. These candidate NF-Y members were then aligned with the published walnut genome. The NF-Y proteins sequences of walnut and Arabidopsis were aligned and a phylogenetic tree was constructed. The conserved domains of the walnut NF-Y protein sequences were aligned with mouse NF-Y protein sequences to further analyze the evolutionary relationships. The motifs of the walnut NF-Y proteins were predicted to analyze their structural features. The functions of walnut NF-Y members were annotated and their interactions were analyzed based on corresponding NF-Ys in Arabidopsis. Microarray data from transcriptome sequencing was used to construct the expression patterns of *JrNF-Ys* at different stages and in different tissues. Differentially expressed *NF-Y* members and the annotated *FLOWER LOCUS T* (*FT*) and *CONSTANS* (*CO*) genes were identified in the walnut transcriptome, and their relative expression levels were measured using real-time quantitative PCR (qRT-PCR) method. The relative expression levels were used to investigate possible associations among *NF-Y*, *CO*, and *FT*. Published data about walnut protein and cDNA data is limited. Therefore, some walnut *NF-Ys* were probably not included in our retrieval results. However, the results of this experiment provide a beginning point for further study about the *NF-Y* gene family in walnut.

## Results

### Identification and genomic localization of *NF-Ys* in walnut

The full-length protein sequences of Arabidopsis NF-Ys [11] were used to search the walnut transcriptome database using BLAST (version 2.60) [69] and HMMER (version 3.0) software [70]. Eighty-eight candidate *NF-Y* genes were identified in walnut by BLAST. Forty-four candidate genes were identified by HMMER. The results of the two search methods were merged resulting in 104 candidate *NF-Y* genes. Some of the candidate genes were discarded because they were too long or too short or because they had improper domains. Some sequences were considered to be the same gene because their similarity was > 98%. Finally, 33 candidate *NF-Y* genes were identified and translated into amino acid sequences according to the code frame shown in CD-Search (https://www.ncbi.nlm.nih.gov/Structure/cdd/wrpsb.cgi). The 33 candidate genes included 17 *NF-YA* genes, 9 *NF-YB* genes, and 7 *NF-YC* genes. These genes were named *JrNY-F* for *J. regia*. The number after gene name indicated the numerical order of the local gene ID. Each *Jr*NF-Y protein was matched against one NF-Y protein in Arabidopsis (Table 1; priority: Query Cover>Ident>E value).

**Table 1:** NF-Y genes in walnut

Family	Name	Gene ID	Best match in *Juglans regia* Genome				
			Query Cover	E value	Ident	Accession	Alignments Ranges
NF-YA Subfamily	JrNF-YA1	Cluster-14,922.20995	97%	0.0	98%	NW_017389264.1	43,746–44,361,44,488-44,976,45,840-46,105,46,556–46,737
	JrNF-YA2	Cluster-14,922.27809	100%	0.0	99%	NW_017388893.1	293,307–293,587,293,372-294,101,294,234–294,305, 294,531–294,693,295,892-296,322,296,422–296,951
	JrNF-YA3	Cluster-14,922.32667	100%	0.0	84%	NW_017441761.1	116,121–117,095,117,697-117,838,118,791–118,861, 118,959–119,069,119,978-120,307,120,912–121,157
	JrNF-YA4	Cluster-14,922.32809	100%	0.0	99%	NW_017388893.1	291,714–291,800,292,443-292,699,293,985-294,101,294,234- 294,305,294,531-294,693,295,892-296,322,296,422–296,951
	JrNF-YA5	Cluster-14,922.40699	89%	0.0	98%	NW_017442734.1	7508–8233,8334-8501,8723-8792,8858–8973, 9835–10,224,12,530–12,634
	JrNF-YA6	Cluster-14,922.46822	98%	0.0	99%	NW_017436745.1	2743–3638,4155-4319,6065-6137,6280-6398,6591–6912, 7558–7868
	JrNF-YA7	Cluster-14,922.46846	89%	0.0	98%	NW_017442734.1	7508–8233,8334-8501,8723-8973,9835–10,224 12,530–12,634
	JrNF-YA8	Cluster-14,922.46847	89%	0.0	98%	NW_017442734.1	7508–8233,8334-8501,8723-8792,8858–8973 9835–10,233,12,279–12,443
	JrNF-YA9	Cluster-14,922.50230	99%	0.0	99%	NW_017388973.1	706,166–707,095,707,522-707,639,707,717–707,791, 708,295–708,465,708,579–709,333
	JrNF-YA10	Cluster-14,922.52088	83%	0.0	99%	NW_017443622.1	162,218–163,195,163,717-163,881,165,641–165,713, 165,852–165,981,166,205–166,527
	JrNF-YA11	Cluster-14,922.60069	96%	0.0	99%	NW_017388854.1	1,241,389–1,241,687,1,244,904-1,245,304,1,246,166–1,246,427 1,246,636–1,246,803,1,247,015–1,247,884
	JrNF-YA12	Cluster-14,922.69778	99%	0.0	99%	NW_017438713.1	17,627–17,902,19,198-19,481,20,521-21,677,23,066–23,146 22,468–23,146
	JrNF-YA13	Cluster-14,922.72325	90%	0.0	99%	NW_017436745.1	2743–3638,4155–4703
	JrNF-YA14	Cluster-14,922.79874	100%	0.0	99%	NW_017441761.1	115,996–117,095,117,697-117,838,118,791–118,861, 118,959–119,069,119,978-120,307,120,912–121,157
	JrNF-YA15	Cluster-14,922.82902	100%	0.0	99%	NW_017388893.1	291,714–291,800,292,443-292,720,293,981–294,101, 294,234–294,305,294,531-294,693,295,892–296,951
	JrNF-YA16	Cluster-14,922.84458	100%	0.0	99%	NW_017441761.1	115,996–117,095,117,697-117,838,118,791–118,861, 118,959–119,069,119,978–120,934
	JrNF-YA17	Cluster-14,922.95251	100%	0.0	99%	NW_017389365.1	22–832,959-1206,1227-1715,1842–2448
NF-YB Subfamily	JrNF-YB1	Cluster-14,922.21265	100%	0.0	99%	NW_017443611.1	188,307–189,170,197,160-197,297,197,295–197,376
	JrNF-YB2	Cluster-14,922.29372	99%	8e-144	100%	NW_017440443.1	158,098–158,332,158,425–158,702
	JrNF-YB3	Cluster-14,922.32115	99%	0.0	100%	NW_017440443.1	157,907–158,332,158,425-158,554,159,022–159,104, 159,272–159,324,161,023–161,350
	JrNF-YB4	Cluster-14,922.39672	100%	0.0	99%	NW_017388887.1	1,546,260–1,547,648,1,547,636–1,547,998
	JrNF-YB5	Cluster-14,922.54864	99%	6e-165	99%	NW_017440443.1	157,770–157,906,158,098-158,332,158,425–158,554, 159,022–159,104,159,272–159,324,161,023–161,350
	JrNF-YB6	Cluster-14,922.57314	100%	0.0	99%	NW_017441173.1	105,472–107,835
	JrNF-YB7	Cluster-14,922.58354	99%	0.0	99%	NW_017389061.1	97,582–98,028,99,008-99,091,99,750-99,791,99,916–100,435 101,311–101,443,101,707-101,956,102,119–102,407
	JrNF-YB8	Cluster-14,922.64236	98%	0.0	99%	NW_017436168.1	34,788–35,884
	JrNF-YB9	Cluster-14,922.72337	96%	0.0	99%	NW_017443591.1	1,605,638–1,606,317
NF-YC Subfamily	JrNF-YC1	Cluster-14,922.36868	100%	0.0	99%	NW_017443565.1	579,861–578,990,574,608–574,273
	JrNF-YC2	Cluster-14,922.44401	99%	0.0	100%	NW_017389361.1	1,038,089–1,039,233,1,035,966-1,036,022,1,036,219–1,036,261
	JrNF-YC3	Cluster-14,922.50411	74%	0.0	97%	NW_017389324.1	983,265–984,133
	JrNF-YC4	Cluster-14,922.50413	100%	0.0	100%	NW_017389324.1	983,265–984,857
	JrNF-YC5	Cluster-14,922.55754	76%	0.0	99%	NW_017389752.1	432,148–433,061,431,991–432,052
	JrNF-YC6	Cluster-14,922.62047	99%	0.0	99%	NW_017389324.1	983,265–984,362,985,618–985,795
	JrNF-YC7	Cluster-14,922.71538	71%	0.0	97%	NW_017389361.1	1,038,255–1,038,849

**Fig. 1:**
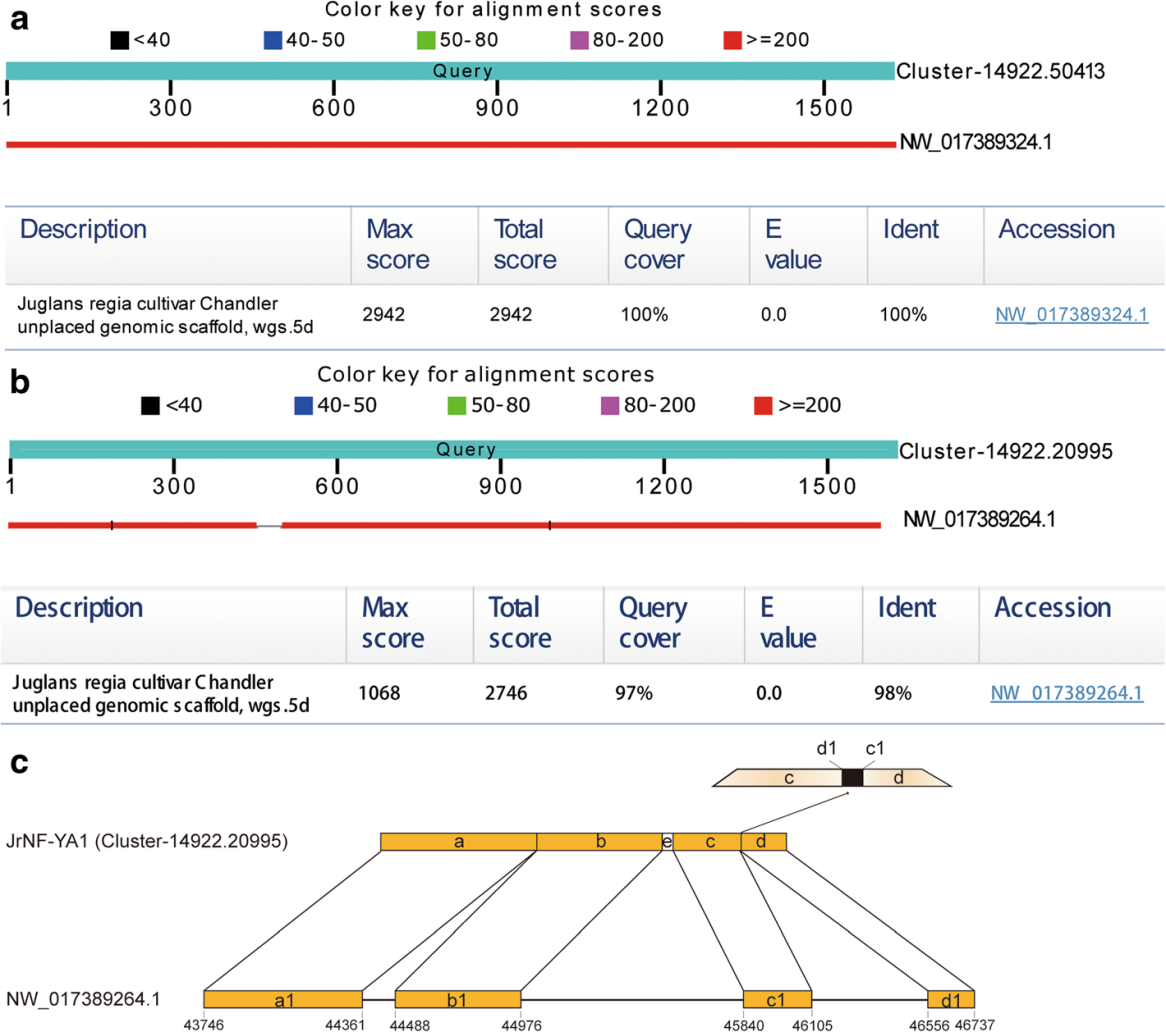
Blast results of the cDNA sequences with the genomic scaffold in walnut. **a** cDNA (Cluster-14,922.50413) was 100% homologous with the genomic scaffold (NW_017389324.1). **b** cDNA (Cluster-14,922.20995) was 98% homologous with the genomic scaffold (NW_017389264.1). **c** Correspondence of different zones between cDNA and the genomic scaffold in walnut; a/b/c/d/e, different segments in cDNA (Cluster-14,922.20995); a1/b1/c1/d1, different segments in the genomic scaffold (NW_017389264.1). The a, b, c, and d segments were respectively matched with a1, b1, c1, and d1. The enlarged part in the upper right shows that (i) the last position of c1 matched the first position of d, (ii) the first position of d1 matched the last position of c, and (iii) c was adjacent to d. The numbers below the scaffold indicate the site number

Walnut genome data was uploaded to NCBI (https://www.ncbi.nlm.nih.gov/bioproject/291087) in 2015. This was an enormous contribution even though the data was spliced at the level of scaffold. We attempted to map the cDNA sequences of the candidate *NF-Ys* on the published walnut genome (GCA 001411555.1wgs.5d scaffolds). In general, cDNA acquired by transcriptome sequencing does not match well against a single scaffold due to post-transcriptional processing. Most of the other *NF-Ys* partially matched the published data [e.g., Cluster-14,922.20995 (*JrNF-YA1*) partially matched NW_017389264.1] (Fig. 1). However, Cluster-14,922.50413 (*JrNF-YC4*) completely matched NW_017389324.1. Optimal matching results of walnut genomic scaffolds for each *JrNF-Y* are shown in Table 1. The table also shows the initiation and termination sites as references for further study.

### Multiple alignments and phylogenetic analyses of the JrNF-Ys

Protein sequences of the three NF-Y subfamilies (i.e., 33 candidate *NF-Y* genes) were aligned using clustalX software [71]. The results showed that each member of the *Jr*NF-Y family member contains an interaction domain for interacting with other NF-Y subunits and a DNA binding domain for recognizing CCAAT binding sites. The three NF-Y subunits of grape, orange and mouse were included as an out-group to root the phylogenetic trees and for comparison. The interaction domain and the DNA binding domain were well conserved between plants and animal. The core conserved regions of the *Jr*NF-YA, *Jr*NF-YB and *Jr*NF-YC proteins were 55, 92, and 107 amino acids long, respectively.

**Fig. 2:**
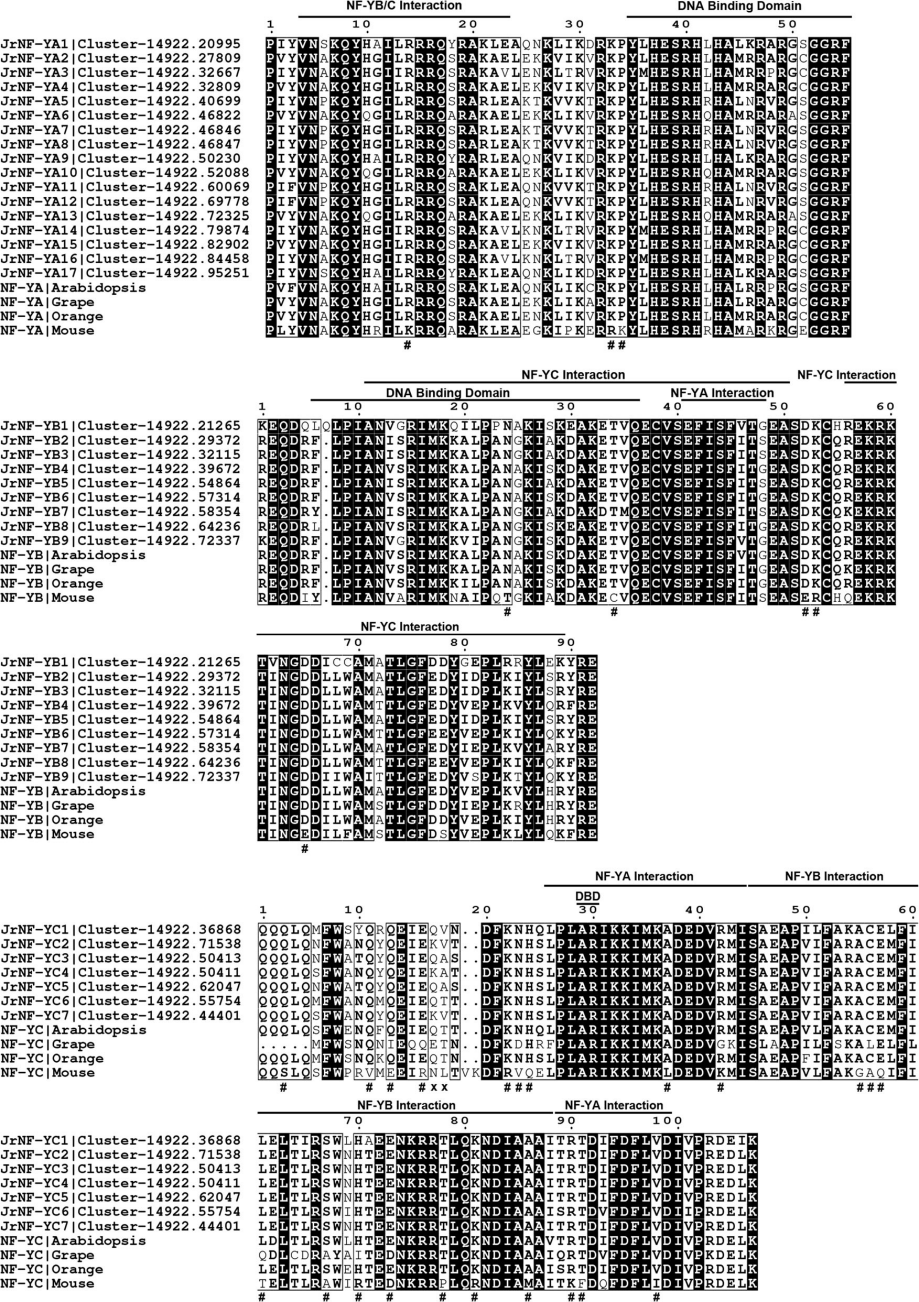
Multiple alignment of the JrNF-Y family was performed by Clustal X. Conserved regions of all JrNF-Y proteins and *Mus musculus* NF-Y proteins (NF-YMouse) are shown. NF-YAMouse, NF-YBMouse, NF-YCMouse were respectively compared with the JrNF-YA, JrNF-YB, JrNF-YC subfamilies. Regions required for DNA binding or interacted with NF-YA, NF-YB and NF-YC subfamilies were previously defined in mammals and yeast. Amino acids in black boxes/white letters were identical in 100% of all aligned sequences. Amino acid sequences with the symbol “#” were conserved in walnut but divided when compared with mouse. Amino acid sequences with the symbol “x” were unique in mouse, and only in the NF-YC subfamily

In most eukaryotes there is a clear boundary between the conserved and non-conserved regions of NF-Y proteins [11, 28]. Studies in yeast have found that CBF-B (NF-YA) and CBF-C (NF-YC) subfamilies are both often accompanied by large amounts of glutamine and some hydrophobic residues, which are involved in transcriptional activation [72]. The conserved regions in the *Jr*NF-Ys were similar; however, there were obvious differences among them (Fig. 2, Additional file 1). Even within the same subfamily, the NF-Y protein sequences showed variability. Therefore, the transcriptional activities of the transcription factors also need to be verified.

Multiple sequence alignment of the conserved regions in the three subfamilies showed that 31 of 55 amino acid residues in the conserved region of NF-YA were absolutely conserved compared with 58 of 92 residues in NF-YB and 86 of 107 residues in NF-YC. These values were much greater than those in Arabidopsis, which had 24 of 53 residues absolutely conserved in NF-YA, 9 of 100 residues absolutely conserved in NF-YB, and 4 of 90 residues absolutely conserved in NF-YC [11]. The conserved regions of the *Jr*NF-YA and *Jr*NF-YB subfamilies were remarkably consistent with the NF-YA and NF-YB subfamilies in grape, orange and mouse. Comparing the conserved regions in walnut with those in mouse, 22 of 86 residues were different in NF-YC, 3 of 31 residues were different in NF-YA, and 5 of 58 residues were different in NF-YB.

The above results indicate that a less proportion of residues were conserved in *Jr*NF-YC than in *Jr*NF-YA and *Jr*NF-YB. The valine(V) and lysine(K) between the nineteenth and twentieth amino acids were unique in mouse and were not observed in seven *Jr*NF-YC sequences and other plant NF-YC sequences (V and K were not numbered and were marked with an “X” at the bottom of NF-YC sequences in Fig. 2). This may reflect the difference between animal and plant. It is worth noting that the initial position of the NF-YC domain reported in Arabidopsis was identified at the L locus whereas the initial position of the *Jr*NF-YC domain was 25 amino acids before the L locus [11].

**Fig. 3:**
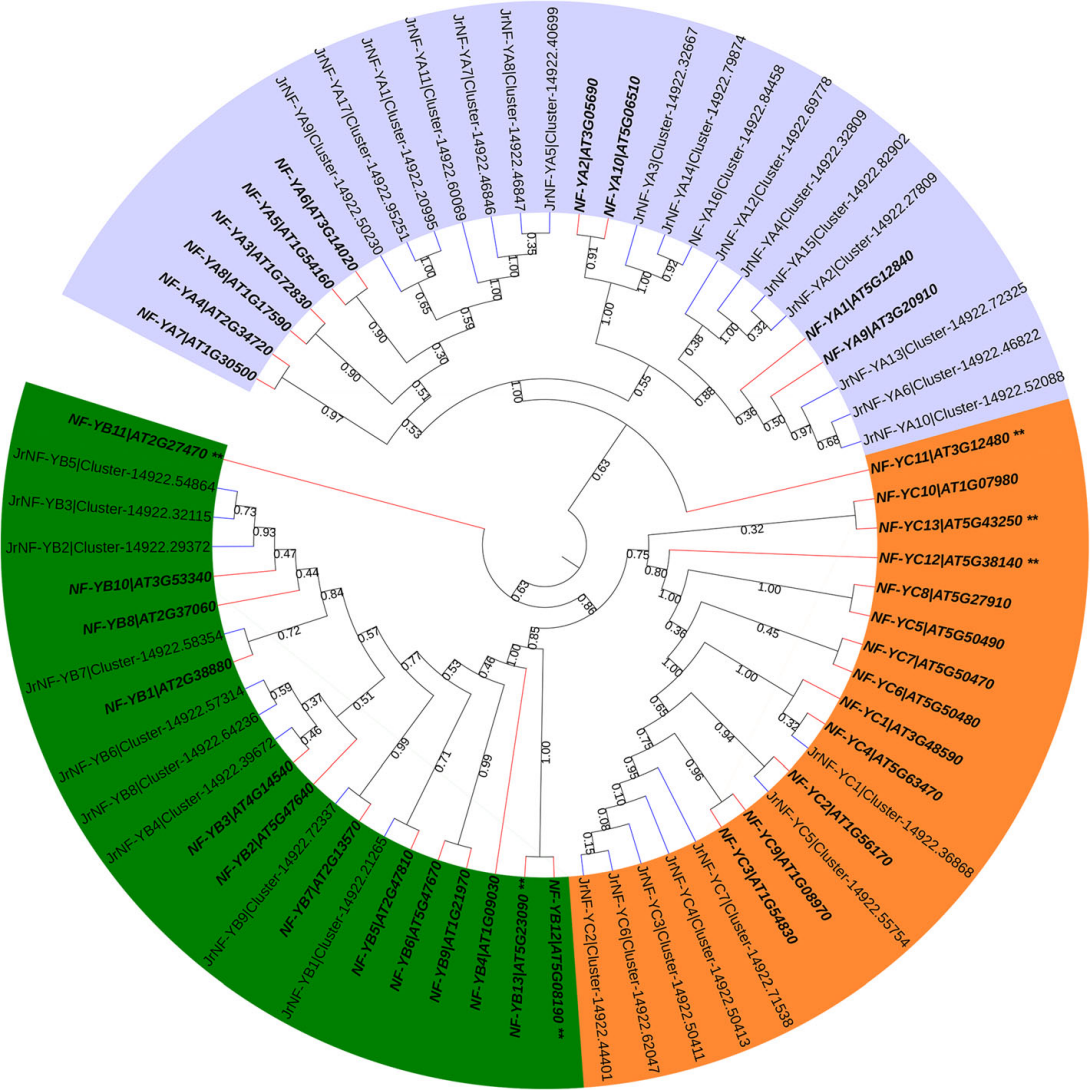
Phylogenetic analysis of NF-Y proteins in walnut and Arabidopsis. The phylogenetic tree was constructed by protein sequences of thirty-three NF-Ys in walnut (JrNF-Ys) and thirty-six NF-Ys in Arabidopsis [11]. Some reports indicate only 30 NF-Y proteins in Arabidopsis [3, 12]. ** indicate NF-Ys reported by Siefers et al. [11] but not reported by either Zhao et al. [3] or Petroni et al. [12]. Purple, green and orange indicate the NF-YA, NF-YB, and NF-YC subfamilies, respectively. The nodes of walnut and Arabidopsis are indicated by blue and red lines, respectively. The bootstrap values are shown on branches

To investigate the evolutionary relationship between the walnut NF-Y family and the Arabidopsis NF-Y family, an un-rooted phylogenetic tree was constructed using the NF-Y protein sequences of Arabidopsis and walnut (Fig. 3). The phylogenetic tree showed close relationships among the candidate NF-Ys within each of the three subfamilies. The exceptions were *Jr*NF-YC1 and *Jr*NF-YB11. The close evolutionary relationships indicated that the NF-Y protein family in Arabidopsis has similar structure and function to that in walnut.

**Fig. 4:**
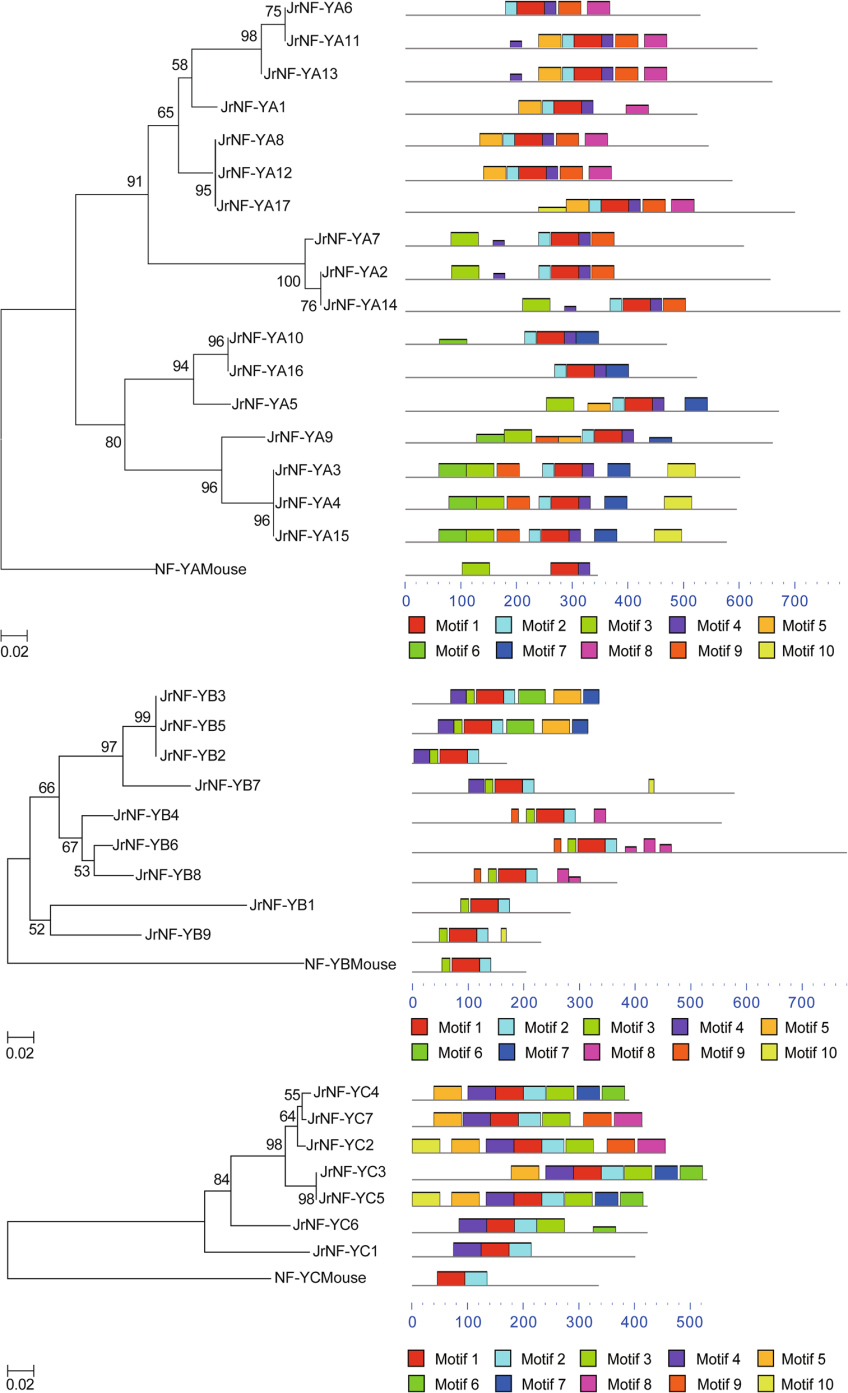
Phylogenetic trees and motif distributions of NF-YA, NF-YB, NF-YC subfamilies in walnut proteins and mouse proteins. Phylogenetic trees were constructed with the conserved regions shown in Fig. 2. The bootstrap values (> 50%) are shown at each branch (1000 replicates). Motif distributions were predicted with the full-length protein sequences of walnut and mouse. The motifs are distinguished by different colors as shown in the legend

A rooted phylogenetic tree of each *Jr*NF-Y subfamily was generated with the conserved domain sequences (Fig. 4). The sequences of the three NF-Y subfamilies in mouse were used as the roots of the phylogenetic trees. The multiple sequence alignment results of each *Jr*NF-Y domain were then used to construct an adjacent evolutionary tree. MEME software (http://meme-suite.org/tools/meme) was used to predict the motif distributions with the full-length protein sequences of the three *Jr*NF-Y subfamilies (Fig. 4) [73]. Initially, the construction of the adjacent evolutionary tree and the prediction of the motif distributions were done separately. Then we observed that the two parts showed some important relationships. For example, the evolutionary relationships indicated close genetic relationships among *Jr*NF-YA3, *Jr*NF-YA4, and *Jr*NF-YA15. Furthermore, their motif distributions were similar. Although the motif distributions were predicted by the *Jr*NF-Y sequence (full-length) and the phylogenetic tree was constructed using domain sequences (fragments), the two results were in good agreement. This phenomenon was also observed in Arabidopsis [11].

### Function prediction and protein interaction

Because of the lack of relevant data about walnut proteins, we predicted the function of *Jr*NF-Y proteins based on corresponding NF-Y proteins in Arabidopsis [68]. We used the Blastp program to align the 33 walnut NF-Y proteins with 36 Arabidopsis NF-Y proteins [69]. Each *Jr*NF-Y protein was closely aligned to at least one Arabidopsis NF-Y protein. Some of the *Jr*NF-Y proteins were closely aligned to the same Arabidopsis NF-Y protein. Overall, the 33 *Jr*NF-Y proteins were most closely aligned to 11 Arabidopsis NF-Y proteins (NF-YA1/NF-YA3/NF-YA9/NF-YA10/NF-YB3/NF-YB5/NF-YB7/NF-YB8/NF-YC1/NF-YC2/NF-YC9) (Table 2).

**Table 2:** Annotation of the JrNF-Y gene family

Gene name	Putative Arabidopsis Orthologs	Function in Arabidopsis
JrNF-YA1	NF-YA1	Flowering time regulation [30]; Salt stress response [54]; Male gametogenesis, embryogenesis, and seed development [39].
JrNF-YA8		
JrNF-YA11		
JrNF-YA12		
JrNF-YA13		
JrNF-YA17		
JrNF-YA3	NF-YA3	Early embryogenesis [41]; Abiotic stress tolerance [55].
JrNF-YA4		
JrNF-YA5		
JrNF-YA9		
JrNF-YA10		
JrNF-YA15		
JrNF-YA16		
JrNF-YA6	NF-YA9	Male gametogenesis, embryogenesis, and seed development [39] [43].
JrNF-YA2	NF-YA10	Root growth [34]; Seed germination [43].
JrNF-YA7		
JrNF-YA14		
JrNF-YB4	NF-YB3	Flowering time regulation [25]; ER stress response [49]; Heat stress response [58].
JrNF-YB6		
JrNF-YB8		
JrNF-YB1	NF-YB5	Unknown
JrNF-YB9	NF-YB7	embryo development [36, 42]
JrNF-YB2	NF-YB8	Unknown
JrNF-YB3		
JrNF-YB5		
JrNF-YB7		
JrNF-YC1	NF-YC1	Flowering time regulation [8]; Freezing stress resistance [56].
JrNF-YC3		
JrNF-YC6	NF-YC2	Photooxidative stress response and flowering time regulation [31]; ER stress response [49].
JrNF-YC2	NF-YC9	Flowering time regulation [26, 28].
JrNF-YC4		
JrNF-YC5		
JrNF-YC7		

**Fig. 5:**
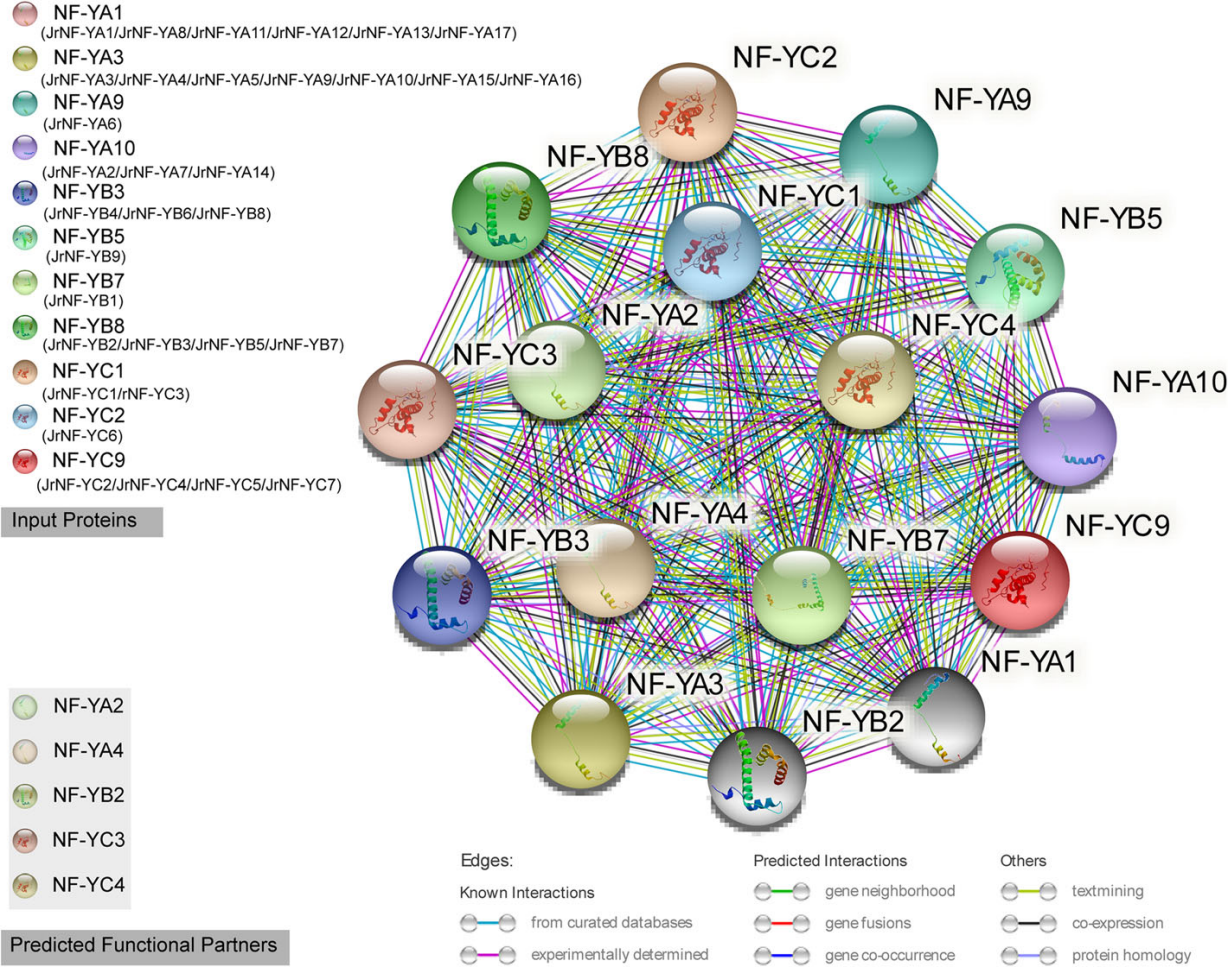
Interaction network of JrNF-Y proteins. The interaction network of the JrNF-Y proteins was constructed using homologous NF-Y proteins in Arabidopsis. The correspondence between the walnut NF-Y proteins (JrNF-Ys) and the Arabidopsis NF-Y proteins (identified in Table 2) are listed in the upper left part of the figure. The network was constructed using the input proteins and six predicted functional partners of the input proteins. The network nodes represent proteins. The 3D structure of the proteins is shown inside the nodes. Edges represent protein-protein associations. The associations are meant to be specific and meaningful, i.e. proteins jointly contribute to a shared function; this does not necessarily mean they are physically bound to each other. The colors of the line indicate different data sources (https://string-db.org/) [74]

In order to investigate the interaction between the 33 *Jr*NF-Ys, we uploaded the 11 Arabidopsis NF-Y proteins which represented the 33 JrNF-Y proteins to the String website [74]. The interaction networks were mapped out according to the 11 input proteins and their 5 predicted functional partners (Fig. 5). The 11 input proteins were annotated to the common function of stimulating the transcription of various genes by recognizing and binding to a CCAAT motif in promoters. Besides, other functions were annotated to these proteins, such as regulation of timing of transition from vegetative to reproductive phase (NF-YA1), embryo development (NF-YA9), long-day photoperiodism and flowering (NF-YB2), positive regulation of transcription (NF-YA3/ NF-YA3/NF-YA10/NF-YB3/NF-YB5/NF-YB7/NF-YB8/NF-YC1/NF-YC2), abscisic acid-activated signaling pathway (NF-YB6/NF-YC9). In addition, the interaction in NF-YA1&NF-YC3, NF-YA1&NF-YC9, NF-YC2&NF-YB3, NF-YC3&NF-YB2, NF-YC3&NF-YB3, NF-YC9&NF-YB2, NF-YC9&NF-YB3 have been validated by lab experiments (https://string-db.org/).

### Expression patterns of *JrNF-Ys* in female flower buds and leaf buds

We compared the relative expression (FPKM) of the 33 *JrNF-Y* genes in F_1, F_2, F_3 and JRL, heat maps were constructed and cluster analysis were conducted to compare the expression (FPKM) patterns of the 33 *JrNF-Y* genes in F_1, F_2, F_3 and JRL.

**Fig. 6:**
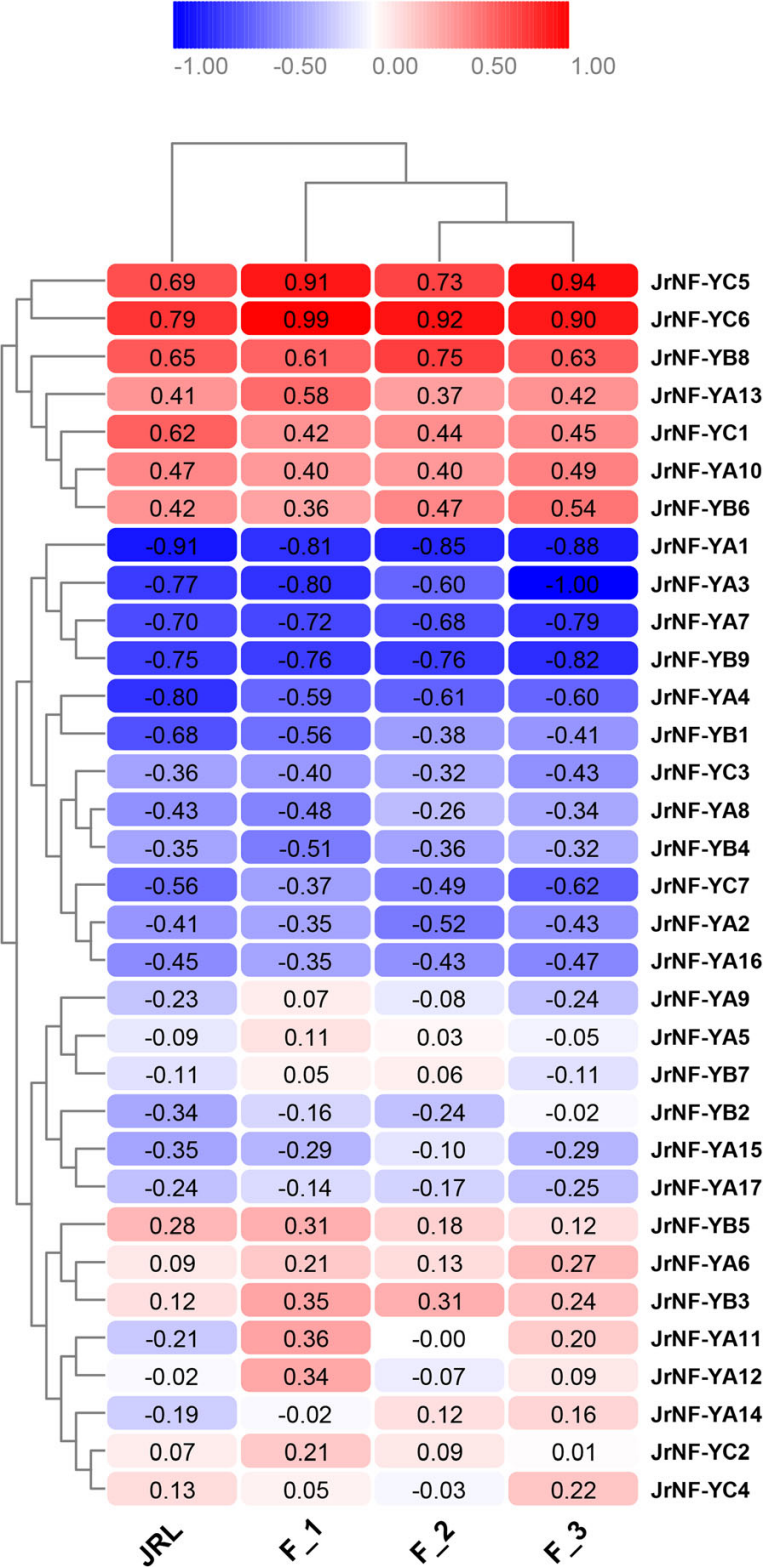
Expression patterns of JrNF-Ys at different development stages and in different walnut tissues. The FPKM values obtained from transcriptome sequencing were used to represent the relative expression level of JrNF-Ys [76]

Cluster analysis showed that the leaf buds (JRL) have distant relationship with female flower buds (F_1, F_2, and F_3). The relative expressions of seven *JrNF-Y* genes (i.e., *A10/A13/B6/B8/C1/C5/C6*) were clustered together for their high expression in F_1, F_2, F_3 and JRL. In contrast, twelve *JrNF-Y* genes (i.e., *A1/A2/A3/A4/A7/A8/A16/B1/B4/B9/C3/C7*) were clustered together for their low expression in F_1, F_2, F_3 and JRL (Fig. 6).

*JrNF-YA11* (q valueF_1vsJRL = 0.001, log2ratioF_1vsJRL = 2.02) and *JrNF-YA12* (q valueF_1vsJRL = 0.046, log2ratioF_1vsJRL = 1.21; q valueF_1vsF_2 = 0.023, log2ratioF_1vsF_2 = 1.47) were screened out for their differential expression patterns. The expression of *JrNF-YA11* was up-regulated in female flower buds before flower transition (F_1) compared with that in leaf buds during flower transition (JRL) (Fig. 6). The expression of *JrNF-YA12* in female flower buds before flower transition (F_1) was upregulated compared with that in (i) female flower buds during flower transition (F_2) and (ii) leaf buds during flower transition (JRL).

**Fig. 7:**
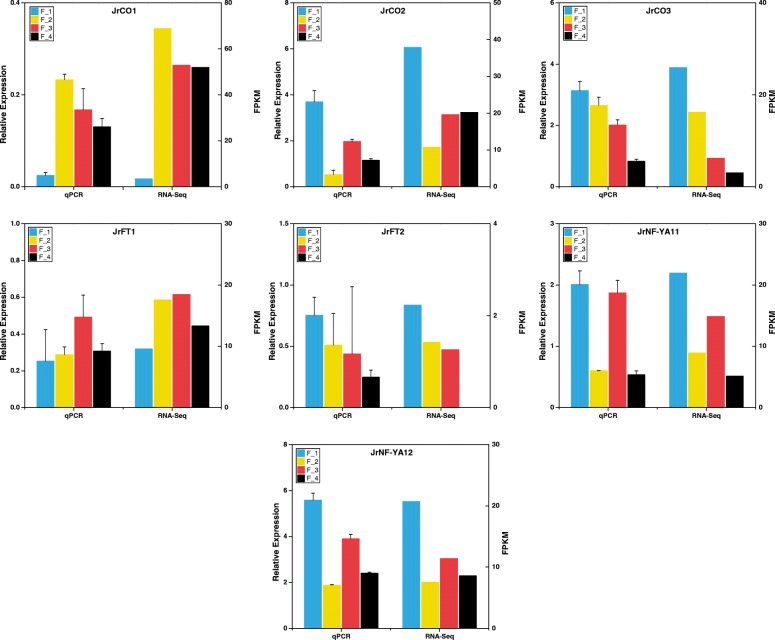
Expression profile of *JrCOs*, *JrFTs* and differential expressed *JrNF-Ys*. Relative expression levels of two differential expressed *JrNF-Ys* (*JrNF-YA11, JrNF-YA12*), three *JrCOs* (*JrCO1, JrCO2, JrCO3*), and two *JrFTs (JrFT1*, *JrFT2*) were obtained from qPCR experiments and were used to construct a heatmap. The data normalization was performed with the computing method of log10 + 2

Some studies indicate that the transcription factor CO competes with other transcription factors (TFs) to regulate the expression of the *FT* gene [3]. We selected two walnut *FTs* (*JrFT1* and *JrFT2*) and three walnut *COs* (*JrCO1*, *JrCO2*, and *JrCO3*) from the transcriptome sequencing data (Additional file 2). The relative expressions of the *JrFTs*, the *JrCOs*, and the differentially expressed *JrNF-Ys* were determined by qPCR (Fig. 7). The expression pattern of *JrCO2* was similar to that of *JrNF-YA11* and *JrNF-YA12*, and their expression trend were down-up-down in F_1, F_2, F_3 and JRL. The similarities also exist between the expression pattern of *JrCO3* and *JrFT2*, and their expression trend were continuous decline in F_1, F_2, F_3 and JRL.

**Fig. 8:**
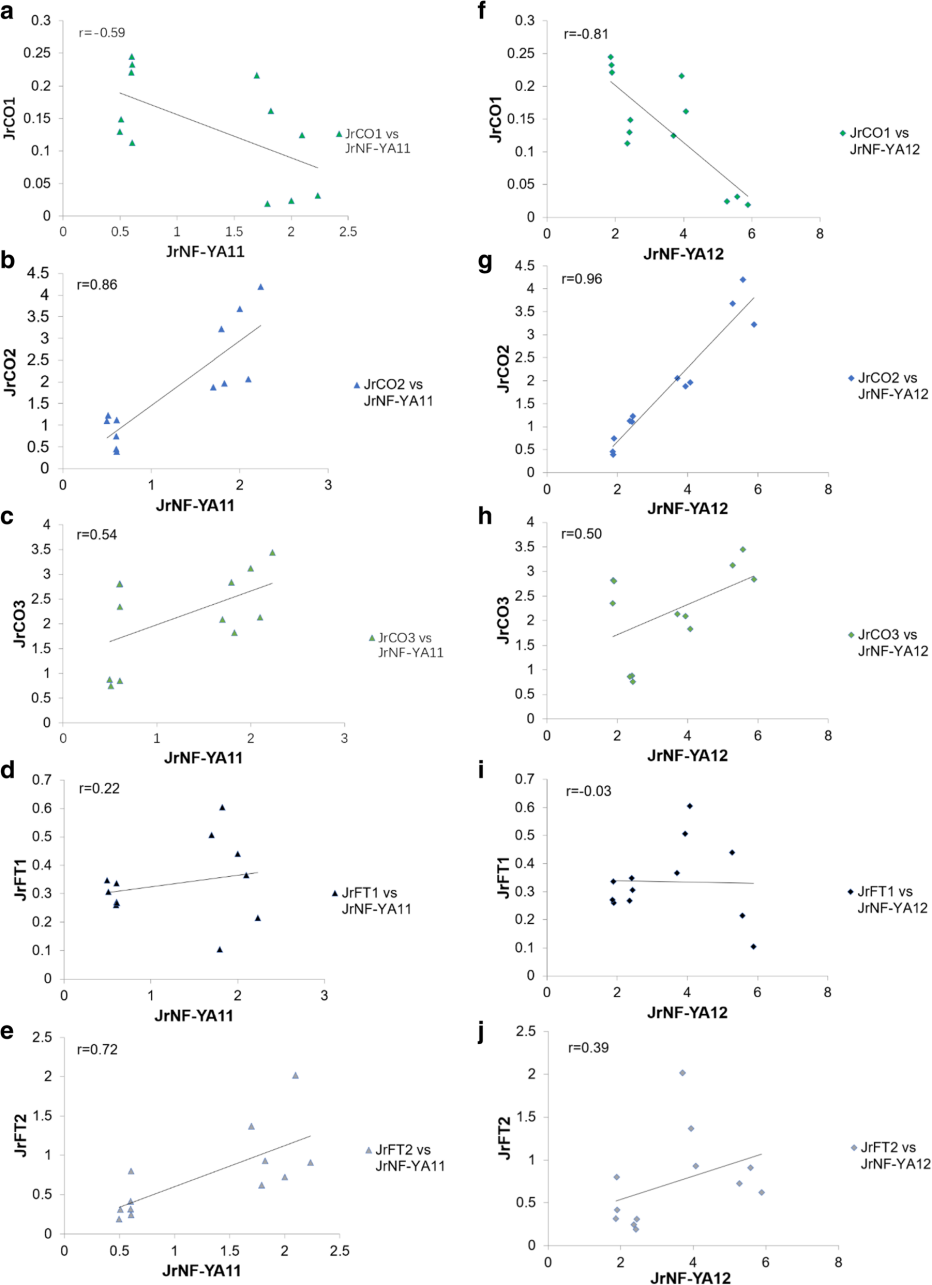
Correlations in expression levels between *JrCOs*, *JrFTs* with *JrNF-Ys* at different periods and in different tissues of walnut. Relative expression levels were used for linear regression analysis. Panels a, b, c, d, e, are for the correlation about *JrCO1* vs *JrNF-YA11*, *JrCO2* vs *JrNF-YA11*, *JrCO3* vs *JrNF-YA11*, *JrFT1* vs *JrNF-YA11*, and *JrFT2* vs *JrNF-YA11*, respectively. Panels f, g, h, i, j, are for the correlation about *JrCO1* vs *JrNF-YA12*, *JrCO2* vs *JrNF-YA12*, *JrCO3* vs *JrNF-YA12*, *JrFT1* vs *JrNF-YA12*, and *JrFT2* vs *JrNF-YA12*, respectively. The correlation coefficient are shown in the figure

The Pearson Correlation Coefficients among these genes is shown in Fig. 8. *JrCO2* showed good correlation with *JrNF-YA11* (*r* = 0.86) and *JrNF-YA12* (*r* = 0.96). *JrNF-CO1* was negatively correlated with *JrNF-YA12* (*r* = − 0.81). *P*-value analysis (Additional file 3) showed that P _*JrCO2* vs *JrNF-YA11*_ = 0.239 > 0.05, which indicated there were no significant difference between *JrCO2* and *JrNF-YA11* and validated the correlation between *JrCO2* and *JrNF-YA11.* However, P _*JrCO2* vs *JrNF-YA12*_ = 0.003 < 0.05, P _*JrNF-CO1* vs *JrNF-YA12*_ = 0.032 < 0.05, which cannot support the correlation between *JrCO2* and *JrNF-YA12*, and the correlation between *JrCO1* and *JrNF-YA12*.

## Discussion

The cDNA sequences of the *JrNF-Y* genes were aligned with the walnut genome. The cDNAs that were mapped to the genome segments were considered as the exon regions (e.g., the section between a1 and b1, Fig. 1c). Considering the post- transcriptional processing, we did not judge the adjacent regions (e.g., NW_017389264.1 44,361 to 44,488; Fig. 1c) to be intron regions even though the possibility exists. We only recorded the information about the start and end sites where cDNA matched the genomic scaffold segments (Table 1).

Thirty-six NF-Y protein sequences of Arabidopsis were used to construct a phylogenetic tree with thirty-three NF-Y protein sequences of walnut. Some studies suggest that six NF-Ys (i.e., NF-YB11/12/13 and NF-YC10/11/13) should not be included in the Arabidopsis NF-Y family because they do not include the proper structure [3]. Our phylogenetic tree seems to support this view (Fig. 3). The six NF-Ys of Arabidopsis have a distant evolutionary relationship with the three clusters of NF-YA/B/C. None of the 33 *Jr*NF-Ys was included in the same sub-cluster with the six Arabidopsis NF-Ys mentioned above.

There are obvious differences between NF-Y proteins in animals and plants (Fig. 2). Two amino acids were observed in mouse NF-Y sequences but not in plants NF-YC sequences. However, the evolutionary conservation of NF-Y proteins in mouse and plants was also demonstrated. The conserved region of NF-Y proteins in mouse and plants showed high similarity in all three subfamilies. In previous report [12], NF-YC conserved regions in Arabidopsis strart form the leucine (L) at the twenty-sixth site (Fig. 2). Sequence alignment indictaed that the 25 amino acid residues before the NF-YC conserved regions in previous report were consistent between mouse, Arabidopsis, walnut and orange.

Conservation and differentiation also exists between plants. Absolutely conserved sequences in black boxes/white letters were shared by Arabidopsis, walnut, grape and orange. However, the first five amino acids (QQQLQ) of NF-YC (Fig. 2) were missing in NF-YC sequences of grape, and this situation did not exist in Arabidopsis, walnut and orange.

An interaction network among the 33 *Jr*NF-Y proteins was established based on the 11 correlated Arabidopsis NF-Y proteins. The 11 input proteins were annotated to the function of regulation of timing of transition from vegetative to reproductive phase, embryo development, long-day photoperiodism and flowering, positive regulation of transcription, abscisic acid-activated signaling pathway. In addition, seven protein-protein interactions have been validated by lab experiments and other interaction relationships were predicted (https://string-db.org/). There is no doubt that the network provides valuable information for further research.

With the exception of *JrNF-YA10*, the expression of the *JrNF-Y* genes in female flower buds varied among development stages (i.e., before, during, and after flower transition, Fig. 6). This suggests that the *NF-Y* genes directly or indirectly participate in the process of flower bud development. Previous studies have confirmed or predicted that most NF-Ys are involved in the regulation of flowering time [3, 11, 23–33]. This is also supported by our observation that the expression of 24 of 33 *NF-Y*s was greater in female flower buds (F_2) than in leaf buds (JRL) (Fig. 2). The expression of nine *NF-Ys* was greater in leaf buds than in female flower buds. It is possible that these *NF-Ys* inhibit flowering during the vegetative stage and this need more experimental evidence.

Previous studies have indicated that CO and NF-Y compete to regulate *FT* expression in Arabidopsis [3]. The NF-YA and CO proteins both can combine with an NF-YB-YC dimer to form either (i) an NF-YA-YB-YC trimer which inhibits *FT* expression or (ii) an NF-YA-YB-CO trimer which promotes *FT* expression. In the photoperiodic pathway of Arabidopsis, NF-YA expression is greatest during the day, whereas *CO* expression is greatest at night. The expression of *FT* reflects this diurnal pattern. Specifically, expression of *FT* is low during the day (when *NF-YA* expression is high) and high during the night (when *CO* expression is high). We observed that *JrCO1* expression was negatively correlated with the expression of both *JrNF-YA11* and *JrNF-YA12*. However, *JrFT2* was positively rather than negatively correlated with *JrNF-YA11* and *JrNF-YA12* in female flower buds (i.e., F_1, F_2, F_3) and in leaf buds (i.e., JRL). *JrNF-YA11* and *JrNF-YA12* had greater expression in female flower buds during flower transition (F_2) and leaf buds (JRL) than in flower buds before flower transition (F_1) or after flower transition (F_3). A complex network is involved in the regulation of flowering. The expression of *FT* is regulated by many transcription factors. However, the results suggest that *Jr*CO or other TFs compete with *Jr*NF-YA proteins to combine with the *Jr*NF-YB-YC dimer and promote the expression of *JrFT2*. This hypothesis needs to be tested in future research work.

## Conclusions

Thirty-three *JrNF-Ys* were identified and their evolutionary, structural, and biological functions were analyzed. The biological function of the *Jr*NF-Y proteins was predicted by comparative analysis with Arabidopsis NF-Y proteins, and this provided a rudimentary understood for the less-studied *JrNF-Ys*. Further more, Two *JrNF-Ys* were differentially expressed during the process of flower transition, which revealed that *JrNF-Ys* might play a role in flower transition. The results of this study may contribute to the future studies about the *JrNF-Y* family.

## Methods

### Plant materials

Walnut (*J. regia* L.) trees were grown under natural conditions in the southern part of the Xinjiang Uyghur Autonomous Region, China. Leaf buds were collected during the flower transition period (JRL) and female flower buds were collected before, during, after the flower transition period (F_1, F_2 and F_3). The leaf buds(JRL) were collected at the same period during the flower transition(F_2). The samples were immediately frozen in liquid N and stored at − 80 °C.

### Transcriptome sequencing and de novo assembly

Solexa/Illumina sequencing was carried out by Novogene, Beijing, China. Total RNA was extracted from three female flower buds at each stage (i.e., F_1, F_2, and F_3). Total RNA was extracted from 18 leaf buds (JRL). Total RNA was extracted using RNAout 1.0 (Tianenze, Beijing, China). A total of 1.5 μg RNA per sample was used as input material for the RNA sample preparations. Sequencing libraries were generated using NEBNext ® Ultra™ RNA Library Prep Kit for Illumina ® (NEB, USA). The clustering of the index-coded samples was performed on a cBot Cluster Generation System using TruSeq PE Cluster Kit v3-cBot-HS (Illumia). After cluster generation, the library preparations were sequenced on an Illumina Hiseq 2000 platform and paired-end reads were generated. For the assembly library, clean data(clean reads) were obtained by removing reads containing adapter, reads containing ploy-N and low quality reads from raw data. Clean reads were de novo assembled using Trinity [75], and the transcriptome reference database was obtained. FPKM was used to obtain the relative expression levels [76].

### Identification of *JrNF-Ys*

The protein sequences of 36 *NF-Y* genes (10 *NF-YA* genes, 13 *NF-YB* genes, and 13 *NF-YC* genes) in Arabidopsis were downloaded from TAIR (http://www.arabidopsis.org/) (Additional file 4) [11]. These sequences were used to search our walnut transcriptome database (unpublished) with the tblastn program in BLAST (blast-2.60) [69]. The screening threshold was set as 1e-10. The protein sequences of 10 *NF-YA* genes, 13 *NF-YB* genes, and 13 *NF-YC* genes were used to establish three Hidden Markov Models (HMMs) (Additional file 5) [70]. The three models were used as the query to search the transcriptome database with the screening threshold set at 1e-10. The results of the BLAST and HMMER searchers were merged, resulting in 104 candidate NF-Y genes in walnut.

All 104 candidates were uploaded to the NCBI to verify the existence of the core domain using Conserved Domain Search (https://www.ncbi.nlm.nih.gov/Structure/cdd/wrpsb.cgi) Some candidates were abandoned because they lacked the core domain. Other candidates were abandoned because their sequences were either too long or too short. Finally, 33 unigenes were identified and translated into amino acid sequences (Additional file 6).

### Multiple alignments and phylogenetic analyses

Clustal X 2.1 [71] was used to align the protein sequences of the JrNF-Y genes. The conserved regions of the three subfamilies were identified using Arabidopsis as a reference. The conserved domains of three subfamilies in Arabidopsis, walnut, grape, orange and mouse were uploaded to the ESPript website (http://espript.ibcp.fr/ESPript/cgi-bin/ESPript.cgi) for editing [77]. The three subfamily sequences of Arabidopsis, grape and orange were download from the website of PlantTFDB (http://planttfdb.cbi.pku.edu.cn/) [78], and then HMM model of NF-YA, NF-YB, NF-YC of Arabidopsis, grape and orange were built based on these sequences (Additional file 5).

Protein sequences of NF-Y genes in Arabidopsis and walnut were used to construct a neighbor-joining tree with 1000 bootstrap replications using MEGA 6 software [79]. The phylogenetic tree constructed by MEGA was uploaded to iTOL (http://itol.embl.de/) for further editing. Motifs were predicted using MEME software (http://meme-suite.org/tools/meme). A protein interaction network was constructed with String software (https://string-db.org/) [74].

### Quantitative real-time PCR

Total RNA was extracted using RNAout 1.0 (Tianenze, Beijing, China) by Novogene, Beijing, China. The synthesis of cDNA was performed using a PrimeScript RT Reagent Kit (TaKaRa, Dalian, China). Real-time quantification was performed using a CFX manager (Bio-Rad, USA) with the SYBR Green Realtime PCR Master Mix (Toyobo, Osaka, Japan). The protocol of the real-time PCR was as follows: initiation with 95 °C for 5 min, followed by 40 cycles for 30 s at 94 °C, 30 s at 55 °C, and 30 s at 72 °C. A melting curve was included from 65 to 95 °C to verify the specificity of the amplified product. Each reaction was repeated three times. Walnut actin gene (forward: 5′-CCATCCAGGCTGTTCTCTC-3′, and reverse: 5′-GCAAGGTCCAGACGAAGG -3′) and walnut gadph gene (forward: 5′-ATTTGGAATCGTTGAGGGTCTTATG-3′ and reverse: 5′- AATGATGTTGAAGGAAGCAGCAC-3′) were used as the normalizer (Additional file 7). The results were evaluated by the method of the 2 ^-ΔCt^ [80].

### Differential expression analysis

Prior to differential gene expression analysis, the read counts for each sequenced library were adjusted with EdgeR software. Differential expression analysis of two samples was performed using the DEGseq (2010) R package. The thresholds for significant differential expression were qvalue < 0.05 and |log2(foldchange)| > 1.

## Additional files

Additional file 1: Figure S1. The conserved regions in the full length of the JrNF-Ys. (DOC 2960 kb)

Additional file 2: Unigene sequences of JrCOs and JrFTs. (DOC 36.0 kb)

Additional file 3: **Table S1**. Correlation and P-value in Expression Level. (DOC 40.0 kb)

Additional file 4: Full length and conserved sequences of the Arabidopsis and mouse NF-Ys. (DOC 44.0 kb)

Additional file 5: The HMM models and domain sequences of NF-YA, NF-YB and NF-YC of Arabidopsis, grape and orange. (DOC 44.0 kb)

Additional file 6: Unigene sequences and translated amino acid sequences of 33 walnut NF-Ys. (DOC 116 kb)

Additional file 7: Primers involved in this article. (DOC 32.0 kb)

## Abbreviations

CBF: CCAAT binding factor
*CO*: *CONSTANS*
ER: Endoplasmic reticulum
F_1: Female flower buds before flower transition
F_2: Female flower buds during flower transition
F_3: Female flower buds after flower transition
*FT*: *FLOWERING LOCUS T*
HAP: Heme activator protein
HMMs: Hidden Markov Models
JRL: Leaf buds during flower transition
qRT-PCR: Real-time quantitative PCR
TFs: Transcription factors
