# Supplementary material for: Identification and characterization of NF-Y gene family in walnut (Juglans regia L.)
Source: BMC Plant Biol. 2018 Oct 23;18:255. doi: 10.1186/s12870-018-1459-2 (PMC6199752; doi:10.1186/s12870-018-1459-2)
Supplement: Supplementary file 1 — Figure S1. The conserved regions in the full length of the JrNF-Ys. (DOC 2960 kb) [file 12870_2018_1459_MOESM1_ESM.doc]

**Additional file 1: Figure s1.** The conserved regions in the full length of the JrNF-Ys

**Figure s1.** The conserved regions in the full length of the JrNF-Ys








**The conserved regions in the full length of the JrNF-YAs**





**The conserved regions in the full length of the JrNF-YBs**





**The conserved regions in the full length of the JrNF-YCs**
