# Supplementary material for: Identification and characterization of NF-Y gene family in walnut (Juglans regia L.)
Source: BMC Plant Biol. 2018 Oct 23;18:255. doi: 10.1186/s12870-018-1459-2 (PMC6199752; doi:10.1186/s12870-018-1459-2)
Supplement: Supplementary file 2 — Unigene sequences of JrCOs and JrFTs. (DOC 36.0 kb) [file 12870_2018_1459_MOESM2_ESM.doc]

**Additional file 2:** Unigene sequences of *JrCOs* and *JrFTs*.

These are the unigene sequences of *JrCOs* and *JrFTs.*

>JrCO1|Cluster-14922.102174

CACACCCACTTCATTCACTGTGTTCACAGTTCACACACAAATACTCAAAAACTCAGAGCCACAGCGAGAGAGAAAAGATA

GAGAGATATGATGAAGGAGGAGATTAGTAATGGTGGTGATGGCGGTGGTGGCAATAACTGGGCACGTGTATGCGACACGT

GCAGGTCGGCGGCTTGCACAGTGTATTGCCGGGCCGACTCGGCCTACCTGTGCACCGGGTGCGACGCCCGCATCCACGCC

GCGAACCTCGTTGCGTCCCGCCACGAGCGTGTGTGGGTCTGCGAGGCGTGCGAGCGCGCCCCGGCAGCTTTCATATGCAA

AGCCGACGCCGCGTCTCTCTGCACCGCCTGCGACGCCGACATCCACTCCGCGAACCCACTCGCAAGCCGCCACCACCGCG

TCCCAATTCTCCCCATCTCCGGGTGCTTACCCGGCCCACCTGCTGCTGACCCCGTAGACGGGTTTTTAGCCCATGATGAT

GGGGACGAGACCATTGACGAAGAGGATGAAGATGAGGCAGCATCGTGGTTGTTACTCAATCCCGTGAAGAATAACTACAA

TCAGAACAATGGGTTCTTGTTTGGTGGGGATGTTGAAGAGTATGTGGACTTTGTGGATTACAACTCGTGTGCTGATCAGA

ATCAGTTCGAAGATCAGTATAACTACGACCAGCAACAACATTACGCTGTTCCCCAGAAGAGCTATGGGGGTGACAGCGTT

GTGCCCGTTCAGAATGGAGATGTGAAGGTTCAGCTGCGTCACCAGCCGCAACAACAGGATTTTCAGTTGGGACTGGATTT

TGAGTCCTCAAAATCTACATACAGTTACCATGGTTCCATTAGTCACAGTGTCTCTGTTTCATCTATGGATGTTGGCGTTG

TACCAGAATCAACGATGATCGATATCTCAATCTCACACCCAAGAACTCCCAAAGGGACAATTGACCTTTTCTCTGGACCT

CCTCTCCAGATGCCACACCAACTTACCCCACTGGACAGGGAAGCAAGGGTCCTAAGATACAGAGAGAAAAAGAAGACAAG

GAAGTTTGAGAAAACAATCCGGTATGCTTCTAGGAAGGCATATGCAGAGACCAGACCCCGGATCAAGGGCCGTTTTGCTA

AGAGGACTGATGTAGAAGTTGAAGTGGACCAGATGTTTTCCACCACACTAATGGCAGAAACTGGATATGGCATTGTGCCA

TCATTCTGAGGCTAGTGAGAGAAGAAGAAGAAG

>JrCO2|Cluster-14922.52838

TCTCCTCTCTCTTACCTTTCTTCCCAACGGGTCCAACTCCTGCGGCCCCCACAAAAAGTTGTTCACTTTTTCTCACAATT

TCTTCATTCCCCGGCTTCAACCAGGAAGAGAGATCAGCCTGAGGAAGCCATAGAGTGCCTTGTCTGTGAGTGTGTGAATG

CGCTGAGAGATCATGTGCAACAAGAACAGTCCTCTGGATATGAAAACCAGGTCTCGGACTAAGAAAGAGAAAGCCAGAGC

AACGCTTAAACCAGCACGCAAAACAAGAACCAAGACCAGGAAGCCCAAGTTCCTCAGTCTCCGCCTGGAACTTTCCTCAG

AAAACTCACCAACCTCTTCCCAAATGACGCACAGCCACAAAAACAACCAACACGATGCAGATGATCACAGTAGCCAGCAC

CAGCTGAACCTGTTTCCTCTACACCCGGAAAACCTAGTGGACGACAAAGACATACACGACGAGAACGTGGCCTTCCTCTT

CGACACCGAACCCGGCGCCACCCTTAACAGCCTCCTTGCCGAGGCTTCGACGTCCTCGGAGGATGACTCGCTCTTTTCCC

CGCCGTCACTAACGTACGCCTACGGGGGAAAGGATAGGGATCAGGAGGGTGTTGATGTCGGTGGCAATTACTGTAACAAC

ACGAACTCCAAGCTGGTACGGACGGCCATGAGGAACAAAGAGAGAGAACCGAGTGAGGAGGAGAAGTGGGTGTGTTACTC

GGAGGTACTAGTGGAGAAGAAGGAGCTGGAAGAGGTGAGCAGCTGCACGGCGGACGTATGGCCGCCTTGTGCAATAAAAC

GGACGAAGATGCAGGGGTTGCTGTCATTGAAGCTTGATTATCAGGGCATCTTAAACGCTTGGTCTGGAAAGGGCCCGCTT

TTCATTGCCGGAGAGTCCCCCCCACAGACTGTGCCTGACTTGCACGAGGATCGGTTTTTTGCCCATGACGGTGCCAATAT

TCTAGGGGATGGTTGGGGCAACGTTGGTAACATATGGGCGGTTCCAGAAATGGGTTGCAGTAGTACTGATATTGGTGGCA

TGAAAGTGGAGGAGGAAGCGGAAGGCAAAGATGGTTGGAAGACGAGGCACAGAGAAGCAAGCGTGTTGAGATACAAGGAG

AAGAGGCAGAACAGACTCTTCTCAAAGCGTATCCGATACGAAGTCCGAAAGCTTAATGCTGAGAAGCGACCCCGTATGAA

GGGTCGATTCGTGAAGAGAAGTTGAGAAGGCATAGACTCTTGGACTTTTTCTTTTCCTTGCAACATAGTAGGGGGCACTG

AGATAATGTTAGTCAGTGCCAAAACAAATCCAAATGAATAAAAAAACAAAAAGAAAGTTGTAGACGCACAGAGATTTAAG

TTATAAGGAGTGCCTACTTTGGAATAATGGATGAGATCCATTCCTGTTTGCTTAATTTTCCCATCAATTTACAGAGCCAA

ATTACGTTTATTTGCAGAATGTTTGGGACGTCAGGTTAGGGTGTACTAGTGGAGTTACGATACAATACTATTGGCAAAGT

TAATAGAAAAATGTTACTTTGTCCACTTCATTTTTTGACACTTCATATATTTT

>JrCO3|Cluster-14922.58854

CAACCACTTTAAACACGTATTAGTAGCAAATAAGAAGGCCGTATCGCCATAATGCAACAACAAAATGGAGAGTTATTTGT

TCGTACACTGATGTAAAACCACAAGAGTATTCCCTCAAAAACATATATATAATTCCCTGCTATACCTGTTTCATGAAATG

GAGGAGAAATGTACAAGGAGAGGGTATTTGTCTGACCACGGGATTGTTAACCAGACAAACTCTCTTTTAAGTCATATAAA

TCCTGTGCTATACATGTCAATTTAAAAGGAAACGAGAACAGTAGAGAAGTGCTAGCTAGTTTCTTGGTTGATCTGGCAGA

TGTGAGTACAGCCACCCGCCATGCCATGAAAACTGAATTTGTAGAAGTGACCAGACCGTGGAATACAATAAACAATAGTT

ATTGAGGTGTTAGGGCTGGGGAGAACCCATAAGGTTCTTTCACTAGAGAACTGCGTACAGACAGACAACAATCCATAACT

TGAATCGAGGGTGCCATGCGGTCTGTTCGGATGGTGTCACAACCCACCAATCCTTTATAGTGATGGATGATCGACATTCT

GCTCCTCCACTTATTGGCGAAGCATTGGAAAACAGCATTTTATATCCACCACTCTCCTGATGAAAACCAAGGCCCTCTCA

GTAGCTTCTGGTTTGTGTCAACGGATCATAATCATAAGCATCACCAGCTTTGACAAAGCGTCCCTTAACACGTCTTCTAA

CATCGGCCCTTGCTTTGCGAGAGGCATACCTCACTCTTTTCTCGAACTTGCGTGCTTTCTTCTTTTCCTTATAACGCATA

ACAGCATCATTACGATTAGCAGATTGGAAGGAACTCTCAGGACATGGAGGACACCATGGAGGCTCTCCCATGAGAAGCAT

TGCAGAAGCCCCACAATCTTGATAATCCCCAGGACTACTCTCTCCAGTAAGACCAGAAAACGAAATACTTGAATGTGCTT

GTCTTGTTGTCAAACAAAGTACTGGTTCAGTTTTAGTACTCATCATAGAATCTGCAGACACTGCATTGCTGCACGCTGGC

TGCATTGCATTAACCAGTCCCACTGAGGACCCCTCAGCAGCAACTGCATCCTGACAACTGGAATCAGCAGCAGACATGTC

CTTTGTCCCAAACAAGCTATCAATGCCACCATTTTCAAAAAGCTCTTCAGAATGGCTAAGCGCTACACCAAAGAGTTCTT

CATAGTTCTCAAGATTCAAATCCACTTCATCCATTTCAAAGTCCTCATATAGATCATCTTCTTGACAGAGACCAGAACCT

TTAGCTCCAGGACAGCATATCTTGGGCAAAGATGTATTTGCGGATCCAGCCGGCTGGTCTAGAATGTGAGGCCCAGAATG

AACTCCAGGCACTGAAGAGGACCCAACCCAACCGCTGGACTTCTCGACGTTGCGTACGCCATTCAATTCAACTGAACTAT

ATAAATCTTGGCTCATGTTGTTTTCTGCAGAGCCCCAAGCAGTTTTTTCACTGTTCTCTGTTATACTCATCAAACCTAAT

TCCTGCTCACAAGTGGATTCACCCACAGAAGGGGAATCTAAAACAAATGACCAGATTGAAGAAAGTTCTGCAGCTGATGG

GCATCCAGAATAACAATTGATTGTCTGTCTCTTGTGTGTTGAAGCTGAGGTAGAAGTGCCATGACCCATCCAATCACAGT

TCTGACAAAGAGAGACCCTCTCTTGGGCACATCTTACTAGTGCAGGTTGTGAATTGCATCTTTCACATAATAGTGTTCTC

GAGTGGCGTTTGGATAGGGCATTAGCAGAATGGACATTTCTGTCACATGACAAGCATAAGCATGCAGCATCTGAGCGGCA

ATACACCATGGACCTTTGTTCCCCGCAAAAATCACATAAATAACCCATCTTCAGATTTATACTTCTCCAATTACCAAACC

TCTACCCAAGTTATATATCAGCCTACCAAAAGTAGCTCCTTACACGAATCACAATCCTTCCCAGCGTCGTTACAACCGTT

CAGAAAAGAGGTGGACAAGATCGGTAAAGAACTGAATTTCTAATTAATTATTGTAAGAAGTAGACAAAACTAAGCAACCC

TAAGAACAGGATCTTTCCAGAAAATATCTCTCTCTCTCTCTCTCTCAGTTGCTAAGATATCTGCACTCTGCTCTTGGCGA

CAGAGTAAATGGACAGTGAAGGTGTCTATTT

>JrFT1|Cluster-14922.104730

GAAGCCGATCAGCAAATAAAGACTGCTTGAATATAAGTTTTCAACTTTTAGTGCTGTAGATGGAGGATTGAATTTTTCCA

ATCATGCAAAGGGATAGAGACCCTCTAGTTGTTGGACGTGTTATAGGTGATGTTTTGAACCCCTTTGAGAAGTCCATTGC

TCTGAGGGTCACTTATAACAATAGAGAGGTTAACAATGGCTGTGAGCTCAAGCCGTCTCTTGTTGTCAGCCCACCTAGGG

TTGATGTAGGCGGTGATGACCTCAGGACTTTCTACACTCTGGTTATGGTGGATCCTGATGCACCCAGTCCTAGCGATCCC

AACCTAAGGGAATACTTGCATTGGTTGGTGACTGATATTCCAGCAACTACAGGGGCAAACTTTGGACAAGAAGTTGTGTG

CTATGAAAGCCCACGACCAACAGTGGGGATTCATCGATTTGCTTTCGTCTTGTTTCGCCAATTGGGTAGGCAGACAGTCT

ACGCACCCGGATGGCGCCAGAATTTCAACACCAGGGACTTCGCTGAGCTTTACAACCTTGGATTACCTGTGGCTGCAATC

TATTTCAATTGCCAGAGAGAAAGTGGCTCAGGTGGAAGAAGAAGCTGATCACGAAAAACCCTCTATCTTTGAACATGTAT

TAAACAAGAAACTAGTCTACAGAAGTGTGCGTATGCTTATGGTTTGAATGTGTGAGCTATATATCTATATCTAAATACTT

TGTTACAGATACAA

>JrFT2|Cluster-14922.51737

GAAGCCGATCAGCAAATAAAGACTGCTTGAATATAAGTTTTCAACTTTTAGTGCTGTAGATGGAGGATTGAATTTTTCCA

ATCATGCAAAGGGATAGAGACCCTCTAGTTGTTGGACGTGTTATAGGTGATGTTTTGAACCCCTTTGAGAAGTCCATTGC

TCTGAGGGTCACTTATAACAATAGAGAGGTTAACAATGGCTGTGAGCTCAAGCCGTCTCTTGTTGTCAGCCCACCTAGGG

TTGATGTAGGCGGTGATGACCTCAGGACTTTCTACACTCTGGTTATGGTTGATCCTGATGCACCAAGTCCAAGTGATCCC

AACCTAAGGGAGTACTTGCATTGGTTGGTGACTGATATTCCAGCAACTACAGGGGCAAACTTTGGGCAAGAGGTTGTGTG

CTATGAAAGTCCAAGACCAACTGTAGGGATCCATCGTTTTGTTTTCGTGTTGTTCCGACAACTGGGTAGGCAGACCGTCT

ATGCACCTGGGTGGCGCCAGAATTTTAACACCAGGGAATTTGCTGAGCTTTACAATCTTGGATTGCCTGTGGCTGCACTG

TATTTCAATTGCCAGAGGGAAAGCGGCTCGGGTGGAAGAAGAAGATGACTGATCACGAGGCCTCCAAGGAAACCCTTCTG

TCTATGGAAAAAGAGTTAAATAATTAAGAATCTAGCTAGCTAGTCTATGTGGTGTACTGCAGTACGTATGAGATCAGATA

TATATTGATCTCAACCACATATCTCGTTCTATCTATGTGCTTAGACATTGGGGATAACTGCTAATTATTATTCTTCAGGG

GTGTAAACGAGTTTAATGTATCTCAAATACATTAATCTATATTAATAATAATTAATGAAGTTTAATG
