# Supplementary material for: Identification and characterization of NF-Y gene family in walnut (Juglans regia L.)
Source: BMC Plant Biol. 2018 Oct 23;18:255. doi: 10.1186/s12870-018-1459-2 (PMC6199752; doi:10.1186/s12870-018-1459-2)
Supplement: Supplementary file 3 — Table S1. Correlation and P-value in Expression Level. (DOC 40.0 kb) [file 12870_2018_1459_MOESM3_ESM.doc]

**Additional file 3: Table s1.** Correlation and P-value in Expression Level.

**Correlation and P-value in Expression Level**

|  |  |  |  |  |  |  |
| --- | --- | --- | --- | --- | --- | --- |
| Correlation in Expression Level | JrCO1 | JrCO2 | JrCO3 | JrFT1 | JrFT2 | JrNF-YA12 |
| JrNF-YA11 | -0.59 | 0.86 | 0.54 | 0.22 | 0.72 | 0.89 |
| JrNF-YA12 | -0.81 | 0.96 | 0.50 | -0.03 | 0.39 | * |
|  |  |  |  |  |  |  |
|  |  |  |  |  |  |  |
|  |  |  |  |  |  |  |
|  |  |  |  |  |  |  |
| P-value in  Expression Level | JrCO1 | JrCO2 | JrCO3 | JrFT1 | JrFT2 | JrNF-YA12 |
| JrNF-YA11 | 0.078244 | 0.238753 | 0.13196 | 0.095274 | 0.135927 | 0.02066044 |
| JrNF-YA12 | 0.031818 | 0.002798 | 0.167657 | 0.033467 | 0.035663 | * |
