# Supplementary material for: Identification and characterization of NF-Y gene family in walnut (Juglans regia L.)
Source: BMC Plant Biol. 2018 Oct 23;18:255. doi: 10.1186/s12870-018-1459-2 (PMC6199752; doi:10.1186/s12870-018-1459-2)
Supplement: Supplementary file 4 — Full length and conserved sequences of the Arabidopsis and mouse NF-Ys. (DOC 44.0 kb) [file 12870_2018_1459_MOESM4_ESM.doc]

**Additional file 4:** Full length and conserved sequences of the Arabidopsis and mouse NF-Ys.

These are the protein sequences of Arabidopsis NF-Ys, full length

>NF-YA1|AT5G12840

MQSKPGRENEEEVNNHHAVQQPMMYAEPWWKNNSFGVVPQARPSGIPSNSSSLDCPNGSESNDVHSASEDGALNGENDGTWKDSQAATSSRSVDNHGMEGNDPALSIRNMHDQPLVQPPELVGHYIACVPNPYQDPYYGGLMGAYGHQQLGFRPYLGMPRERTALPLDMAQEPVYVNAKQYEGILRRRKARAKAELERKVIRDRKPYLHESRHKHAMRRARASGGRFAKKSEVEAGEDAGGRDRERGSATNSSGSEQVETDSNETLNSSGAP*

>NF-YA2|AT3G05690

MAMQTVREGLFSAPQTSWWTAFGSQPLAPESLAGDSDSFAGVKVGSVGETGQRVDKQSNSATHLAFSLGDVKSPRLVPKPHGATFSMQSPCLELGFSQPPIYTKYPYGEQQYYGVVSAYGSQSRVMLPLNMETEDSTIYVNSKQYHGIIRRRQSRAKAAAVLDQKKLSSRCRKPYMHHSRHLHALRRPRGSGGRFLNTKSQNLENSGTNAKKGDGSMQIQSQPKPQQSNSQNSEVVHPENGTMNLSNGLNVSGSEVTSMNYFLSSPVHSLGGMVMPSKWIAAAAAMDNGCCNFKT*

>NF-YA3|AT1G72830

MMHQMLNKKDSATHSTLPYLNTSISWGVVPTDSVANRRGSAESLSLKVDSRPGHIQTTKQISFQDQDSSSTQSTGQSYTEVASSGDDNPSRQISFSAKSGSEITQRKGFASNPKQGSMTGFPNIHFAPAQANFSFHYADPHYGGLLAATYLPQAPTCNPQMVSMIPGRVPLPAELTETDPVFVNAKQYHAIMRRRQQRAKLEAQNKLIRARKPYLHESRHVHALKRPRGSGGRFLNTKKLLQESEQAAAREQEQDKLGQQVNRKTNMSRFEAHMLQNNKDRSSTTSGSDITSVSDGADIFGHTEFQFSGFPTPINRAMLVHGQSNDMHGGGDMHHFSVHI

>NF-YA4|AT2G34720

MTSSVHELSDNNESHAKKERPDSQTRPQVPSGRSSESIDTNSVYSEPMAHGLYPYPDPYYRSVFAQQAYLPHPYPGVQLQLMGMQQPGVPLQCDAVEEPVFVNAKQYHGILRRRQSRAKLEARNRAIKAKKPYMHESRHLHAIRRPRGCGGRFLNAKKENGDHKEEEEATSDENTSEASSSLRSEKLAMATSGPNGRS*

>NF-YA5|AT1G54160

MQVFQRKEDSSWGNSMPTTNSNIQGSESFSLTKDMIMSTTQLPAMKHSGLQLQNQDSTSSQSTEEESGGGEVASFGEYKRYGCSIVNNNLSGYIENLGKPIENYTKSITTSSMVSQDSVFPAPTSGQISWSLQCAETSHFNGFLAPEYASTPTALPHLEMMGLVSSRVPLPHHIQENEPIFVNAKQYHAILRRRKHRAKLEAQNKLIKCRKPYLHESRHLHALKRARGSGGRFLNTKKLQESSNSLCSSQMANGQNFSMSPHGGGSGIGSSSISPSSNSNCINMFQNPQFRFSGYPSTHHASALMSGT*

>NF-YA6|AT3G14020

MQEFHSSKDSLPCPATSWDNSVFTNSNVQGSSSLTDNNTLSLTMEMKQTGFQMQHYDSSSTQSTGGESYSEVASLSEPTNRYGHNIVVTHLSGYKENPENPIGSHSISKVSQDSVVLPIEAASWPLHGNVTPHFNGFLSFPYASQHTVQHPQIRGLVPSRMPLPHNIPENEPIFVNAKQYQAILRRRERRAKLEAQNKLIKVRKPYLHESRHLHALKRVRGSGGRFLNTKKHQESNSSLSPPFLIPPHVFKNSPGKFRQMDISRGGVVSSVSTTSCSDITGNNNDMFQQNPQFRFSGYPSNHHVSVLM*

>NF-YA7|AT1G30500

MTSSIHELSDNIGSHEKQEQRDSHFQPPIPSARNYESIVTSLVYSDPGTTNSMAPGQYPYPDPYYRSIFAPPPQPYTGLMGVQQQGVPLPSDAVEEPVFVNAKQYHGILRRRQSRARLESQNKVIKSRKPYLHESRHLHAIRRPRGCGGRFLNAKKEDEHHEDSSHEEKSNLSAGKSAMAASSGTS*

>NF-YA8|AT1G17590

MDKKVSFTSSVAHSTPPYLSTSISWGLPTKSNGVTESLSLKVVDARPERLINTKNISFQDQDSSSTLSSAQSSNDVTSSGDDNPSRQISFLAHSDVCKGFEETQRKRFAIKSGSSTAGIADIHSSPSKANFSFHYADPHFGGLMPAAYLPQATIWNPQMTRVPLPFDLIENEPVFVNAKQFHAIMRRRQQRAKLEAQNKLIKARKPYLHESRHVHALKRPRGSGGRFLNTKKLQESTDPKQDMPIQQQHATGNMSRFVLYQLQNSNDCDCSTTSRSDITSASDSVNLFGHSEFLISDCPSQTNPTMYVHGQSNDMHGGRNTHHFSVHI*

>NF-YA9|AT3G20910

MGIEDMHSKSDSGGNKVDSEVHGTVSSSINSLNPWHRAAAACNANSSVEAGDKSSKSIALALESNGSKSPSNRDNTVNKESQVTTSPQSAGDYSDKNQESLHHGITQPPPHPQLVGHTVGWASSNPYQDPYYAGVMGAYGHHPLGFVPYGGMPHSRMPLPPEMAQEPVFVNAKQYQAILRRRQARAKAELEKKLIKSRKPYLHESRHQHAMRRPRGTGGRFAKKTNTEASKRKAEEKSNGHVTQSPSSSNSDQGEAWNGDYRTPQGDEMQSSAYKRREEGECSGQQWNSLSSNHPSQARLAIK*

>NF-YA10|AT5G06510

MQTEELLSPPQTPWWNAFGSQPLTTESLSGEASDSFTGVKAVTTEAEQGVVDKQTSTTLFTFSPGGEKSSRDVPKPHVAFAMQSACFEFGFAQPMMYTKHPHVEQYYGVVSAYGSQRSSGRVMIPLKMETEEDGTIYVNSKQYHGIIRRRQSRAKAEKLSRCRKPYMHHSRHLHAMRRPRGSGGRFLNTKTADAAKQSKPSNSQSSEVFHPENETINSSREANESNLSDSAVTSMDYFLSSSAYSPGGMVMPIKWNAAAMDIGCCKLNI*

>NF-YB1|AT2G38880

MADTPSSPAGDGGESGGSVREQDRYLPIANISRIMKKALPPNGKIGKDAKDTVQECVSEFISFITSEASDKCQKEKRKTVNGDDLLWAMATLGFEDYLEPLKIYLARYRELEGDNKGSGKSGDGSNRDAGGGVSGEEMPSW*

>NF-YB2|AT5G47640

MGDSDRDSGGGQNGNNQNGQSSLSPREQDRFLPIANVSRIMKKALPANAKISKDAKETMQECVSEFISFVTGEASDKCQKEKRKTINGDDLLWAMTTLGFEDYVEPLKVYLQRFREIEGERTGLGRPQTGGEVGEHQRDAVGDGGGFYGGGGGMQYHQHHQFLHQQNHMYGATGGGSDSGGGAASGRTRT*

>NF-YB3|AT4G14540

MADSDNDSGGHKDGGNASTREQDRFLPIANVSRIMKKALPANAKISKDAKETVQECVSEFISFITGEASDKCQREKRKTINGDDLLWAMTTLGFEDYVEPLKVYLQKYREVEGEKTTTAGRQGDKEGGGGGGGAGSGSGGAPMYGGGMVTTMGHQFSHHFS*

>NF-YB4|AT1G09030

MTDEDRLLPIANVGRLMKQILPSNAKISKEAKQTVQECATEFISFVTCEASEKCHRENRKTVNGDDIWWALSTLGLDNYADAVGRHLHKYREAERERTEHNKGSNDSGNEKETNTRSDVQNQSTKFIRVVEKGSSSSAR*

>NF-YB5|AT2G47810

MAGNYHSFQNPIPRYQNYNFGSSSSNHQHEHDGLVVVVEDQQQEESMMVKEQDRLLPIANVGRIMKNILPANAKVSKEAKETMQECVSEFISFVTGEASDKCHKEKRKTVNGDDICWAMANLGFDDYAAQLKKYLHRYRVLEGEKPNHHGKGGPKSSPDN*

>NF-YB6|AT5G47670

MERGGFHGYRKLSVNNTTPSPPGLAANFLMAEGSMRPPEFNQPNKTSNGGEEECTVREQDRFMPIANVIRIMRRILPAHAKISDDSKETIQECVSEYISFITGEANERCQREQRKTITAEDVLWAMSKLGFDDYIEPLTLYLHRYRELEGERGVSCSAGSVSMTNGLVVKRPNGTMTEYGAYGPVPGIHMAQYHYRHQNGFVFSGNEPNSKMSGSSSGASGARVEVFPTQQHKY*

>NF-YB7|AT2G13570

MTEESPEEDHGSPGVAETNPGSPSSKTNNNNNNNKEQDRFLPIANVGRIMKKVLPGNGKISKDAKETVQECVSEFISFVTGEASDKCQREKRKTINGDDIIWAITTLGFEDYVAPLKVYLCKYRDTEGEKVNSPKQQQQRQQQQQIQQQNHHNYQFQEQDQNNNNMSCTSYISHHHPSPFLPVDHQPFPNIAFSPKSLQKQFPQQHDNNIDSIHW*

>NF-YB8|AT2G37060

MAESQAKSPGGCGSHESGGDQSPRSLHVREQDRFLPIANISRIMKRGLPANGKIAKDAKEIVQECVSEFISFVTSEASDKCQREKRKTINGDDLLWAMATLGFEDYMEPLKVYLMRYREMEGDTKGSAKGGDPNAKKDGQSSQNGQFSQLAHQGPYGNSQAQQHMMVPMPGTD*

>NF-YB9|AT1G21970

MERGAPFSHYQLPKSISELNLDQHSNNPTPMTSSVVVAGAGDKNNGIVVQQQPPCVAREQDQYMPIANVIRIMRKTLPSHAKISDDAKETIQECVSEYISFVTGEANERCQREQRKTITAEDILWAMSKLGFDNYVDPLTVFINRYREIETDRGSALRGEPPSLRQTYGGNGIGFHGPSHGLPPPGPYGYGMLDQSMVMGGGRYYQNGSSGQDESSVGGGSSSSINGMPAFDHYGQYK*

>NF-YB10|AT3G53340

MAESQTGGGGGGSHESGGDQSPRSLNVREQDRFLPIANISRIMKRGLPLNGKIAKDAKETMQECVSEFISFVTSEASDKCQREKRKTINGDDLLWAMATLGFEDYIDPLKVYLMRYREMEGDTKGSGKGGESSAKRDGQPSQVSQFSQVPQQGSFSQGPYGNSQSLRFGNSIEHLEVLMSSTRTLFITIFRDSTMPVVSENLSDPLSIDMDCEAIYHHFIGLLILSCK*

>NF-YB11|At2g27470

MESEKVVVDELPLAIVRRVVKKKLSECSPDYDVSIHKEALLAFSESARIFIHYLSATANDFCKDARRQTMKADDVFKALEEMDFSEFLEPLKSSLEDFKKKNAGKKAGAAAASYPAGGAALKSSSGTASKPKETKKRKQEEPSTQKGARKSKIDEETKRNDEETENDNTEEENGNDEEDENGNDEEDENDDENTEENGNDEENDDENTEENGNDEENEKEDEENSMEENGNESEESGNEDHSMEENGSGVGEDNENEDGSVSGSGEEVESDEEDE

>NF-YB12|At5g08190

MDPMDIVGKSKEDASLPKATMTKIIKEMLPADVRVARDAQDLLIECCVEFINLISSESNEVCNKEDKRTIAPEHVLKALQVLGFGEYVEEVYAAYEQHKYETMQDSQRSVKMNSGAEMTEEEAAAEQQRMFAEARARMNGGVTVPQPEQLEEPQQQQQTSLQS*

>NF-YB13|At5g23090

MDPMDIVGKSKEDASLPKATMTKIIKEMLPPDVRVARDAQDLLIECCVEFINLVSSESNDVCNKEDKRTIAPEHVLKALQVLGFGEYIEEVYAAYEQHKYETMQDTQRSVKWNPGAQMTEEEAAAEQQRMFAEARARMNGGVSVPQPEHPETDQRSPQS*

>NF-YC1|AT3G48590

MDTNNQQPPPSAAGIPPPPPGTTISAAGGGASYHHLLQQQQQQLQLFWTYQRQEIEQVNDFKNHQLPLARIKKIMKADEDVRMISAEAPILFAKACELFILELTIRSWLHAEENKRRTLQKNDIAAAITRTDIFDFLVDIVPRDEIKDEAAVLGGGMVVAPTASGVPYYYPPMGQPAGPGGMMIGRPAMDPNGVYVQPPSQAWQSVWQTSTGTGDDVSYGSGGSSGQGNLDGQG*

>NF-YC2|AT1G56170

MEQSEEGQQQQQQGVMDYVPPHAYQSGPVNAASHMAFQQAHHFHHHHQQQQQQQLQMFWANQMQEIEHTTDFKNHTLPLARIKKIMKADEDVRMISAEAPVIFAKACEMFILELTLRAWIHTEENKRRTLQKNDIAAAISRTDVFDFLVDIIPRDELKEEGLGVTKGTIPSVVGSPPYYYLQQQGMMQHWPQEQHPDES*

>NF-YC3|AT1G54830

MDQQGQSSAMNYGSNPYQTNAMTTTPTGSDHPAYHQIHQQQQQQLTQQLQSFWETQFKEIEKTTDFKNHSLPLARIKKIMKADEDVRMISAEAPVVFARACEMFILELTLRSWNHTEENKRRTLQKNDIAAAVTRTDIFDFLVDIVPREDLRDEVLGGVGAEAATAAGYPYGYLPPGTAPIGNPGMVMGNPGAYPPNPYMGQPMWQQPGPEQQDPDN*

>NF-YC4|AT5G63470

MDNNNNNNNQQPPPTSVYPPGSAVTTVIPPPPSGSASIVTGGGATYHHLLQQQQQQLQMFWTYQRQEIEQVNDFKNHQLPLARIKKIMKADEDVRMISAEAPILFAKACELFILELTIRSWLHAEENKRRTLQKNDIAAAITRTDIFDFLVDIVPREEIKEEEDAASALGGGGMVAPAASGVPYYYPPMGQPAVPGGMMIGRPAMDPSGVYAQPPSQAWQSVWQNSAGGGDDVSYGSGGSSGHGNLDSQG*

>NF-YC5|AT5G50490

MENNNNNHQQPPKDNEQLKSFWSKGMEGDLNVKNHEFPISRIKRIMKFDPDVSMIAAEAPNLLSKACEMFVMDLTMRSWLHAQESNRLTIRKSDVDAVVSQTVIFDFLRDDVPKDEGEPVVAAADPVDDVADHVAVPDLNNEELPPGTVIGTPVCYGLGIHAPHPQMPGAWTEEDATGANGGNGGN*

>NF-YC6|AT5G50480

MAENNNNNGDNMNNDNHQQPPSYSQLPPMASSNPQLRNYWIEQMETVSDFKNRQLPLARIKKIMKADPDVHMVSAEAPIIFAKACEMFIVDLTMRSWLKAEENKRHTLQKSDISNAVASSFTYDFLLDVVPKDESIATADPGFVAMPHPDGGGVPQYYYPPGVVMGTPMVGSGMYAPSQAWPAAAGDGEDDAEDNGGNGGGN*

>NF-YC7|AT5G50470

MEENNGNNNHYLPQPSSSQLPPPPLYYQSMPLPSYSLPLPYSPQMRNYWIAQMGNATDVKHHAFPLTRIKKIMKSNPEVNMVTAEAPVLISKACEMLILDLTMRSWLHTVEGGRQTLKRSDTLTRSDISAATTRSFKFTFLGDVVPRDPSVVTDDPVLHPDGEVLPPGTVIGYPVFDCNGVYASPPQMQEWPAVPGDGEEAAGEIGGSSGGN*

>NF-YC8|AT5G27910

MENNNGNNQLPPKGNEQLKSFWSKEMEGNLDFKNHDLPITRIKKIMKYDPDVTMIASEAPILLSKACEMFIMDLTMRSWLHAQESKRVTLQKSNVDAAVAQTVIFDFLLDDDIEVKRESVAAAADPVAMPPIDDGELPPGMVIGTPVCCSLGIHQPQPQMQAWPGAWTSVSGEEEEARGKKGGDDGN*

>NF-YC9|AT1G08970

MDQQDHGQSGAMNYGTNPYQTNPMSTTAATVAGGAAQPGQLAFHQIHQQQQQQQLAQQLQAFWENQFKEIEKTTDFKNHSLPLARIKKIMKADEDVRMISAEAPVVFARACEMFILELTLRSWNHTEENKRRTLQKNDIAAAVTRTDIFDFLVDIVPREDLRDEVLGSIPRGTVPEAAAAGYPYGYLPAGTAPIGNPGMVMGNPGGAYPPNPYMGQPMWQQQAPDQPDQEN*

>NF-YC10|AT1G07980

MVSSKKPKEKKARSDVVVNKASGRSKRSSGSRTKKTSNKVNIVKKKPEIYEISESSSSDSVEEAIRGDEAKKSNGVVSKRGNGKSVGIPTKTSKNREEDDGGAEDAKIKFPMNRIRRIMRSDNSAPQIMQDAVFLVNKATEMFIERFSEEAYDSSVKDKKKFIHYKHLSSVVSNDQRYEFLADSVPEKLKAEAALEEWERGMTDAG*

>NF-YC11|AT3G12480

MRKKLDTRFPAARIKKIMQADEDVGKIALAVPVLVSKSLELFLQDLCDRTYEITLERGAKTVSSLHLKHCVERYNVFDFLREVVSKVPDYGHSQGQGHGDVTMDDRSISKRRKPISDEVNDSDEEYKKSKTQEIGSAKTSGRGGRGRGRGRGRGGRAAKAAEREGLNREMEVEAANSGQPPPEDNVKMHASESSPQEDEKKGIDGTAASNEDTKQHLQSPKEGIDFDLNAESLDLNETKLAPATGTTTTTTAATDSEEYSGWPMMDISKMDPAQLASLGKRIDEDEEDYDEEG*

>NF-YC12|AT5G38140

MRRPKSSHVRMEPVAPRSHNTMPMLDQFRSNHPETSKIEGVSSLDTALKVFWNNQREQLGNFAGQTHLPLSRVRKILKSDPEVKKISCDVPALFSKACEYFILEVTLRAWMHTQSCTRETIRRCDIFQAVKNSGTYDFLIDRVPFGPHCVTHQGVQPPAEMILPDMNVPIDMDQIEEENMMEERSVGFDLNCDLQ*

>NF-YC13|AT5G43250

MEEEEGSIRPEFPIGRVKKIMKLDKDINKINSEALHVITYSTELFLHFLAEKSAVVTAEKKRKTVNLDHLRIAVKRHQPTSDFLLDSLPLPAQPVKHTKSVSDKKIPAPPIGTRRIDDFFSKGKAKTDSA*

These are the protein sequences of Arabidopsis NF-Ys, conserved regions only

>NF-YA1|AT5G12840

YVNAKQYEGILRRRKARAKAELERKVIRDRKPYLHESRHKHAMRRARASGGRF

>NF-YA2|AT3G05690

YVNSKQYHGIIRRRQSRAKAAAQKKLSRCRKPYMHHSRHLHALRRPRGSGGRF

>NF-YA3|AT1G72830

FVNAKQYHAIMRRRQQRAKLEAQNKLIRARKPYLHESRHVHALKRPRGSGGRF

>NF-YA4|AT2G34720

FVNAKQYHGILRRRQSRAKLEARNRAIKAKKPYMHESRHLHAIRRPRGCGGRF

>NF-YA5|AT1G54160

FVNAKQYHAILRRRKHRAKLEAQNKLIKCRKPYLHESRHLHALKRARGSGGRF

>NF-YA6|AT3G14020

FVNAKQYQAILRRRERRAKLEAQNKLIKVRKPYLHESRHLHALKRVRGSGGRF

>NF-YA7|AT1G30500

FVNAKQYHGILRRRQSRARLESQNKVIKSRKPYLHESRHLHAIRRPRGCGGRF

>NF-YA8|AT1G17590

FVNAKQFHAIMRRRQQRAKLEAQNKLIKARKPYLHESRHVHALKRPRGSGGRF

>NF-YA9|AT3G20910

FVNAKQYQAILRRRQARAKAELEKKLIKSRKPYLHESRHQHAMRRPRGTGGRF

>NF-YA10|AT5G06510

YVNSKQYHGIIRRRQSRAKAEKLSRCRKPYMHHSRHLHAMRRPRGSGGRF

>NF-YB1|AT2G38880

REQDRYLPIANISRIMKKALPPNGKIGKDAKDTVQECVSEFISFITSEASDKCQKEKRKTVNGDDLLWAMATLGFEDYLEPLKIYLARYRELEGDN

>NF-YB2|AT5G47640

REQDRFLPIANVSRIMKKALPANAKISKDAKETMQECVSEFISFVTGEASDKCQKEKRKTINGDDLLWAMTTLGFEDYVEPLKVYLQRFREIEGER

>NF-YB3|AT4G14540

REQDRFLPIANVSRIMKKALPANAKISKDAKETVQECVSEFISFITGEASDKCQREKRKTINGDDLLWAMTTLGFEDYVEPLKVYLQKYREVEGEK

>NF-YB4|AT1G09030

TDEDRLLPIANVGRLMKQILPSNAKISKEAKQTVQECATEFISFVTCEASEKCHRENRKTVNGDDIWWALSTLGLDNYADAVGRHLHKYREAERER

>NF-YB5|AT2G47810

KEQDRLLPIANVGRIMKNILPANAKVSKEAKETMQECVSEFISFVTGEASDKCHKEKRKTVNGDDICWAMANLGFDDYAAQLKKYLHRYRVLEGEK

>NF-YB6|AT5G47670

REQDRFMPIANVIRIMRRILPAHAKISDDSKETIQECVSEYISFITGEANERCQREQRKTITAEDVLWAMSKLGFDDYIEPLTLYLHRYRELEGER

>NF-YB7|AT2G13570

KEQDRFLPIANVGRIMKKVLPGNGKISKDAKETVQECVSEFISFVTGEASDKCQREKRKTINGDDIIWAITTLGFEDYVAPLKVYLCKYRDTEGEK

>NF-YB8|AT2G37060

REQDRFLPIANISRIMKRGLPANGKIAKDAKEIVQECVSEFISFVTSEASDKCQREKRKTINGDDLLWAMATLGFEDYMEPLKVYLMRYREMEGDT

>NF-YB9|AT1G21970

REQDQYMPIANVIRIMRKTLPSHAKISDDAKETIQECVSEYISFVTGEANERCQREQRKTITAEDILWAMSKLGFDNYVDPLTVFINRYREIETDR

>NF-YB10|AT3G53340

REQDRFLPIANISRIMKRGLPLNGKIAKDAKETMQECVSEFISFVTSEASDKCQREKRKTINGDDLLWAMATLGFEDYIDPLKVYLMRYREMEGDT

>NF-YB11|At2g27470

KVVVDELPLAIVRRVVKKKLPDYDVSIHKEALLAFSESARIFIHYLSATANDFCKDARRQTMKADDVFKALEEMDFSEFLEPLKSSLEDFKKKNAGKK

>NF-YB12|At5g08190

SKEDASLPKATMTKIIKEMLPADVRVARDAQDLLIECCVEFINLISSESNEVCNKEDKRTIAPEHVLKALQVLGFGEYVEEVYAAYEQHKYETMQDSQR

>NF-YB13|At5g23090

SKEDASLPKATMTKIIKEMLPPDVRVARDAQDLLIECCVEFINLVSSESNDVCNKEDKRTIAPEHVLKALQVLGFGEYIEEVYAAYEQHKY

>NF-YC1|AT3G48590

LPLARIKKIMKADEDVRMISAEAPILFAKACELFILELTIRSWLHAEENKRRTLQKNDIAAAITRTDIFDFLVDIVP

>NF-YC2|AT1G56170

LPLARIKKIMKADEDVRMISAEAPVIFAKACEMFILELTLRAWIHTEENKRRTLQKNDIAAAISRTDVFDFLVDIIP

>NF-YC3|AT1G54830

LPLARIKKIMKADEDVRMISAEAPVVFARACEMFILELTLRSWNHTEENKRRTLQKNDIAAAVTRTDIFDFLVDIVP

>NF-YC4|AT5G63470

LPLARIKKIMKADEDVRMISAEAPILFAKACELFILELTIRSWLHAEENKRRTLQKNDIAAAITRTDIFDFLVDIVP

>NF-YC5|AT5G50490

FPISRIKRIMKFDPDVSMIAAEAPNLLSKACEMFVMDLTMRSWLHAQESNRLTIRKSDVDAVVSQTVIFDFLRDDVP

>NF-YC6|AT5G50480

LPLARIKKIMKADPDVHMVSAEAPIIFAKACEMFIVDLTMRSWLKAEENKRHTLQKSDISNAVASSFTYDFLLDVVP

>NF-YC7|AT5G50470

FPLTRIKKIMKSNPEVNMVTAEAPVLISKACEMLILDLTMRSWLHTVEGGRQTLKRSDTLTRSDISAATTRSFKFTFLGDVVP

>NF-YC8|AT5G27910

LPITRIKKIMKYDPDVTMIASEAPILLSKACEMFIMDLTMRSWLHAQESKRVTLQKSNVDAAVAQTVIFDFLLDDDI

>NF-YC9|AT1G08970

LPLARIKKIMKADEDVRMISAEAPVVFARACEMFILELTLRSWNHTEENKRRTLQKNDIAAAVTRTDIFDFLVDIVP

>NF-YC10|AT1G07980

FPMNRIRRIMRSDNSAPQIMQDAVFLVNKATEMFIERFSEEAYDSSVKDKKKFIHYKHLSSVVSNDQRYEFLADSVP

>NF-YC11|AT3G12480

IFQARIKKIMQADEDVGKIALAVPVLVSKSLELFLQDLCDRTYEITLERGAKTVSSLHLKHCVERYNVFDFLREVVS

>NF-YC12|AT5G38140

LPLSRVRKILKSDPEVKIYVNFQKISCDVPALFSKACEYFILEVTLRAWMHTQSCTRETIRRCDIFQAVKNSGTYDFLIDRVP

>NF-YC13|AT5G43250

FPIGRVKKIMKLDKDINKINSEALHVITYSTELFLHFLAEKSAVVTAEKKRKTVNLDHLRIAVKRHQPTSDFLLDSLP

These are the protein sequences of MOUSE NF-Y, conserved regions only

>NF-YAMouse

YVNAKQYHRILKRRQARAKLEAEGKIPKERRKYLHESRHRHAMARKRGEGGRF

>NF-YBMOUSE

REQDIYLPIANVARIMKNAIPQTGKIAKDAKECVQECVSEFISFITSEASERCHQEKRKTINGEDILFAMSTLGFDSYVEPLKLYLQKFREAMKGEK

>NF-YCMouse

PLARIKKIMKLDEDVKMISAEAPVLFAKGAQIFITELTLRAWIRTEDNKRRPLQRNDIAMAITKFDQFDFLIDIVPR
