# Supplementary material for: Identification and characterization of NF-Y gene family in walnut (Juglans regia L.)
Source: BMC Plant Biol. 2018 Oct 23;18:255. doi: 10.1186/s12870-018-1459-2 (PMC6199752; doi:10.1186/s12870-018-1459-2)
Supplement: Supplementary file 5 — The HMM models and domain sequences of NF-YA, NF-YB and NF-YC of Arabidopsis, grape and orange. (DOC 44.0 kb) [file 12870_2018_1459_MOESM5_ESM.doc]

**Additional file 5:** The HMM models and domain sequences of NF-YA, NF-YB and NF-YC of Arabidopsis, grape and orange.

The HMM model of NF-YA, NF-YB and NF-YC domain sequences of Arabidopsis, grape, orange

>NF-YA|Arabidopsis

PVFVNAKQYHAILRRRQSRAKLEAQNKLIKCRKPYLHESRHLHALRRPRGSGGRF

>NF-YA|Grape

PVYVNAKQYHGILRRRQSRAKAELEKKLIKARKPYLHESRHLHALRRARGCGGRF

>NF-YA|Orange

PVYVNAKQYHGILRRRQSRAKAELENKLIKVRKPYLHESRHLHAMRRARGCGGRF

>NF-YB|Arabidopsis

REQDRF-LPIANVSRIMKKALPANAKISKDAKETVQECVSEFISFVTGEASDKCQREKRKTINGDDLLWAMATLGFEDYVEPLKVYLHRYRE

>NF-YB|Grape

REQDRF-LPIANVSRIMKKALPANAKISKDAKETVQECVSEFISFITGEASDKCQKEKRKTINGDDILWAMSTLGFDDYIEPLKRYLHRYRE

>NF-YB|Orange

REQDRF-LPIANVSRIMKKILPANAKISKDAKETVQECVSEFISFITGEASDKCQREKRKTINGDDILWAMATLGFDDYVEPLKIYLHRYRE

>NF-YC|Arabidopsis

QQQLQSFWENQFQEIEQTT--DFKNHQLPLARIKKIMKADEDVRMISAEAPVLFAKACEMFILDLTLRSWLHTEENKRRTLQKNDIAAAVTRTDIFDFLVDIVPRDELK

>NF-YC|Grape

-----MFWSNQNIEQQETN--DFKDHRFPLARIKKIMKADEDVGKISLAAPILFSKALELFLQDLCDRAYAITEDNKRRTLQKNDIAAAIQRTDVFDFLVDIVPKDELK

>NF-YC|Orange

QQQLQMFWSNQKQEIEQTN--DFKNHSLPLARIKKIMKADEDVRMISAEAPFIFAKACELFILELTLRSWEHTEENKRRTLQKNDIAAAISRTDIFDFLVDIVPRDELK

The NF-YA domain sequences of Arabidopsis, grape, orange

>AT1G30500.2/99-155

EEPVFVNAKQYHGILRRRQSRARLESQNKVIKSRKPYLHESRHLHAIRRPRGCGGRF

>AT3G20910.1/165-221

QEPVFVNAKQYQAILRRRQARAKAELEKKLIKSRKPYLHESRHQHAMRRPRGTGGRF

>AT1G54160.1/177-233

NEPIFVNAKQYHAILRRRKHRAKLEAQNKLIKCRKPYLHESRHLHALKRARGSGGRF

>AT1G17590.1/171-227

NEPVFVNAKQFHAIMRRRQQRAKLEAQNKLIKARKPYLHESRHVHALKRPRGSGGRF

>AT3G14020.1/170-226

NEPIFVNAKQYQAILRRRERRAKLEAQNKLIKVRKPYLHESRHLHALKRVRGSGGRF

>AT2G34720.1/97-153

EEPVFVNAKQYHGILRRRQSRAKLEARNRAIKAKKPYMHESRHLHAIRRPRGCGGRF

>AT5G12840.1/171-227

QEPVYVNAKQYEGILRRRKARAKAELERKVIRDRKPYLHESRHKHAMRRARASGGRF

>AT5G06510.1/134-186

-GTIYVNSKQYHGIIRRRQSRAKAEK---LSRCRKPYMHHSRHLHAMRRPRGSGGRF

>orange1.1g026474m/143-199

EEPVFVNAKQYHGILRRRQSRAKAESENKVLKSRKPYLHESRHLHALRRARGCGGRF

>orange1.1g019782m/188-244

DEPIYVNAKQYRAILRRRQYRAKLEAQNKLVKGRKPYLHESRHAHAMNRARGSGGRF

>orange1.1g017825m/177-233

EEPVYVNAKQYHGILRRRQLRAKAELERKLIKVRKPYLHESRHLHAMRRARGCGGRF

>orange1.1g018999m/174-230

QEPVYVNAKQYMGILRRRQARAKAELEKKLIKVRKPYLHESRHQHAMRRARGSGGRF

>orange1.1g021081m/150-206

DGPIYVNAKQYHGIIRRRKSRAKAVLENKITRKRKPYMHHSRHLHAMRRPRGCGGRF

>orange1.1g019764m/149-187

EEPVYVNAKQYRGILRRRQSRAKAELEKKVIKARKVTFH------------------

>orange1.1g019764m/212-234

----------------------------------QPYLHESRHQHAMRRARGCGGRF

>GSVIVT01021622001/113-169

EEPVFVNAKQYHGILRRRQSRAKAESENKVVKSRKPYLHESRHLHALRRARGCGGRF

>GSVIVT01022601001/161-217

DGPIYVNAKQYHGIIRRRQSRAKAELENKLTRARKPYMHESRHLHAMRRPRGCGGRF

>GSVIVT01033313001/175-231

EEPVYVNAKQYHGILRRRQSRAKAELEKKLIKVRKPYLHESRHQHAMRRARGCGGRF

>GSVIVT01015120001/169-225

DGPIFVNAKQYHGILRRRQSRAKMEAQNKLVKARKPYLHESRHLHALNRVRGSGGRF

>GSVIVT01032101001/234-290

EEPVYVNAKQYHGILRRRQSRAKAELEKKAIKVRKPYLHESRHQHAMRRARGCGGRF

>GSVIVT01025252001/135-191

QDPVYVNPKQYHGILRRRQSRAKAELEKKLIKVRKPYLHESRHQHALRRARSSGGRF

>GSVIVT01016790001/180-236

EEVIFINPKQYNGIMRRRKHRAKLEAQTNPVKARKPYLHESRHLHALKRPRGAGGRF

**The NF-YB domain sequences of Arabidopsis, grape, orange**

>AT4G14540.1/20-115

-REQDRFLPIANVSRIMKKALPANAKISKDAKETVQECVSEFISFITGEASDKCQREKRKTINGDDLLWAM

LGFEDYVEPLKVYLQKYREVEGEK-

>AT5G47640.1/26-121

-REQDRFLPIANVSRIMKKALPANAKISKDAKETMQECVSEFISFVTGEASDKCQKEKRKTINGDDLLWAM

TTLGFEDYVEPLKVYLQRFREIEGER-

>AT3G53340.1/27-124

VREQDRFLPIANISRIMKRGLPLNGKIAKDAKETMQECVSEFISFVTSEASDKCQREKRKTINGDDLLWAMATLGFEDYIDPLKVYLMRYREMEGDTK

>AT2G37060.1/28-125

VREQDRFLPIANISRIMKRGLPANGKIAKDAKEIVQECVSEFISFVTSEASDKCQREKRKTINGDDLLWAMATLGFEDYMEPLKVYLMRYREMEGDTK

>AT2G13570.1/35-130

-KEQDRFLPIANVGRIMKKVLPGNGKISKDAKETVQECVSEFISFVTGEASDKCQREKRKTINGDDIIWA

ITTLGFEDYVAPLKVYLCKYRDTEGEK-

>AT2G38880.8/19-112

VREQDRYLPIANISRIMKKALPPNGKIGKDAKDTVQECVSEFISFITSEASDKCQKEKRKTVNGDDLLWAMATLGFEDYLEPLKIYLARYREVF----

>AT5G47670.1/56-152

VREQDRFMPIANVIRIMRRILPAHAKISDDSKETIQECVSEYISFITGEANERCQ

REQRKTITAEDVLWAMSKLGFDDYIEPLTLYLHRYRELEGER-

>AT2G47810.1/49-145

VKEQDRLLPIANVGRIMKNILPANAKVSKEAKETMQECVSEFISFVTGEASDKCH

KEKRKTVNGDDICWAMANLGFDDYAAQLKKYLHRYRVLEGEK-

>AT1G21970.1/58-153

-REQDQYMPIANVIRIMRKTLPSHAKISDDAKETIQECVSEYISFVTGEANERCQ

REQRKTITAEDILWAMSKLGFDNYVDPLTVFINRYREIETDR-

>AT1G09030.1/2-97

-TDEDRLLPIANVGRLMKQILPSNAKISKEAKQTVQECATEFISFVTCEASEKCH

RENRKTVNGDDIWWALSTLGLDNYADAVGRHLHKYREAERER-

>orange1.1g030627m/25-122

VREQDRYLPIANISRIMKKALPANGKIAKDAKETVQECVSEFISFITSEASDKCQ

REKRKTINGDDLLWAMATLGFEDYIDPLKIYLTRYREMEGDTK

>orange1.1g030547m/26-123

VREQDRYLPIANISRIMKKALPANGKIAKDAKDTVQECVSEFISFITSEASDKCQ

KEKRKTINGDDLLWAMATLGFEDYIDPLKAYLMRYREMEGDTK

>orange1.1g036580m/25-120

-REQDRFLPIANVSRIMKKALPANAKISKDAKETVQECVSEFISFVTGEASDKCQREKRKTINGDDLLW

AMTTLGFEEYVEPLKVYLQRFREMEGEK-

>orange1.1g027605m/22-117

-KEQDRFLPIANVSRIMKKSLPANAKISKEAKETVQECVSEFISFITGEASDKCQREKRKTINGDDLLWA

MTTLGFENYVSPLKIYLNKYRETEGEK-

>orange1.1g047516m/38-133

-KEQDRFLPIANVGRIMKKVIPGNGKISKDAKETVQECVSEFISFVTGEASDKCQREKRKTINGDDIIWA

ITTLGFEDYVAPLKLYLSKYREIEGEK-

>orange1.1g026469m/56-152

VREQDRFMPIANVIRIMRKILPQHAKISDDAKETIQECVSEYISFITGEANERCQREQRKTITAEDVLW

AMSKLGFDDYIEPLTVYLHRYREMEGER-

>orange1.1g038014m/33-128

-KEQDRLLPIANVGRIMKQILPPNAKISKEAKETMQECVSEFISFVTGEASDKCHKEKRKTVNGDDICW

ALATLGFDNYADQLKRYLHRYRELEGER-

>orange1.1g044287m/31-126

-KEQERLLPIANVGRIMKQILPANAKISKEAKETMQECVSEFISFITSEASEKCRKERRKTVNGDDICWA

LGTLGFDDYAGPIRRYLHKYRELEGER-

>orange1.1g038325m/54-150

VREQDQYMPIANVIRIMRRILPPHAKISDDAKETVQECVSEYISFITGEANERCHREQRKTITAEDVVWAMGKLGFDNYVEPLSIFLNRFRDSEHER-

>orange1.1g045194m/3-98

-EEQDKLLPIANVGKIMKQILPPRAKISKEAKQTMQECATEFISFVTGEASDKCHKENRKTVNGDDIC

WALSTLGFDNYTEAIVRYLHKYREDERER-

>orange1.1g031569m/11-94

--KEDASLPKATMTKIIKEMLPADVRVARDAQDLLIECCVEFINLVSSESNEVCSREDKRTIAPEHVLKAL

EVLGFGEYIEEVYAA------------

>GSVIVT01014673001/22-117

-REQDRFLPIANVSRIMKKALPANAKISKDAKETVQECVSEFISFITGEASDKCQREKRKTINGDDLLW

AMTTLGFEDYVDPLKIYLHRFREMEGEK

>GSVIVT01016347001/25-118

VREQDRYLPIANISRIMKKALPANGKIAKDAKDTVQECVSEFISFITSEASDKCQKEKRKTINGDDLLWAMATLGFEDYIEPLKVYLQRYREGD---

>GSVIVT01025110001/25-118

VREQDRYLPIANISRIMKKALPANGKIAKDAKETLQECVSEFISFITSEASDKCQKEKRKTINGDDLLWAMATLGFEDYIDPLKVYLHRFREGD---

>GSVIVT01022214001/105-200

-KEQDRFLPIANVSRIMKKSLPANAKISKEAKETVQECVSEFISFITGEASDKCQREKRKTINGDDLLWAMTMLGFENYVGPLKVYLSKYRETEGEK

>GSVIVT01025539001/24-117

VREQDRFLPIANISRIMKKALPANGKIAKDAKEIMQECVSEFISFITSEASDKCQREKRKTINGDDLLWAMATLGFEDYIDPLKLYLAAYREGD---

>GSVIVT01002895001/47-143

VREQDRFMPIANVIRIMRKILPPHAKISDDAKETIQECVSEYISFITGEANERCQREQRKTITAEDVLWAMSKLGFDDYMEPLTMYLHRYRELEGDR

>GSVIVT01017741001/17-112

-KEQDRLLPIANVSRIMKQTLPTNAKISKEAKETMQECVSEFISFVTGEASEKCKKERRKTVNGDDICW

ALAALGFDDYAGPLKRYLQRYRELEGDR

>GSVIVT01031089001/3-98

-DEQDHLLPIANVGRIMKQILPPRAKISKEGKETMQECASEFISFVTGEASDKCHKENRKTVNGDDIC

WALSALGFDDYAEAILRYLHKYREFERER

>GSVIVT01014689001/48-140

----EQYMPIANLTRVMRRVLPA---HAK—ISDDAKETVQECVSEFISFITSEANDRCHHELRKTITAED

VIAAMSKLGFDDYIDPLTLYLHRYRESENER

>GSVIVT01010260001/3-98

-DEQDLLLPIANVGRIMKQIPPP---SAK—ISKEAKETMQECVSEFIKFVTGEASEKCQRENRKTVNGD

DICWALSALGFDDHAEAIVRYLHKYREFERER

>GSVIVT01010959001/1-81

----------------MKQILPPNAKISKEAKETMQECVSEFISFVTGEASDKCHKEKRKTVNGDDICWA

LGTLGFDDYAEPLKRYLHRYRELEGEK

>GSVIVT01014690001/47-142

-REQEHYMPMAHLTRVMRRVLPAHAQISDQAKESIQECVCEFISFITSEANDRSHHELRKTITGEDII

AAMGKLGFDDYIEPLTLYLHRYRQAENER

>GSVIVT01010264001/4-98

--KQDLLLPIANVGRIMKQILPPGAKVSKEAKETVQECVSEFVKFVTGEASAKCRKEDRQTVTV

DDICWALSALGLDDYAGATVRYLHKYREFERER

>GSVIVT01008215001/11-94

--KEDASLPKATMTKIIKEMLPPDVRVARDAQDLLIECCVEFINLISSESNDVCSREEKRTIAP

EHVLKALEVLGFGEYIEEVYAA-----------

**The NF-YC domain sequences of Arabidopsis, grape, orange**

>AT1G08970.1/58-161

QLQAFWENQfkeIEKTTDFK-NHSLPLARIKKIMKADEDVRMISAEAPVVFARACEMFIL

ELTLRSWNHTEENKRRTLQKNDIAAAVTRTD-IFDFLVDIVPREDL-

>AT1G54830.1/48-151

QLQSFWETQfkeIEKTTDFK-NHSLPLARIKKIMKADEDVRMISAEAPVVFARACEMFIL

ELTLRSWNHTEENKRRTLQKNDIAAAVTRTD-IFDFLVDIVPREDL-

>AT5G63470.1/56-160

QLQMFWTYQrqeIEQVNDFK-NHQLPLARIKKIMKADEDVRMISAEAPILFAKACELFIL

ELTIRSWLHAEENKRRTLQKNDIAAAITRTD-IFDFLVDIVPREEIK

>AT3G48590.1/43-147

QLQLFWTYQrqeIEQVNDFK-NHQLPLARIKKIMKADEDVRMISAEAPILFAKACELFIL

ELTIRSWLHAEENKRRTLQKNDIAAAITRTD-IFDFLVDIVPRDEIK

>AT1G56170.1/54-158

QLQMFWANQmqeIEHTTDFK-NHTLPLARIKKIMKADEDVRMISAEAPVIFAKACEMFIL

ELTLRAWIHTEENKRRTLQKNDIAAAISRTD-VFDFLVDIIPRDELK

>AT5G50480.1/35-134

QLRNYWIEQ---METVSDFK-NRQLPLARIKKIMKADPDVHMVSAEAPIIFAKACEMFIV

DLTMRSWLKAEENKRHTLQKSDISNAVASSF-TYDFLLDVVPKDE--

>AT5G27910.1/17-115

QLKSFWSKE---MEGNLDFK-NHDLPITRIKKIMKYDPDVTMIASEAPILLSKACEMFIM

DLTMRSWLHAQESKRVTLQKSNVDAAVAQTV-IFDFLLDDDIEV---

>AT5G50490.1/17-116

QLKSFWSKG---MEGDLNVK-NHEFPISRIKRIMKFDPDVSMIAAEAPNLLSKACEMFVM

DLTMRSWLHAQESNRLTIRKSDVDAVVSQTV-IFDFLRDDVPKDE--

>AT5G38140.1/48-145

-LKVFWNNQ---REQLGNFAgQTHLPLSRVRKILKSDPEVKKISCDVPALFSKACEYFIL

EVTLRAWMHTQSCTRETIRRCDIFQAVKNSG-TYDFLIDRVPF----

>AT5G19490.1/6-86

---------------------QTRFPATRIKKIMQTDEEVGKIAMAVPLLVSKALELFLQ

DLCNHTYDVTLSRGAKTVNAFHLKQCVQATN-VFDFLRDTVAK----

>AT3G12480.1/6-86

---------------------DTRFPAARIKKIMQADEDVGKIALAVPVLVSKSLELFLQ

DLCDRTYEITLERGAKTVSSLHLKHCVERYN-VFDFLREVVSK----

>AT5G43250.1/9-89

---------------------RPEFPIGRVKKIMKLDKDINKINSEALHVITYSTELFLH

FLAEKSAVVTAEKKRKTVNLDHLRIAVKRHQpTSDFLLDSLP-----

>AT1G07980.1/106-188

-------------------A-KIKFPMNRIRRIMRSDNSAPQIMQDAVFLVNKATEMFIE

RFSEEAYDSSVKDKKKFIHYKHLSSVVSNDQ-RYEFLADSVPEK---

>orange1.1g026901m/39-143

QLQMFWSYQrqeIEQ--ANDFKNHQLPLARIKKIMK-ADEDVRMISAEAPILFAKACELF

ILELTIRSWLHAEENKRRTLQKNDIAAAITRTDIFDFLVDIVPRDEIK

>orange1.1g024238m/84-188

QLQMFWSNQmqeIEQ--TADFKNHSLPLARIKKIMK-ADEDVRMISAEAPVIFAKACEMF

ILELTLRSWIHTEENKRRTLQKNDIAAAISRTDVFDFLVDIIPRDELK

>orange1.1g019814m/154-257

QLQSFWANQyqeIEK--VNDFKNHSLPLARIKKIMK-ADEDVRMISAEAPVIFARACEMF

ILELTLRSWNHTEENKRRTLQKNDIAAAITRTDIFDFLVDIVPREDL-

>orange1.1g038850m/6-76

------------EEQ--NTETTRPEFPVGRVKKIIK-LDEDINKVTSEALFIVSRSTELF

LRFLAEKSAEAAIEKKRKTIKLGDMR----------------------

>orange1.1g028727m/105-187

---------------------KVCNFPMGRIKRIFK-TQSSDIGITGEAVFLVNKATDKF

LEQFCEDAYECCAKDRKKSLAYKHLAAVVSEQSKYDFLSDYVPEK---

>GSVIVT01019784001/22-103

------------------HLLPLARIKKIMKrSGEDVKMISGEAPIIFSKACELFIEELT

QRSWKVTLQGKRRTLHKEDVASAVIATDVFDFLVNVVSKS---

>GSVIVT01036760001/1-35

MFWSNQmqeIEQTTDFKNHSLPLARIKKIMK-ADED------------------------

-------------------------------------------

>GSVIVT01036760001/34-69

------------------------------------------------------------

-------EDNKRRTLQKNDIAAAISRTDVFDFLVDIIPRDELK

>GSVIVT01008570001/1-35

MFWSYQrqeIEQVNDFKNHQLPLARIKKIMK-ADED------------------------

-------------------------------------------

>GSVIVT01008570001/35-67

------------------------------------------------------------

----------DRRTLQKNDIAAAITRTDIFDFLVDIVPRDEIK

>GSVIVT01030963001/1-62

--------------------------------------MISADSQILFAKASELFILELT

LRAWFHAEANKRRTLQPCDIGRAIRCYPTLHFLTNIAPDV---

>GSVIVT01036581001/6-86

-----------------DTRFPAARIKKIMQ-ADEDVGKIALAVPVLVSKALELFLQDLC

DRTYDITLQRGAKTMSSLHLKHCVQRHNVFDFLRDIVSK----

>GSVIVT01037394001/6-86

-----------------DTRFPASRIKKIMQ-ADEDVGKIALAVPLLVSKALELFLQDLC

DRTYQITLERGAKTMSSLHLKQCVQRFNVFDFLREIVSK----

>GSVIVT01017901001/5-39

------------------------------------------------------------

-------EDNKRRTLQKNDIAAAITRTDIFDFLVDIVPREDL-

>GSVIVT01025169001/331-410

------------------YNFPMSRIERIVR-SDCDDVRISQEALFLINKASEEFLQQFV

NDAYACSVKDRKNYVSYKHIASAVSKCKRFDFLSDFVPE----
