# Supplementary material for: Identification and characterization of NF-Y gene family in walnut (Juglans regia L.)
Source: BMC Plant Biol. 2018 Oct 23;18:255. doi: 10.1186/s12870-018-1459-2 (PMC6199752; doi:10.1186/s12870-018-1459-2)
Supplement: Supplementary file 6 — Unigene sequences and translated amino acid sequences of 33 walnut NF-Ys. (DOC 116 kb) [file 12870_2018_1459_MOESM6_ESM.doc]

**Additional file 6:** Unigene sequences and translated amino acid sequences of 33 walnut *NF-Ys.*

These are the unigene sequences of 33 Walnut NF-Ys, full length

>JrNF-YA1|Cluster-14922.48043

GGGCATCGGAGTCCATACGGAAGATGACAAGACTCACTCCGTCAGTTAACTCGCGGGGATTATGTGCCTCCCAATATATT

TAAAAAATAAATAAAACGTCGTTTACCCTTGACATAATTTCCATTTATCTTTAAAGAGAGAGCTCTGCAATCAGCGTAGC

TATATTATTTACTAGAACACTGGTCTTGTTCTTGATTATCACAGGCAAATCGATTCTCCCTCTGCTGTCTCCAATACAGA

ATTTATAGGGTTCCAGTCCCCCTCTCAACCGTTACACATATCGGGAGATCATTATTTCAGGAGTGGACTAGGGACCATGC

ATCAAAAGCAGGATGGCACAAACCATCAAAACACAGTCTGCTTTCAACCTTGGTGGCGTGGTATTGGGCATAGTCCCATT

TCGGCAGATTTCTTGGGAGAAACCGCAGCAAGTTTATCTCCATCCAAAAATTCAAATGATAGTTTAGGGTCCAAAACCAG

AAAATTACAAGTTGAGGATGGGCTGGATGAGGGAAATGATGTCAACAAAAAAATAGAAATTACTCTCGTATCACAAGCAG

ATGGAAAATGTGATCAAGAACAGCAGAATGCTGCATCCAGTAAGCCTGAAGCAATGGGTCAATACCTCACTCCACCTACA

GAGCTAGAACTTCTCAGCCACTCAATTGCATGTGCATCGTATCAAGTTTCTAATCCATATTATGGGGGAGTTATGCCTGC

TTATGGATCTCAAGCTTTGGTACATTCTCATTGTCTTGGAGGGCAGCCCACTAGAATGGCCTTGCCCCTTGAAATGGCAG

AGGAACCTGTTTATGTGAATGCCAAGCAATATCATGGAATTTTGAGGCGAAGACAGTCCCGTGCTAAGGCCGAGCTGGAA

AAGAAACTAATAAAGTTTAGGAAGCCGTATCTTCATGAGTCTCGGCACTTGCATGCAATGAGAAGGGCAAGAGGTTCTGG

TGGCCGTTTTCTGAACACAAAGAAGCCTAGTGGTAGTGATGCTAACACTGCACCTGAGAAAGGCGCCATCGGAGCTGTTT

CCTCATATACTTTCAATCCATTGCACTCTGAACAGTCGAACCTCTCCGGGAATATGCATTCATCCTATGATCATATGAAA

GTAACAGGGATTCATGTGCCGGAAACGCACCAACAACCAACATACTCCAATGCCAATGGCAACGGCTGCTATCCACACCA

TCAGGGATTCCAGTTTTCTGCATATCATTCGCTCTCTGGTGATAGGACAGAAGTAGTCGATTGCTCAGAGAAGCAGCACG

AACGACTTATGGTCAATGGGGCTCATAGAGCCCTGATCATCAAATGAACTTTTTACACAACTTCACGTTATTCTTCCTCA

TCGGGTGTGTGTTCTTTTACGAAAAAAGCATCAATCGACAAGGCAAGGCTGGTCTCAGGCAAATCATTCTTGTGTTCCTC

CCATGTACAAAGAAAGTGACTGTTGTAGGGGTTTTGTTTGAAGAGTGAGCAGGTTACATTTTTTTTTTTCCCTTTTGTGT

TTCGTGTTTAGCTTTGACGATTTGCAACTTGGCAATACCTTTCTTTTTTTTTTTTTTTTTTT

>JrNF-YA2|Cluster-14922.79874

GAGAGAGAGAGAGAGAGTGTGTGTTTTTATATATTTGGCACATGATGCAAAGGAGGTTGCATGAAAGCTAAAGCTAATTG

GCCGAGGGCGTGGTATTCCATTTCCATTACACTTGTACCCATTTATTGGCTGTCTGTGTGTCTCTCTCTTGTACACTCGC

TTGAGATTGTACAGACATGGCTCGTACCAAATGGGGTTTCTGTGTTTGAGCGGGCGATTCAAAGCTGCTGCTTTCTACTA

AGAGAGATTTGCAGGGAGTAGAGTGAACCTTCCAAATACAGTTAACCTCAGAAACCTTGCTCGGACCATTTTGGAGCGTT

AAATGGCTGCTGAAACGTTCTATTTCAAACAACGTCAAGGGACTGTCCACAATCCTATAGGGCAGCTGTCAGCGCCTTGG

TGGAGTGCCATCACTTCTCCATCGGTTTATGGGGAAGCCTGTGTCCAATTGAAGCCTTTGTCCATGGAACACCCCAGCGG

TGGAGACCAACTGATTACCAACAACCAAGCAGCAAGGGGTACCGAAGCTGCGGCTAGAGCGAATACAACTCGGTTCACTC

TCTTTCCCGATAATTGCAGAAGTTCAGCGGATGGGCAAAAATCTCAGGTAGCAATTTCTCTCCAATCAGCTCTGCCAGAA

TATCGAGGTTGCTTAGAGCTAGGATTTGGGCAGCCGATGATCTATGCAAAGTATCCTTATGGGGACCAATGTTATGGAGT

CTTCCCAACTTGTGGACCTCAAATTCCGGGCCGTGTTATGCTGCCATTGAACATGACAACTGATGATGGACCCATATATG

TTAATGCCAAGCAGTACCATGGGATTATCAGGCGCCGGCAGTCCCGTGCAAAGGCAGTTCTGAAGAATAAGTTGACTAGA

GTTCGTAAGCCATATATGCATGAATCACGCCATCTCCATGCTATGCGCCGACCAAGGGGATGTGGTGGCCGTTTCTTGAA

CACAAATATTTTGAACAAACGAAAGAATGGAACTGAACTGAACAAAAACGGTGATGGACAAATTTCTCAGGCCACTGGTT

CTCAGAGTTCTGAAGTACTGCAGTCTGATAGTGGTACCTTAAACACTTCAAAGGATGCTAATGGCAGTGGTTCAAATATT

TCTGTGTCAGAGGTGACTAGTATGTTCTCTAGGGGAGATCTTGATTACTTTCCAATCAACCATCTCATGCCTTCTGTCCA

CTCTCTATCAGGCAAGATAGATGGTGGGCATGGCATTATTATGCCCGGTAAGTGGGTTGCAGCAGTAGACAACTGCTGCA

GCCTCAAAGTCTGATAGCAAGGGATTGGGTGGTGTTGGTGCTAAATGCGCCAATCTCCATCCTTGCTGCAACCAGATGGA

GAAGTTTCATTGCAATGACTTGTTCTTGCAACAGGCTTTTGTTGAGGGGGTTGTCCCCACCATGTGGTTGCTAAAAATGG

CAATTCATTCTTGGCTCATGTGCATCGAGGCTGTCTTCTGCTATTCCTTGCAGCCTTTCTGGCTTAAGGTGGTTAGAGAT

GCCTGATGTACTTTTATGTACTGTAGATTAATGGAGTAGTTGATAAATAAGTCTTGTTAGCTAATTAAATAAGAGCATCA

ATGTCTGGATATGAGTTTGTGAATTAGAGATATTGAAGTGTGATGCTGTTAAAGTTTGATTATCTAGTATGTCTCTGTCT

GACTTTTGGAGTGATGTATCCTGTAACTTCGCGTCTGTTTTGTTATCTTATACTTTGGAAGCATCAAGCTTTACTCAAGA

ATTTGGTGTTCCTGTCATCTGTATTTGAAGCATAATGCCTTATTCCTATTCTTGATTTGCAGTTTGAGAAACACTTTTTC

TAAAGATGTAAAGGATTCATTATTAGCTAGTCGCGTGTGCTTTGCTTGGGTGATAGAATGTGTTTGGAATAACTCAGTGA

AGGTGTTCGATAATATGAGTAAGTTCTCTGATTCTTTGCTGTGAGGTACAAATTACAATACACGAT

>JrNF-YA3|Cluster-14922.46846

GCTCTCTATAACAGAAACAACCTGGTATTGGAAATTACAACAATTATTATAGATATAAACACTTGGTGAGATATAAAGAT

GAGAATAAATAAGACAACAAAAATTGAAGCCAGAGCATCAACCAACTCAAAAATAAGCTGATCCTACTGCTACATAGTAT

CCATAGTTTTTTTTTTTATTAGTAAACATAATTTTATTGATCACAAGAATAGGCAAAAACCCAGGTATACGGGATATATA

CAAGAGCGTAATGTCGCCTATATATGCGACACACCATTGTTAAGCATTTTCCTCCATCTCTTTCCAAACCATGCCACACA

TGGGAATACAAAACATGTCAATGTTTCGCTTGACACACTTGTGCAAACAATTTAGGAACAGCAAAAGTGATGTATTAGCA

ACCATATCGATGTTCTCTTATTAACACATGCAAATGAAACAGTTTGGTAGAGATTACATCGTGGTATGCCAAAAGCTTTT

TGCAAGACATGTAGGAAACATACAGAAAGTAAAGTAACAAAGCCAAGGATGAATTGCCAGACCAGTAACGTGGTCTTTTG

TATTCGTCTTACCGGACGACTGAAGCACAGTGCTGGGTGCCACCAGACACAAGGCCTCCATTGCATTGCATGGATCCACC

CATGAGGGGAGAGATGCCTGATAATCTAGGATCCCGCAGCTGAAACGTATTGTTATCATTGATATTGGAGATAAGTGTTA

TGTTGGAGCAGGTGGTATCTTGACCAGCCGCAAATCGATCACTATCAAACTCAGACATAGTTTTACTTGAAAGCAAGTCA

GTGGCGGCCGGATCAGGATTATGAAGCTTTCTTGTGCTGACAAAGCGTCCACCGGATCCCCTAACCCTATTTAACGCATG

ACGATGCCGTGACTCATGAAGATATGGCTTTCGAGTTTTGACAACTTTTGTTTTAGCCTCAAGCCTTGCACGTGACTGCC

TCCTTCTAAGAATTCCATGGTACTGTTTCGGATTGACATAAATGGGTCCATCATCTGAAAAATCAAGTGGCAGTGGGACT

CGTGCAGACACTCTCCCCACCATCTCGGGCTGAATAATAGCTGGTAGTCTGTAAGTAGTCAACAAGCATGGCTCGGCATA

GGCATGTGACACTCGAGACTGAAATGAGAGAAGAATAAGTTTACAAGAGTATCTAACAAACAGGATTCCATAATCTACAT

ATCAACCTACCATTAAATAGTTGTGAGCAACTTGTGAAGGATTGCAAATAATATATGGATTATTCAATAAGAAAGGCTTC

TTTTGACCTTCCACATCCCTGACACAGCCTTCATCTCCATCAGATTCTGATGAAATGCATTGATCTTGAGAATTGCTCCC

TCCCGCAGCACCTGCTTTGGGGTGAAATTGACCAGTCAACTGATTTGTAGTTGATTCCTGGATCGGTAGTTGAAGACCTA

AATGCTTTGCATCATGACAAAGTTGTGGAGGAGATCCTGCTTTCAAGCATGCATTTTCAGATAAAGCTGGTGGAATTCGA

TGTACATCGGAATTCCACCACAATGGACAATTAGCAGTAAAACGGGGCATTGAATGAACAGGAATTCCGATCGTTTCTTG

GCAAGTCCAGAATTCGTATGGCCTCAAAGTTGTCAATACTTCGGACTTTTATAAAAAAGCCACGGGACCTTCAACAAGTA

GGTTCCTTTCCACTCTTTTAGGGATTAGCTTTGGGATCCACGAAGACATGAAGAAGCAAGGAATAGTACTGTTGGGATGA

ACTTGGACATGTCTCCCATCTTGAGTCTTGGAATCAACTCATACACACAAATA

>JrNF-YA4|Cluster-14922.46847

GCTCTCTATAACAGAAACAACCTGGTATTGGAAATTACAACAATTATTATAGATATAAACACTTGGTGAGATATAAAGAT

GAGAATAAATAAGACAACAAAAATTGAAGCCAGAGCATCAACCAACTCAAAAATAAGCTGATCCTACTGCTACATAGTAT

CCATAGTTTTTTTTTTTATTAGTAAACATAATTTTATTGATCACAAGAATAGGCAAAAACCCAGGTATACGGGATATATA

CAAGAGCGTAATGTCGCCTATATATGCGACACACCATTGTTAAGCATTTTCCTCCATCTCTTTCCAAACCATGCCACACA

TGGGAATACAAAACATGTCAATGTTTCGCTTGACACACTTGTGCAAACAATTTAGGAACAGCAAAAGTGATGTATTAGCA

ACCATATCGATGTTCTCTTATTAACACATGCAAATGAAACAGTTTGGTAGAGATTACATCGTGGTATGCCAAAAGCTTTT

TGCAAGACATGTAGGAAACATACAGAAAGTAAAGTAACAAAGCCAAGGATGAATTGCCAGACCAGTAACGTGGTCTTTTG

TATTCGTCTTACCGGACGACTGAAGCACAGTGCTGGGTGCCACCAGACACAAGGCCTCCATTGCATTGCATGGATCCACC

CATGAGGGGAGAGATGCCTGATAATCTAGGATCCCGCAGCTGAAACGTATTGTTATCATTGATATTGGAGATAAGTGTTA

TGTTGGAGCAGGTGGTATCTTGACCAGCCGCAAATCGATCACTATCAAACTCAGACATAGTTTTACTTGAAAGCAAGTCA

GTGGCGGCCGGATCAGGATTATGAAGCTTTCTTGTGCTGACAAAGCGTCCACCGGATCCCCTAACCCTATTTAACGCATG

ACGATGCCGTGACTCATGAAGATATGGCTTTCGAGTTTTGACAACTTTTGTTTTAGCCTCAAGCCTTGCACGTGACTGCC

TCCTTCTAAGAATTCCATGGTACTGTTTCGGATTGACATAAATGGGTCCATCATCTGAAAAATCAAGTGGCAGTGGGACT

CGTGCAGACACTCTCCCCACCATCTCGGGCTGAATAATAGCTGGTAGTCTGTAAGTAGTCAACAAGCATGGCTCGGCATA

GGCATGTGACACTCGAGACATTAAATAGTTGTGAGCAACTTGTGAAGGATTGCAAATAATATATGGATTATTCAATAAGA

AAGGCTTCTTTTGACCTTCCACATCCCTGACACAGCCTTCATCTCCATCAGATTCTGATGAAATGCATTGATCTTGAGAA

TTGCTCCCTCCCGCAGCACCTGCTTTGGGGTGAAATTGACCAGTCAACTGATTTGTAGTTGATTCCTGGATCGGTAGTTG

AAGACCTAAATGCTTTGCATCATGACAAAGTTGTGGAGGAGATCCTGCTTTCAAGCATGCATTTTCAGATAAAGCTGGTG

GAATTCGATGTACATCGGAATTCCACCACAATGGACAATTAGCAGTAAAACGGGGCATTGAATGAACAGGAATTCCGATC

GTTTCTTGGCAAGTCCAGAATTCGTATGGCCTCAAAGTTGTCAATACTTCGGACTTTTATAAAAAAGCCACGGGACCTTC

AACAAGTAGGTTCCTTTCCACTCTTTTAGGGATTAGCAAGACTAGAACTATTTTCCCATCATCATCATCGCTATGATTTC

AAACTCCACATGCACCCAATAAACAGCAAAAACAAGAAATCAACTAAGATTCCAGGCCAATAAATGAGCCCCTACAAGAG

GAAAAGTAAATGACAGAGAGAGCGAGAGAGAGAGAGA

>JrNF-YA5|Cluster-14922.50230

GTTTTTGTTTTTGTTTTTTTTTTTGATGTACTCAAATCTGGATGTTCAAATTGTAAGTATTAATACCGAGGGAAAGCAGA

GCTGCCTGAAAGTGACATGTAATTTGATTATCTATATTTTTTGTATTTTAGAACTGGTATTATAGTTTAGAAATTCATCA

CATAATACTTAAAAGAAAGCAAAGGTATTCGAGATGTTTCTTATAGTTATTTCTAATTTATGGCATGGAGGCCTCCTGCT

TGAAAATGCAATGGTAATACGGTGATCTATGATTAGAAATTGTGTATTGGACTTGTGGTCATAGGGCATGCTCGTGTACT

GTACTAGACTATACGTTTACTCGTAATAATGCAATTAACAAAATCGTTTCACTTCAGTAGTTAAGTCAAATTTTTATTGT

CACTTCTTATTTCCTATTGGAATTGAGTTTCCTTAGTTTTAATGATTCAATTCGTAGCGTATGCATATCTAATTCGTATA

TGTGCAAGCATGCGGGTGCGCACGCACACACACGCACACAAGTGGTTGCAACTGAAAACAAGTGTAGGTTTTCCTCTTTT

ACATTATTAAAAACCCTAGTCTATTTCTCATAATTCTTAAATTCTCCAAGCAGTAGTTTGAGCCAATGCAGAACTTGTGT

AAGAAAGAATCTGTTATAAGTTTTCCCTATTCTGCATCCCCATACTTTCTTGGTTGCCCATCATGGGAGAATTCTGCTGA

GTCACATGTTGAGCAATCATCTACATCCAGAATTCTAAGCATGAAGATGGGAGTTTCACCGCAACATTTTCATAACACCA

AGCAATTGAATTTTCAATTCCAAGATCAGGATTCGTCCTCAACTCAATCAACCTGTGAATCTTATCCTGAAGTGGTTAGT

ATGGGAGAAAGAAACCCGTGTGTACAAGGCATAGTTTCACCACAATCAGGATTTATTGAAACTAAGGGGAAGCCTGTTGG

GGGTATCATCAAATCTGGCTCACCAATTGGTACTCGGGATTTTGTCTTCTCACCTTCACAGCTTGATTATAGCCAATCAA

TTGCTCGCATTCCATTCCACTATGCAGATCCGTATTTTGGTGGTTTACTGGCTTCTGCTTATGGACAACAGTCTATTATT

CATCCTTCCCAAATGATAGGGGTGGCACCTACACGAGTGCCCCTACCTCTTGATCTTATAGATGATGAACCCATTTATGT

CAATGCAAAACAATACCATGCAATTCTCAGACGGAGACAGTATCGAGCCAGGCTTGAAGCTCAGAACAAAGTCATCAAAG

ATCGGAAGCCATATCTTCATGAATCTCGTCATCTTCATGCATTGAAGAGGGCTAGGGGATCTGGTGGACGCTTTGTTAAC

ACAAAGAAGCTCCAAGAATCCAAGCAAACTCCAATGAGCAATGGGCTAGATGTCTCAGGCTTTGCTCAGCCACTTTTGAC

TAGAGATATGGCAGAATCTGAAGTTCATCTGCCAGAGAACTATAGAGACAGAGCACCCACCACCTCTTGCTCTGATGTGA

CTAGTGCCTCCAACAGTGACGACATCTTCCAGCAGCCAGAATTCAGGTTCTCTGGCTACCCTTCTTCATTTGGCAGGACT

ATGCAAGTTCACTTGGTTGATATGCATGATGGTGGTGGTGGTGGTAAGCATCACCTCCGATGAGAGATGTTGCCAACCAC

ACTCATTCAGCAGTTTGTGTTCCATTTTTGGAGAAGGTAGCTGCTGGAGTTCTGAAGGGAAGTCATCCTTGGCTCTTTTG

CTATTTACAATTTCACTTCTTTGTCACCGTAGCTTGTCAAAAACCAAGTGTAGACTGTTACTGGCTATACACTCTTTTGG

CATTTGGGCATAAACTAGCATCTCAAGACTACTACATGTCAAGTCGGTGATCATTGACAATATCGTAATTGCTCCTTATG

TTCATGATATGCTTTTGTCTGTGGTAACTCTCTGCCGACTTTTTCTAGTCTCTGATGGCAAAAATTTCTGTTGAAAGCGG

CATAAAAAATTAAAAAACTCTCAT

>JrNF-YA6|Cluster-14922.72325

TGTTGGCTTCGAAAGACAAAATATAGTTGGATCAACAATTTCATCCACAAGTCAATCAAAATCAATGTTTGAAAAGCTTA

ATGTTCCGCACAATTCAAATCAAATCCTATGAAATGATCAACATAAACAACACAAAGATAGATCATCAAGCCTCCTATCT

AATTAACAAGACGGTTTGTACATTAACCCAAGAGTTTAAAAGGCAAACTTTGAGTCGGCTAATTAATCTCTACCTTTGTA

CAAAGAGCAAAAATGGAAGAAGAGGCTGAAATAAAGAACTGGAAACCTTTTAAGAAGCCGTTCAACTTCTCCACTCTTCA

TCCATGTCACATGCACAATATTAAAGCCAAGAATGATTTGCCGCCTGGCATATAAATTGCCAAGAATGCTGTCTGTAACA

GATTCACAGGGACTGATCCCGTTCCATCATGGTGGGGTCCAATCCACAATCCTGGCACCCAACTCCCTCCCCACAGGAGT

TCATTGTATTGCAAGACGCCTCTGTGAGGCCTGGTTAGAAGAGATGCTTCCCCATTGCTGGCCTGAACAGTCTCCTTCCT

CGCCTCTTCCGCCCATATGCAGATATGATGAGACAGGTAAGACCTCATGATTTTGGTAGCGGTTGTCACCATTTACATAA

TTCTGAGCTTCAGAAGCATCTTGCCTCCCTTCCTGTTGCCCATGAGAGGAATTCCATATTTCAGCAGAGTCAGTGAGCAA

GGGTTCAGAACCAGACGAACTTGCAGACTGTGATGAGAGAGCTGGCCCAGAAGCAATGCCCTTTTCTTTGGCTTCATGGT

TCGCAGCATCAACATTACTTTTCTTTGCAAAACGTCCTCCACTTGCCCTGGCCCTTCTCATAGCATGCTGGTGTCGAGAC

TCATGCAGATAAGGCTTTCTAACTTTTATCAACTTCTTTTCAAGCTCGGCCTTAGCACGTGCCTGTCTCCGCCTCAGAAT

ACCTTGGTATTGTTTGGCATTCACATAAACAGGCTCTTGTGCCATCTCGAGAGGTAAAGGCATTCTGGCATGAGGCATTC

CAAGAAAAGGAGGATAGCCCTGACCATGACAAATACATGTCAGACCATTCCATATTCTAGCAAATTAGTCATGAGAAATA

TTTGTTTCATGTAAAACAAGCGTCTTATTTCTGTGAAGTTTATAACTCCACCAAAAGGAAAACTACATCAATTGACTAAA

AGCAAACAACCTTCTAGCACATAAAATTATTTCTATTATCATTATCACTGTTGTTACAGCATAAGCATAATGAAAAATTG

CTCCTCAATAAAAATGAAACAAAAATCATGGAACACCAGAAACGAGAAATACTTTGCATATACTTTCTCGTTGCTGCTAA

GCCAACCATAGATTGGCAGACAAATAAAGTGACAAAGAAAACCACCCATGAAGACAGATCTAGCGATCTAGCGCTTCGGG

CAGACTGAGTATAGTTAAAAAGGAAAGCCTTCAATTTGTGACCATGTCTGCAGGTATGAATAAAAAATGACCATTTCCTA

CCCAATCGGATTAATCCCAGTCCCCACTCCCCCAATATATTTATACAAGAAACTTTTTTTTAAAAAAAAAAAAAAA

>JrNF-YA7|Cluster-14922.32667

GAGAGAGAGAGAGAGAGTGTGTGTTTTTATATATTTGGCACATGATGCAAAGGAGGTTGCATGAAAGCTAAAGCTAATTG

GCCGAGGGCGTGGTATTCCATTTCCATTACACTTGTACCCATTTATTGGCTGTCTGTGTGTCTCTCTCTTGTACACTCGC

TTGAGATTGTACAGACATGGCTCGTACCAAATGGGGTTTCTGTGTTTGAGCGGGCGATTCAAAGCTGCTGCTTTCTACTA

AGAGAGATTTGCAGGGAGTAGAGTGAACCTTCCAAATACAGTTAACCTCAGAAACCTTGCTCGGACCATTTTGGAGCGTT

AAATGGCTGCTGAAACGTTCTATTTCAAACAACGTCAAGGGACTGTCCACAATCCTATAGGGCAGCTGTCAGCGCCTTGG

TGGAGTGCCATCACTTCTCCATCGGTTTATGGGGAAGCCTGTGTCCAATTGAAGCCTTTGTCCATGGAACACCCCAGCGG

TGGAGACCAACTGATTACCAACAACCAAGCAGCAAGGGGTACCGAAGCTGCGGCTAGAGCGAATACAACTCGGTTCACTC

TCTTTCCCGATAATTGCAGAAGTTCAGCGGATGGGCAAAAATCTCAGGTAGCAATTTCTCTCCAATCAGCTCTGCCAGAA

TATCGAGGTTGCTTAGAGCTAGGATTTGGGCAGCCGATGATCTATGCAAAGTATCCTTATGGGGACCAATGTTATGGAGT

CTTCCCAAGTTGTGGACCTCAGATTTCGGGCCGTGTTATGCTGCCTTTGAACATGTCAACTGATGATGGACCTATATATG

TTAATGCTAAGCAGTACCATGGGATTATCAGGCGCCGGCAGTCCCGTGCAAAGGCAGTACTGGAGAATAAGTTGACAAGA

GTTCGTAAGCCATATATGCATGAATCACGCCATCTCCATGCTATGCGCCGACCAAGGGGATGTGGTGGTCGTTTCTTGAA

CACAAAGAATTCGAACAAAGGAATGAGTGGAACTAAAGTGAATAAAGCTGGCGATGGACTAATTTCTCAGCCCACTGGTT

CTGCGAGTTCTGAAGTCCTTCAGTCTGATAGCGGTGGTGCCTTAAACTCATCAAGGGATGCAAACAGAAGTGTTTCAAAT

ATTTCTGTGTCTGAGGTGACTAGTATGTTCTCTAGGAGAGATCTCGATCACTTTCCAATCAATCATCTCATTCCTTCTGT

CCACTCTCTGTCGGACATGATAAGTGGTGTACGTGGCATTGTTATGCCCAGTAAGTGGGTTGCAGCACCGAACTGCTGCA

ACCTCAAAGTCTGACAGCAAGTGATCAAGTGGAGTTGGTGCTAATTGCACCAATCTCCATCCTTGCTGCAACCAGATGGA

GAAGTTTCATTGCAATGACTTGTTCTTGCAACAGGCTTTTGTTGAGGGGGTTGTCCCCACCATGTGGTTGCTAAAAATGG

CAATTCATTCTTGGCTCATGTGCATCGAGGCTGTCTTCTGCTATTCCTTGCAGCCTTTCTGGCTTAAGGTGGTTAGAGAT

CCTTAATGTAAATGTATTGTAGGTTAAATGGAGTAGGTGATAAATAAGTCTGTTTCACTAATTGAATCAGAGCATCAAAG

TCTGGATAGGTATTGAAGTGTGATGTTTAAGTTTAATATCTAGTACGTCTATGTCTGACTTTTGGTTTGATGTATTGTAG

AACTGTGTGATTGTGTTGTTATCTTATGCCACCTTGCTTTAGTCAAGAACATGGTATTCGTGTTGTCATCCATATTTGAA

GCACAATAATGCATTATGCTATTCTCGATCTGTAGTTTTGAGAAACGCTATCTAAAGATGCAAAGAATTAATT

>JrNF-YA8|Cluster-14922.32809

CCAGGCTTTCTTAATTTCCTCCCAACCCCTTATCTCAACCGACCTATGGGATGAAGGGCTGCTGTTATAGGCAACCAGAC

CTCCATTCAATCATAGATTGATAGGATGCACCTCCTATCTGTGCTTTGCATCTTTATTGTCATCTAACACTCGTCAAATT

GCAATAAAAGCAGAGAGAGAGAGATAAGCTCATAACATTGTCAACATAACACCATCAGGCTAGCAAACTCTTGTCATAAA

CAAGCATCACACTTCTGAAACCTCCATACCTAAATTGTTGAAATTTTACACATACATAACCATTTCCTACTTGATAAGTT

AGGAACTTTGAGCATTTTACACTAGGAGGGAAAGGAAGAAAAATGTATGAACTTCAGACCATAATGGCAGCGAGTCATAT

TTGTCTCCCAATTCTCACCTAGAATTAAGACAAGTAAACTTGTTTTTTTTAAGCCAAGAATGAATTGCCTGAGAAACACG

AGGGTTTCTCCCAAAGCTGGATGCGGCGAACACCTTCTGGTTCACCTTCAGCGAATGTCATTTAATGGGGAGTGCCCCAT

GTGCAACCCCGTTCACCTGCATGCTCTCTCTCTGCTGACCCAAGCAGTCTACCTCCTTACCATCACTAAACAGTGAATGG

TAAGTTGATGACAGGCCATTGCTACTGCTGTTACCTTTGGAAAAACAGTGCGCTCCATGTGTGTTGTGAACTATGGGCAG

TGATCCTTTGTGCTGATTAAGCGAGGAACCTGAATTCCCATTGCCATTGTTGAAAAAGCATTTAGAGCCAGATGAGATGG

TTGGCCCCATAGAAGGTTTAGCACCCATATTCATGCCTTCTTCTGAAGGGGGATCAGAAAGATTATCAAGCTTTTTTGTA

TTAAGAAAGCGCCCCCCACAGCCTCTTGCCCTCCTCATAGCATGTAGGTGTCGAGACTCGTGCAGATATGGCTTCCGAAC

TTTTATCACTTTCTTCTCAAGCTCAGCCTTAGCACGTGATTGTCTTCGTCTCAGAATACCATGATATTGCTTTGCATTTA

CATAGACAGGTTCCTCTTCCATTTCAAGAGGTAAAGGCATTCTAGCATGATGCATTCCATATAATTGAGGTATCATAGCT

TGGGGCCCATAAGAAGCAAACATTCCACCATACTGTTGATCTGAATAAGGATATGATGTCAATACAATTGAGTGACCAAC

AAGCTCCATTTGGGAGTTCGGATCAAGGTGTTCAACCATTTTAGGGGTTAAGGATGGAACACGGTTATTATCTCGGTGCT

CTTTCCCAATATTCCCATCCCGTGTTTCTTTGTTGAATTTGGCTCCATCATCTAGCGGGCCATTCACTTTCAATTGCATG

GCTCCATTCCCAACAGAAACATTTAAGTGTTCCATTGAAGTTGATTTTGAAGCATTGTCACCTAAGGACACATTGCTCCC

CACGCCACGCCACCAAGGTTGAGAATATGTGGCTGACTGCAACACAGTCTGCCCACCATGTTCTAATCGTCCATCTTCAT

TTCCAGGCTTTGCTGGCATCATTCACAACTTCCTCGATGCAGCTGATCGGAACTCAAGACGCTTTGGTCTTTCACAAGTC

GAAGCTCATCGGGACTGGAGTCTCTCTCTCTCTCTCTCTCT

>JrNF-YA9|Cluster-14922.60069

CATGAATCATATAAGATCACCTCAGCATCCGCACATAATCTAAAATGACTAGTAATTGACACGGTTATTGGGACACGGAT

TGGGTCCAATTCCGTACAAGTCATTACCACTCGTACCAAAGATGATAGGCACCAGATTGGTTGGAAAACTGTCTTGAACC

CTGAAAACACAACTTGAGCCAAGTTTAATTTTAGGGAGGGCCAAACAGAATCCAGAAATGAACCATATTCAGAAACTTCA

AATTCTCTTTGATAATATCCTATTCGTGCACAGAATTGGTAGCAACAACATAAGTGATGTATTAGCAAAGTTGAGTTAAA

AGCACAATCTTCTTACAAAACCATGTCAATATCCTTCATGACACCATGCAAACGAAATTAGTAAAGATCTTAGTACGATG

TGGCAAATGTAGCGCATTTCGGAAGACAAGCAGGAAACATAGAAAGTAAAGTGACGGAGCCAAGGATGAATTGCCTGACC

GATGATACGGATCATCGATCTTGTTTATTTCCCATGTCTCACTGGACGACTGAAGCACTGTGCTGGGTGCCTCCAGACAC

AAGGCCTCCATTGCAATTACACTGCATGGGTCTAGAGAACCTCCTAAAGCTACCGTTACCATTGTTGCTATTGGAGACAT

GCTTGATGTCAGAGCAGGTGGTAAGGGAAGCAACATCTTGAAACTGATGACTATGAAACTCAGACATTTTTTTACTTGGA

TGCAAGTCAACACAATCTGAGGAACAGTGAGTGTTGCTAGTGGCGGACGAATCAGGATTTTGGAGCTTTCTTACGCTAAC

GAAGCGTCCACCAGATCCCCTTACCCTATTTAGGGCATGACGATGCCGAGACTCATGAAGATAAGGCTTTCTAGTTTTGA

CAACTTTGTTTTGAGCCTCAAGCTTTGCACGTGATTGTCTCCTTCTGAGGATTCCATGGTACTGTTTTGGATTGACAAAA

ATGGGTCCATCGTCCATGAGATCAAGTGGCAGTGGAACTCGTGCGGGTGCTATCCCCACCATCTGGGGTTGAATAATAGT

TTGTTGTCCATAATTAGTCAATAATCCACTAAAGCATGGCTCAGCATAAGGATATGGAACTCGAGCCTGAAATGAGAGTA

GAGACATGAGTTTAAAGGCATATCTAACAAACAGGATTTCAAAAGATCAACCTACCATTGAATGGTGATGAGAGATTTGT

GAAGGATTGATTATGATATCTGGATTATTCAACAAGTAAACCGGCTTCATTTGACCTTCCACGATGCGCTTGACACAACT

TCCATCTCCATCAGATTCAGATGAAACACATTGATCTTGACAATTGCTCCCTCCCATAATATCCACTTTATGGCGAGATT

GACCAGTCAACTGAATTGTGGACGATTCCTGGTCTGCTAGTTGGAGACCTAAATGCTTTGCATCGTGACAAAGTCGTGGT

GCAGAGCCCGCTTTCATGCTTGCATTTTTGGATAAAGATGACAGAAATTTCTGTTCATCTGGATTCCACCACGAAGGGAA

AGGGACAGCAGAATGGGTCATTGAATGAACAAAATTACACTTCAATTGTTTTCTAGGTAATTTTAGAATATGTGTAGGGT

CAAAGTTATCTGTCTTCTGAATTTGATGCAAAATCTACAAGATCCTCAACAGCAAGCAGGTTCCTTTCCACTCATTTAAC

ATCAGCTACTCTTTGGGATCCACTAAGACATGAAGAAGAAGCGAGGAATAGTGCTGCTGGTATGAACTTGGACATGTTTC

CCCTCTTGAGTCTTGGAATCCATATAGACAATCTCTCTCTCTCCCTATCCAAAACTGAAAACTCTTTCCTTGTTTGTTCT

CTACGTCCGTACAAGTCTCACTCTTCCTTGATCTTGAGCTTTTATTTTACTTGAACTGGAGCCACACCACTGAGCAGAGA

GACTTTAAATCCGCAATATACAAAAGAAACACACATCCCCACCATCGGTACCCACCAATTTTGCCGGA

>JrNF-YA10|Cluster-14922.95251

GGGGGGGGGTTGTCCTTATTATCTCTGCTGAAATTCTTGAAGTGCAACAAACATATTGCTGGCCTTCTGCAGTCTTCCAC

ATGATTGAATGTTTGTAAAGTGGGTCTATGAAGGACTTTTCCATTTTATATGCTTTCTTACCCTTTTTATTTTTATTTTC

ATATTGCCTTTGCTTTTATTATTTTCTACTTTCCTCGTCTTAATGATTACTTTTTAGTAGGAAAGTTTCTCCTCTCCTGT

CTATCAAATGCTAATTTTTAATGATGTCTGTACTTTGAAAGTTTGAAGGCATGGATGACACCTAACGAAGCAAGGATATT

TCAACGTCATTCATTTTTCCAACGAGATGGGAAGAAATTGTGACATTTGCTGGATATTATATTATTTTTTTTATAAGTAA

AAAAGAATTGCTGGATGTTATATTATATATCTTCTGCGGTTTCAAACCTACTGTTCCTGATATGCGGTTGATGTCATGAG

CTGTTTCAAGTTGTGGATTATACCACGCGGTATGATTGATTACAGACTGATTGCTGATTCATCGATGACCTGTAAAATTG

CATCATTTCTGGTCTGTCACTGCATGAAGATGTGTTTTCTTTGCGACTCCATGAGATATTTTTTGCTAAAATGCCCGCAG

ATTCATCATCCCCAAATCATAGGGATGACCCCAACTCGAGTGCCCCTACCTCTCGATCTTACAGAGGATGAACCCATTTA

TGTCAATTCAAAACAGTACCATGCAATTCTCAGACGGAGACAGTATCGAGCTAAGCTTGAAGCTCAGAACAAACTCATCA

AAGATCGGAAGCCGTATCTTCATGAATCTCGGCATCTTCATGCATTAAAGAGGGCTAGAGGATCTGGTGGACGTTTTCTC

AACACAAAGAAGCTCCAAGAAGATGCATCCATAGCCTCTTGCTCTGATGTGATAAGTGCCTCCAACAGTGACAACATCTT

GCAGCCACCAGAATTTAGGTTCAATTGTTATCCTTCTCCTGTTGGTGGGACCATGCGAGGTCATTCAGTTGATATGCATG

GTGGTGGTGGTGGTGGTAAGCAGCACCTACACCTTCCTTCCACCCTCCAATGAGAGATGTGGCAAACCACGCACATCCAG

CAGTCTCTGTTCCATTTTTGGAGGTGGCTGCTGGAAGGGAAGTCATCCTTGGCTATTTTACTTTTTTCTGTTTTACTGCT

TGTCACCTAAGCTTGTCAAAAAACAAAACATAAACCGTTATTGGCTATGTACCATTTTCGCATCGGGGCGTACAAGCACC

TTAAGACTAATGTTATCATGTTAAGTAGGTGATTGTTGATACTTGATAGTAACATATTTGCTCCTTTGAGCAAGACATAA

TGGGAGAAAAAATGAACTGACTATGGAAGCCTACTTTTTCATTATAGTTGGGAATG

>JrNF-YA11|Cluster-14922.46822

TGTTGGCTTCGAAAGACAAAATATAGTTGGATCAACAATTTCATCCACAAGTCAATCAAAATCAATGTTTGAAAAGCTTA

ATGTTCCGCACAATTCAAATCAAATCCTATGAAATGATCAACATAAACAACACAAAGATAGATCATCAAGCCTCCTATCT

AATTAACAAGACGGTTTGTACATTAACCCAAGAGTTTAAAAGGCAAACTTTGAGTCGGCTAATTAATCTCTACCTTTGTA

CAAAGAGCAAAAATGGAAGAAGAGGCTGAAATAAAGAACTGGAAACCTTTTAAGAAGCCGTTCAACTTCTCCACTCTTCA

TCCATGTCACATGCACAATATTAAAGCCAAGAATGATTTGCCGCCTGGCATATAAATTGCCAAGAATGCTGTCTGTAACA

GATTCACAGGGACTGATCCCGTTCCATCATGGTGGGGTCCAATCCACAATCCTGGCACCCAACTCCCTCCCCACAGGAGT

TCATTGTATTGCAAGACGCCTCTGTGAGGCCTGGTTAGAAGAGATGCTTCCCCATTGCTGGCCTGAACAGTCTCCTTCCT

CGCCTCTTCCGCCCATATGCAGATATGATGAGACAGGTAAGACCTCATGATTTTGGTAGCGGTTGTCACCATTTACATAA

TTCTGAGCTTCAGAAGCATCTTGCCTCCCTTCCTGTTGCCCATGAGAGGAATTCCATATTTCAGCAGAGTCAGTGAGCAA

GGGTTCAGAACCAGACGAACTTGCAGACTGTGATGAGAGAGCTGGCCCAGAAGCAATGCCCTTTTCTTTGGCTTCATGGT

TCGCAGCATCAACATTACTTTTCTTTGCAAAACGTCCTCCACTTGCCCTGGCCCTTCTCATAGCATGCTGGTGTCGAGAC

TCATGCAGATAAGGCTTTCTAACTTTTATCAACTTCTTTTCAAGCTCGGCCTTAGCACGTGCCTGTCTCCGCCTCAGAAT

ACCTTGGTATTGTTTGGCATTCACATAAACAGGCTCTTGTGCCATCTCGAGAGGTAAAGGCATTCTGGCATGAGGCATTC

CAAGAAAAGGAGGATAGCCATAAGGCTGATGCCCATAAGCTGCTAACATTCCCCCATGATATGGATCCTGATATGGATTT

GATGCACATGCAATTGAGTGGCCAACAAGTTCAAGCTGCGCAGGTTGTGTGAGGCATTCATTACGCATAGATGGCGCAGT

TGACGCAACATGCTGCATATTTTGGTGGTGTTGTCCATAATTTCCGGCTGATCGAGAAGATGCAGTATTTTGTGATTCTT

TGGTGGCATCGTCACCTTCCTCATTCGGTTCATTATTTGACAGTGATTGACCATCGTTAGAATCTGAACCAGTATCTGGG

CATTCCAACGAAGACGAATTGGATACATTCCCCCCTGTCACAGCAGGGGAGATGGGATTGTACCCAATATTACGCCACCA

AGGTTCAGTATAGACAGCTGGTGGCTGAATGGCATGTGAACGAGGGTCTAATCGATTTGGCTTTTCAGACTTCTGCTGCA

TTCCTTCAAATTCCTTATAAAAGTGTAGGTGCCTTTATTCCTGGTACAATTAAACCCTAGAATTTTGGTCCCGGAGAGAA

TCTGAAAGGGGTCAATCTAATTTCTAATTACTATGCACCCGGCCTCTCTCACACTGAAGCGGCAATACAAGGGCTCTGAT

TGCGTCACCAACACGGCCCCACGTCGGTTAAAACTGCTTATAGTTCCGACGCGTGGAAGAATAGTGACCCGGTACTTACC

GATCCGGTTTTCCGTTTCGTCTGCGGTTCGCGACTAGCGTGGGAATTGAGGAGAGACGGTGAGTGCTGGTTACCGGTTGA

AAATCAAGGGCGCAGGAGGGTTCGAGTCCCGCATGGGTGAAGTTAGCAGAGGCTTTTGGAAGCAG

>JrNF-YA12|Cluster-14922.82902

CCAGGCTTTCTTAATTTCCTCCCAACCCCTTATCTCAACCGACCTATGGGATGAAGGGCTGCTGTTATAGGCAACCAGAC

CTCCATTCAATCATAGATTGATAGGATGCACCTCCTATCTGTGCTTTGCATCTTTATTGTCATCTAACACTCGTCAAATT

GCAATAAAAGCAGAGAGAGAGAGATAAGCTCATAACATTGTCAACATAACACCATCAGGCTAGCAAACTCTTGTCATAAA

CAAGCATCACACTTCTGAAACCTCCATACCTAAATTGTTGAAATTTTACACATACATAACCATTTCCTACTTGATAAGTT

AGGAACTTTGAGCATTTTACACTAGGAGGGAAAGGAAGAAAAATGTATGAACTTCAGACCATAATGGCAGCGAGTCATAT

TTGTCTCCCAATTCTCACCTAGAATTAAGACAAGTAAACTTGTTTTTTTTAAGCCAAGAATGAATTGCCTGAGAAACACG

AGGGTTTCTCCCAAAGCTGGATGCGGCGAACACCTTCTGGTTCACCTGCACAATAGTCTCTCATAAGATACAAAGTTACT

ACGTTCTCGATTACAACTAAACTCAAGAGTCATTAGAACTGAACTGGTAAACTATTTCAGGAGGCATCCCACCTTCAGCG

AATGTCATTTAATGGGGAGTGCCCCATGTGCAACCCCGTTCACCTGCATGCTCTCTCTCTGCTGACCCAAGCAGTCTACC

TCCTTACCATCACTAAACAGTGAATGGTAAGTTGATGACAGGCCATTGCTACTGCTGTTACCTTTGGAAAAACAGTGCGC

TCCATGTGTGTTGTGAACTATGGGCAGTGATCCTTTGTGCTGATTAAGCGAGGAACCTGAATTCCCATTGCCATTGTTGA

AAAAGCATTTAGAGCCAGATGAGATGGTTGGCCCCATAGAAGGTTTAGCACCCATATTCATGCCTTCTTCTGAAGGGGGA

TCAGAAAGATTATCAAGCTTTTTTGTATTAAGAAAGCGCCCCCCACAGCCTCTTGCCCTCCTCATAGCATGTAGGTGTCG

AGACTCGTGCAGATATGGCTTCCGAACTTTTATCACTTTCTTCTCAAGCTCAGCCTTAGCACGTGATTGTCTTCGTCTCA

GAATACCATGATATTGCTTTGCATTTACATAGACAGGTTCCTCTTCCATTTCAAGAGGTAAAGGCATTCTAGCATGATGC

ATTCCATATAATTGAGGTATCATAGCTTGGGGCCCATAAGAAGCAAACATTCCACCATACTGTTGATCTGAATAAGGATA

TGATGTCAATACAATTGAGTGACCAACAAGCTCCATTTGGGAGTTCGGATCAAGGTGTTCAACCATTTTAGGGGTTAAGG

ATGGAACACGGTTATTATCTCGGTGCTCTTTCCCAATATTCCCATCAGATTTTGATGCTACAGTACCCCGTGTTTCTTTG

TTGAATTTGGCTCCATCATCTAGCGGGCCATTCACTTTCAATTGCATGGCTCCATTCCCAACAGAAACATTTAAGTGTTC

CATTGAAGTTGATTTTGAAGCATTGTCACCTAAGGACACATTGCTCCCCACGCCACGCCACCAAGGTTGAGAATATGTGG

CTGACTGCAACACAGTCTGCCCACCATGTTCTAATCGTCCATCTTCATTTCCAGGCTTTGCTGGCATCATTCACAACTTC

CTCGATGCAGCTGATCGGAACTCAAGACGCTTTGGTCTTTCACAAGTCGAAGCTCATCGGGACTGGAGTCTCTCTCTCTC

TCTCTCTCT

>JrNF-YA13|Cluster-14922.52088

CTGAATACAAATTATAACTTGGTGTCTGAGCAAAAGTGTCCCGGAAACTTGTGTATGCATGCAGAGAGGGTACATAACAA

GCATTACGGGAAGGTCAACAATTGCAGCCATTAGGCTACGCAGAAGTGGATGAAAATCAAATATTTCGAAGCCTTCAAAG

TTTTGCAAGATATGCCACAATTCAAATCCTATGAGCAACATTAACTTAAAAGTACACAAAGATGATCAAGCCTCCTATCA

AACAAGATGGTTTCTACATTAACCCATAGTTTAAGAGGCAAACTTTGAGTCGTCTAATCTCTAACTCTGTACAAACAGCC

ATATGAAAGAAGAGGATGGGATGAAACACTGGAAACCTTTTGAGAAGCCACAACATGGCCCACTCATCATCTCTTCTCCA

CCCTTTCATCCATGTCACATGCATAATATAAAAGCCAAGAATGATTTGCCGCCTGGCATACAAATTGCCAAGAATGCTAT

CAACAGATATACAGGAAATACATCCTGTTCCATCAGGGTGGGGTTCTAATCCTCCATATTGACACCCAACTTCTGCAGGG

ATTCATTGTATGGCAAGACGCCTTTGTGAGGACTGCTTAGAAGAGATGCTTCCCCATTGCTGGCCTGAACAGTCTCCTTC

CTCACCTCTTTCGCCAGCATGCAGATATGATGAGGCCTTCAAGCCGTTGTGATTTTGGTAGTGGTCACCACCATTTACAT

AATTTTGAGCTTCAGATGCATCATGCCTCCCTCCCTGTTGCCCATGGGAGGAATTCCATGTTTCGGCAGAATCAGTAAGC

AACGGTTCCGAACCAGATGAACTTGCAGACTGTGATGAGAGAGCTGGCCCAGAACCTATGCCCTTTTCTTTGGCATCATG

GTTCGAAGAATCAACATCACCTTTCTTTGCAAACCGTCCTCCGCTCCCCCTGGCCCTTCTCATAGCATGCTGGTGTCGAG

ACTCATGCAGATAAGGCTTTCTAACTTTTATCAACTTCTTTTCAAGCTCGGCCTTAGCACGTGCCTGTCTCCGCCTCAGA

ATACCTTGGTATTGTTTGGCATTCACATAAACAGGCTCTTGTGCCATCTCGAGGGGCAAAGGCATCCTAGCATGAGGCAT

TCCAAGAAAAGGAGGATAACCATAAGGTTGATGCCCATAAGCTGCCATCATTCCCCCGTAATATGGATCCTGATATGGAT

TTGATGCACATGCAATTGAGTGACCAACAAGTTCAAGCTGTGCAGGTTGTGTAAGGCATTCATCACGCATAGAAGGTGCA

GTTGACGTAACATGCTGCACATTTTGGTGTTCTTGTCCATAATTTCCTGCTGATCGTGAATACGTAGTATTTTGTGATTC

TTTGGTGGCATCGTCACCTTCCTCATTCGGTTCATTATTTGACAGTGATTGACCATCGTTAGAATCTGAACCAGTATCTG

GGCATTCCAACGAAGACGAATTGGATACATTCCCCCCTGTCACAGCAGGGGAGATGGGATTGTACCCAATATTACGCCAC

CAAGGTTCAGTATAGACAGCTGGTGGCTGAATGGCATGTGAACGAGGGTCTAATCGATTTGGCTTTTCAGACTTCTGCTG

CATTCCTTCAAATTCCTTATAAAAGTGTAGGTGCCTTTATTCCTGGTACAATTAAACCCTAGAATTTTGGTCCCGGAGAG

AATCTGAAAGGGGTCAATCTAATTTCTAATTACTATGCACCCGGCCTCTCTCACACTGAAGCGGCAATACAAGGGCTCTG

ATTGCGTCACCAACACGGCCCCACGTCGGTTAAAACTGCTTATAGTTCCGACGCGTGGAAGAATAGTGACCCGGTACTTA

CCGATCCGGTTTTCCGTTTCGTCTGCGGTTCGCGACTAGCGTGGGAATTGAGGAGAGACGGTGAGTGCTGGTTACCGGTT

GAAAATCAAGGGCGCAGGAGGGTTCGAGTCCCGCATGGGTGAAGTTAGCAGAGGCTTTTGGAAGCAG

>JrNF-YA14|Cluster-14922.84458

GCTGCTGCTTTCTACTAAGAGAGGTAGAGGGAGCGCTCTCATTCTTCTCTTCTATTCTTAAGAGTTTGTTCTGGCTAAAC

CATACCGTTTCTGGGTCTTACAAGAATATCCGGCTCATGACTTTGTACCTTTACTTTCTCTGTGGTTTCTATGTTTCACT

ACTCCGACGTATGGCTCGTGTTGGCGTAACGTTTCTGTGCTTTAATCTGGGTTCTATTTTCTTTCTTGTTTGTCTATCTA

ATTTTTGTTCTTTATCAAATGGGTTCTGTGTATTTTTTTTCCCTGAAATTTTGGTTGGCGTTTGTGGAAATCTTGTGCCT

TTTTTTTTTTTTTCTTGAAATACCGATCATCATCATATTCTTATGGGTTTGAATTGATATATGGATATTTCTAACAAAAT

TTGCTCATTTTTCTCTCGTTTTAGTTCCCGCTTTACTGATGTTCCTGTGGATTATATACGAAATTAAGAAATGGGCCTGA

TTGCATCATGGTGTATTTAATTTCTTCAAGATCTTTGGTGTACATAGAGGATAAACTATAGAGCATAAACTATTTGTTCA

TAATCTTTCATATTTTGTTCAATTTGTATTTAACACGGTTAGCTGGGAATTTCGGTTGTAATTTGAATTGCAGATTTGCA

GGGAGTAGAGTGAACCTTCCAAATACAGTTAACCTCAGAAACCTTGCTCGGACCATTTTGGAGCGTTAAATGGCTGCTGA

AACGTTCTATTTCAAACAACGTCAAGGGACTGTCCACAATCCTATAGGGCAGCTGTCAGCGCCTTGGTGGAGTGCCATCA

CTTCTCCATCGGTTTATGGGGAAGCCTGTGTCCAATTGAAGCCTTTGTCCATGGAACACCCCAGCGGTGGAGACCAACTG

ATTACCAACAACCAAGCAGCAAGGGGTACCGAAGCTGCGGCTAGAGCGAATACAACTCGGTTCACTCTCTTTCCCGATAA

TTGCAGAAGTTCAGCGGATGGGCAAAAATCTCAGGTAGCAATTTCTCTCCAATCAGCTCTGCCAGAATATCGAGGTTGCT

TAGAGCTAGGATTTGGGCAGCCGATGATCTATGCAAAGTATCCTTATGGGGACCAATGTTATGGAGTCTTCCCAACTTGT

GGACCTCAAATTCCGGGCCGTGTTATGCTGCCATTGAACATGACAACTGATGATGGACCCATATATGTTAATGCCAAGCA

GTACCATGGGATTATCAGGCGCCGGCAGTCCCGTGCAAAGGCAGTTCTGAAGAATAAGTTGACTAGAGTTCGTAAGCCAT

ATATGCATGAATCACGCCATCTCCATGCTATGCGCCGACCAAGGGGATGTGGTGGCCGTTTCTTGAACACAAATATTTTG

AACAAACGAAAGAATGGAACTGAACTGAACAAAAACGGTGATGGACAAATTTCTCAGGCCACTGGTTCTCAGAGTTCTGA

AGTACTGCAGTCTGATAGTGGTACCTTAAACACTTCAAAGGATGCTAATGGCAGTGGTTCAAATATTTCTGTGTCAGAGG

TGACTAGTATGTTCTCTAGGGGAGATCTTGATTACTTTCCAATCAACCATCTCATGCCTTCTGTCCACTCTCTATCAGGC

AAGATAGATGGTGGGCATGGCATTATTATGCCCGGTAAGTGGGTTGCAGCAGTAGACAACTGCTGCAGCCTCAAAGTCTG

ATAGCAAGGGATTGGGTGGTGTTGGTGCTAAATGCGCCAATCTCCATCCTTGCTGCAACCAGATGGAGAAGTTTCATTGC

AATGACTTGTTCTTGCAACAGGCTTTTGTTGAGGGGGTTGTCCCCACCATGTGGTTGCTAAAAATGGCAATTCATTCTTG

GCTCATGTGCATCGAGGCTGTCTTCTGCTATTCCTTGCAGCCTTTCTGGCTTAAGGTGGTTAGAGATGCCTGATGTACTT

TTATGTACTGTAGATTAATGGAGTAGTTGATAAATAAGTCTTGTTAGCTAATTAAATAAGAGCATCAATGTCTGGATATG

AGTTTGTGAATTAGAGATATTGAAGTGTGATGCTGTTAAAGTTTGATTATCTAGTATGTCTCTGTCTGACTTTTGGAGTG

ATGTATCCTGTAACTTCGCGTCTGTTTTGTTATCTTATACTTTGGAAGCATCAAGCTTTACTCAAGAATTTGGTGTTCCT

GTCATCTGTATTTGAAGCATAATGCCTTATTCCTATTCTTGATTTGCAGTTTGAGAAACACTTTTTCTAAAGATGTAAAG

GATTCATTATTAGCTAGTCGCGTGTGCTTTGCTTGGGTGATAGAATGTGTTTGGAATAACTCAGTGAAGGTGTTCGATAA

TATGAGTAAGTTCTCTGATTCTTTGCTGTGAGGTACAAATTACAATACACGAT

>JrNF-YA15|Cluster-14922.40699

GCTCTCTATAACAGAAACAACCTGGTATTGGAAATTACAACAATTATTATAGATATAAACACTTGGTGAGATATAAAGAT

GAGAATAAATAAGACAACAAAAATTGAAGCCAGAGCATCAACCAACTCAAAAATAAGCTGATCCTACTGCTACATAGTAT

CCATAGTTTTTTTTTTTATTAGTAAACATAATTTTATTGATCACAAGAATAGGCAAAAACCCAGGTATACGGGATATATA

CAAGAGCGTAATGTCGCCTATATATGCGACACACCATTGTTAAGCATTTTCCTCCATCTCTTTCCAAACCATGCCACACA

TGGGAATACAAAACATGTCAATGTTTCGCTTGACACACTTGTGCAAACAATTTAGGAACAGCAAAAGTGATGTATTAGCA

ACCATATCGATGTTCTCTTATTAACACATGCAAATGAAACAGTTTGGTAGAGATTACATCGTGGTATGCCAAAAGCTTTT

TGCAAGACATGTAGGAAACATACAGAAAGTAAAGTAACAAAGCCAAGGATGAATTGCCAGACCAGTAACGTGGTCTTTTG

TATTCGTCTTACCGGACGACTGAAGCACAGTGCTGGGTGCCACCAGACACAAGGCCTCCATTGCATTGCATGGATCCACC

CATGAGGGGAGAGATGCCTGATAATCTAGGATCCCGCAGCTGAAACGTATTGTTATCATTGATATTGGAGATAAGTGTTA

TGTTGGAGCAGGTGGTATCTTGACCAGCCGCAAATCGATCACTATCAAACTCAGACATAGTTTTACTTGAAAGCAAGTCA

GTGGCGGCCGGATCAGGATTATGAAGCTTTCTTGTGCTGACAAAGCGTCCACCGGATCCCCTAACCCTATTTAACGCATG

ACGATGCCGTGACTCATGAAGATATGGCTTTCGAGTTTTGACAACTTTTGTTTTAGCCTCAAGCCTTGCACGTGACTGCC

TCCTTCTAAGAATTCCATGGTACTGTTTCGGATTGACATAAATGGGTCCATCATCTGAAAAATCAAGTGGCAGTGGGACT

CGTGCAGACACTCTCCCCACCATCTCGGGCTGAATAATAGCTGGTAGTCTGTAAGTAGTCAACAAGCATGGCTCGGCATA

GGCATGTGACACTCGAGACATTAAATAGTTGTGAGCAACTTGTGAAGGATTGCAAATAATATATGGATTATTCAATAAGA

AAGGCTTCTTTTGACCTTCCACATCCCTGACACAGCCTTCATCTCCATCAGATTCTGATGAAATGCATTGATCTTGAGAA

TTGCTCCCTCCCGCAGCACCTGCTTTGGGGTGAAATTGACCAGTCAACTGATTTGTAGTTGATTCCTGGATCGGTAGTTG

AAGACCTAAATGCTTTGCATCATGACAAAGTTGTGGAGGAGATCCTGCTTTCAAGCATGCATTTTCAGATAAAGCTGGTG

GAATTCGATGTACATCGGAATTCCACCACAATGGACAATTAGCAGTAAAACGGGGCATTGAATGAACAGGAATTCCGATC

GTTTCTTGGCAAGTCCAGAATTCGTATGGCCTCAAAGTTGTCAATACTTCGGACTTTTATAAAAAAGCCACGGGACCTTC

AACAAGTAGGTTCCTTTCCACTCTTTTAGGGATTAGCTTTGGGATCCACGAAGACATGAAGAAGCAAGGAATAGTACTGT

TGGGATGAACTTGGACATGTCTCCCATCTTGAGTCTTGGAATCAACTCATACACACAAATA

>JrNF-YA16|Cluster-14922.20995

AGAATTCTAAGCATGAAGATGGGAGTTTCACCGCAACATTTTCATAACACCAAGCAATTGAATTTTCAATTCCAAGATCA

GGATTCGTCCTCAACTCAATCAACTGGTGAATCTTATCCTGCAGTGGTTAGTATGCAGGAAAGCTATGCTTGTGGACAAG

GAATAGTTTCACCACAATCAGGATGTATTGAAACTAAGGGGAAGCTTGTTGGAGGTATCATCAAATCTGCCTCACCAATT

GGTGCTCAGGATTTTTTCTTCTTGCCTTCACAGAATGATTACAACCAATCAGTTGTAAGTTGATGCCTCTGTTTAGATAG

TTGTGAGGGGAGATGTGGATCTCTACATGTTCTATGTTTCCTGTTTCAGGCTCACATTCCATTCCCCCATGCTGATCCAT

ATTTTGGTGGTTTATTGTCTGCTGCTTATGGTTCACAATCTATCTTTGAAGGCATGGATGACACCTAACGAAGCAAGGAT

ATTTCAACGTCATTCATTTTTCCAACGAGATGGGAAGAAATTGTGACATTTGCTGGATATTATATTATTTTTTTTATAAG

TAAAAAAGAATTGCTGGATGTTATATTATATATCTTCTGCGGTTTCAAACCTACTGTTCCTGATATGCGGTTGATGTCAT

GAGCTGTTTCAAGTTGTGGATTATACCACGCGGTATGATTGATTACAGACTGATTGCTGATTCATCGATGACCTGTAAAA

TTGCATCATTTCTGGTCTGTCACTGCATGAAGATGTGTTTTCTTTGCGACTCCATGAGATATTTTTTGCTAAAATGCCCG

CAGATTCATCATCCCCAAATCATAGGGATGACCCCAACTCGAGTGCCCCTACCTCTCGATCTTACAGAGGATGAACCCAT

TTATGTCAATTCAAAACAGTACCATGCAATTCTCAGACGGAGACAGTATCGAGCTAAGCTTGAAGCTCAGAACAAACTCA

TCAAAGATCGGAAGCCGTATCTTCATGAATCTCGGCATCTTCATGCATTAAAGAGGGCTAGAGGATCTGGTGGACGTTTT

CTCAACACAAAGAAGCTCCAAGAAGATGCATCCATAGCCTCTTGCTCTGATGTGATAAGTGCCTCCAACAGTGACAACAT

CTTGCAGCCACCAGAATTTAGGTTCAATTGTTATCCTTCTCCTGTTGGTGGGACCATGCGAGGTCATTCAGTTGATATGC

ATGGTGGTGGTGGTGGTGGTAAGCAGCACCTACACCTTCCTTCCACCCTCCAATGAGAGATGTGGCAAACCACGCACATC

CAGCAGTCTCTGTTCCATTTTTGGAGGTGGCTGCTGGAAGGGAAGTCATCCTTGGCTATTTTACTTTTTTCTGTTTTACT

GCTTGTCACCTAAGCTTGTCAAAAAACAAAACATAAACCGTTATTGGCTATGTACCATTTTCGCATCGGGGCGTACAAGC

ACCTTAAGACTAATGTTATCATGTTAAGTAGGTGATTGTTGATACTTGATAGTAACATATTTGCTCCTTTGAGCAAGACA

TAATGGGAGAAAAAATGAACTGACTATGGAAGCCTACTTTTTCATTATAGTTGGGAATG

>JrNF-YA17|Cluster-14922.27809

CCAGGCTTTCTTAATTTCCTCCCAACCCCTTATCTCAACCGACCTATGGGATGAAGGGCTGCTGTTATAGGCAACCAGAC

CTCCATTCAATCATAGATTGATAGGATGCACCTCCTATCTGTGCTTTGCATCTTTATTGTCATCTAACACTCGTCAAATT

GCAATAAAAGCAGAGAGAGAGAGATAAGCTCATAACATTGTCAACATAACACCATCAGGCTAGCAAACTCTTGTCATAAA

CAAGCATCACACTTCTGAAACCTCCATACCTAAATTGTTGAAATTTTACACATACATAACCATTTCCTACTTGATAAGTT

AGGAACTTTGAGCATTTTACACTAGGAGGGAAAGGAAGAAAAATGTATGAACTTCAGACCATAATGGCAGCGAGTCATAT

TTGTCTCCCAATTCTCACCTAGAATTAAGACAAGTAAACTTGTTTTTTTTAAGCCAAGAATGAATTGCCTGAGAAACACG

AGGGTTTCTCCCAAAGCTGGATGCGGCGAACACCTTCTGGTTCACCTTCAGCGAATGTCATTTAATGGGGAGTGCCCCAT

GTGCAACCCCGTTCACCTGCATGCTCTCTCTCTGCTGACCCAAGCAGTCTACCTCCTTACCATCACTAAACAGTGAATGG

TAAGTTGATGACAGGCCATTGCTACTGCTGTTACCTTTGGAAAAACAGTGCGCTCCATGTGTGTTGTGAACTATGGGCAG

TGATCCTTTGTGCTGATTAAGCGAGGAACCTGAATTCCCATTGCCATTGTTGAAAAAGCATTTAGAGCCAGATGAGATGG

TTGGCCCCATAGAAGGTTTAGCACCCATATTCATGCCTTCTTCTGAAGGGGGATCAGAAAGATTATCAAGCTTTTTTGTA

TTAAGAAAGCGCCCCCCACAGCCTCTTGCCCTCCTCATAGCATGTAGGTGTCGAGACTCGTGCAGATATGGCTTCCGAAC

TTTTATCACTTTCTTCTCAAGCTCAGCCTTAGCACGTGATTGTCTTCGTCTCAGAATACCATGATATTGCTTTGCATTTA

CATAGACAGGTTCCTCTTCCATTTCAAGAGGTAAAGGCATTCTAGCATGATGCATTCCATATAATTGAGGTATCATAGCT

TGGGGCCCATAAGAAGCAAACATTCCACCATACTGTTGATCTGAATAAGGATATGATGTCAATACAATTGAGTGACCAAC

AAGCTCCATTTGGGAGTTCGGATCAAGGTGTTCAACCATTTTAGGGGTTAAGGATGGAACACGGTTATTATCTCGGTGCT

CTTTCCCAATATTCCCATCTGATATGAAAAATAAATTTGAATATTAACCAGATACTCTGCACTTGTGACATACATGTTCA

TAAAGCAGAGAACAACGACCTGTTAAAATTTTAATTCTAGGATATAAGAAACATTCCATCCACACCTTGGCACAACATTG

CATTTGTGAAGTTCGTGTATCTCAGATCACATGAATATGTAGATCTATATTTTCCCCCTATTCTGGGATACCTGGTGGTG

TAAGTCAACATATCTTCCTAGCAGTATTTCATAAGAGGTTCACATAGAAAAAACAATGACATAGAGTTTGGGAAGGACCC

AGTAAGGGACCATAAAATAGGAAAAGGATAGAAAAGAGCTGCTTGAATTTCGCATTAGGACCAAAAGCACAGTTTAAACA

TTTTTTTTTATTGGTTCTGAAGCCGTTCCATTTATGGACAACTCGTGTAACAAAGGAAAAGATGACAGTCACGGAAAAAA

GGGGAAGTTGGAAATGGAATAAAAAACACAGTTTAAACATTGGAAGCCCATGAATACTTAACAGCCTGAAGCCTTTGTTC

CATATATGGAAAACTCGTGTAACAAAGGAAAAGATGACAGTCACGGAAAAAAGGGGAAGTTGGAAATGGAATAAAAAACA

CAGTTTAAACATTGGAAGCCCATGAATACTTAACAGCCTGAAGCCTTTGTTCCATATATGGAAAACTCGTGTAACAAAGG

AAAAGATGGCAGTCATGGGAAAAAGGGGAAGCTGGAAATGGAATCATGATTGATCAGCAATTGATGGTCTCGGTCAAAGA

TGGTCCACATCTTGACCTATTTCACTTAC

>JrNF-YB1|Cluster-14922.21265

CTCCATACATGCTTCGCATACAGGTAGACATGCACACACATACATGCTTCTATATCTACTTCTATATAGCAACCAGAGAA

TAGATCATCGAAAGCAACGTGTCTGTGTTTGAGAGGACCAAATTAAGCCTGATCTCCTCATTATAGAGATCGATATATGG

CTGATAACAGGGGCAATAACTTAAGCATTAGGGAAGGATTTAAGTACCATTTCGATGGTGCTAGCAGTACTGCTTCGGGT

GAAGATGCGCCCATGAAAGAGCAAGATCAGTTGCAGCTCCCCATAGCTAATGTTGGGAGGATCATGAAGCAGATTCTTCC

TCCAAACGCCAAAATCTCCAAGGAAGCCAAAGAAACCGTGCAAGAGTGTGTTTCCGAGTTCATCAGCTTTGTTACTGGAG

AAGCCTCGGACAAGTGTCACAGGGAGAAGCGCAAGACAGTGAACGGGGATGATATCTGTTGTGCCATGGCAACCCTAGGA

TTTGATGACTATGGAGAGCCGTTGAGAAGGTATTTGGAAAAGTATAGGGAGTTGGAAGAGGAGAGAGCCCAACAAGGCAA

GTTCTGCAACAGTACTGAAGAAAACAACATGAAGCATGGTAACAATCTCTAGTACTCTTATATATATTTATAAACATTTT

GATGCTGATCATGATATCTTAGACACTTCGTCATGTTCACATAGTAGGTTGGTGGGGGTGGGGGAGAGGGCTGATTTTAG

GTACTACTTGTGTGCAAACTGATTATTGACTATGGATCTTCAATTGATCACTGTTCTTAGCTTAGGTATTATATGAAATT

AATCAAATTCTTGTGTCCTTTTTTCTGGATTAATTATGAGGTTAATTTGTAGCATGAAAC

>JrNF-YB2|Cluster-14922.29372

CAAAAAAAAAAAAAAAAAAACTCCCGGCGGATGTTATAATTGCAAAACAAAAGAAACCCAAGTTTTCTCTTTCTTTTCAT

TTTCCCGTTTCTTTTTCGCAGCAACCAAGAACTCAGATAGATGTACGTAAAACAGTCATATTTAAAAAATGACCTCTCTG

TATCTTGACAGGTAAATCTTAAGCGGATCGATGTAGTCTTCAAACCCTAAAGTAGCCATAGCCCAAAGCAAATCGTCCCC

ATTAATTGTCTTCCGCTTCTCTCTCTGGCACTTGTCACTGGCCTCGCTGGTGATAAAGCTGATAAACTCAGAGACGCACT

CTTGCACGGTCTCCTTGGCATCCTTAGCGATCTTTCCGTTAGCAGGGAGAGCCTTCTTCATAATCCGGCTAATATTAGCG

ATAGGGAGGAACCGGTCCTGCTCGCGAACATTGGAGCGGGGGCTCTGCTCCCCGCTCTCAAGGCTGCCTCCTCCGTTTGG

ACTGGTCGGAGCATCGGCCATCCGAGCCAAAACCCTA

>JrNF-YB3|Cluster-14922.32115

AAGGCATTAAGGTTTCATAATCATTTCCATAACATCTTTAAAAAAAAGGGTCTAAAAATGACATGAACAAAAAAAACATT

TACAAATATTCTGTTCAAATAGTCATTGTATTCTAAGACACATAAATATATGATGTTACCTGCCATACTTCTGGAGATGA

GTTCAGAATAACCCTAAAATGTCAGTGTCACACATAAGAATAATGTTTCATTATGTTTTGATAACAAGATATTCCTAAAG

TAGGATACCAAATATTGAAATTGGACGAAGATCAGCCAGATGCCTATTCAGTGCCTTGCATAGGAACCATCAGATGTTGG

GCCACCTGAGAACTAGAGTAATTCATGCCTTGTGAGAAAGAACCTTGATGACCAATTTCCACCTGCACATTTGGACTTAA

TTGAACATTTTTCTTAGAAGATGCATCTCCACCCTTTGCTGGCCCCTTGGTGTCACCCTCAATCTCTCTGTATCTTGACA

GGTAAATCTTAAGCGGATCGATGTAGTCTTCAAACCCTAAAGTAGCCATAGCCCAAAGCAAATCGTCCCCATTAATTGTC

TTCCGCTTCTCTCTCTGGCACTTGTCACTGGCCTCGCTGGTGATAAAGCTGATAAACTCAGAGACGCACTCTTGCACGGT

CTCCTTGGCATCCTTAGCGATCTTTCCGTTAGCAGGGAGAGCCTTCTTCATAATCCGGCTAATATTAGCGATAGGGAGGA

ACCGGTCCTGCTCGCGAACATTGGAGCGGGGGCTCTGCTCCCCGCTCTCAAGGCTGCCTCCTCCGTTTGGACTGGTCGGA

GCATCGGCCATCCGAGCCAAAACCCTAGTACACAGCACCAATCACAAAACGACATCGTCAGTGAGGACCAGAAACCCCAA

GCCCCTCGGTGGCACCAAAACCCTAGAGAGAGAGGGGGTGCGGGGGGGTGAATTTCATGGCGTGCCTCTGTGCGTGCGTG

CGTGCGAGATGGAAAATGAAAAATGCGAGGGAAATCGTGGTGATGGCACTGATGGGTA

>JrNF-YB4|Cluster-14922.39672

ATGAGAGGAAACCACATATAAGAAATAAAAAAGAGTACTACTTTGCACATATTCTCATGGAAATCACATCTCAAACCTTA

TGATTCACAATCACAATCATCAATCCATATCCTTTTGTTTTCACAATATAACTAAATTAGAAGAAACCTGAACTATATTT

ATCTACACTTTACATACAACCTCTTGTAACCTTAAATTCAATCTCTACCCCATATTTACAAAAACAAAAATCCAAAAGGA

TTCAAGATTAAAGAAAAGAAAAATTCTTTTCATCAGTCACTATTCACTATCTTATACTTTATAAAAAACATTCTCATACA

CTATTAGAAAAATAAATATAAATATAAAATATGAAAATAAATTGTGACTGATGAGTAAAATTTATCTTTAAAAAAAACAC

ATATAAATTCATAAAAAGCTCTATTACAGTGGAGTTGCCCTCACTTGGATATATATTTAAAATGTTTGATAAATAGCTTA

AGGGTCCCACATTAACTATTAATGAAAATAATTATCTGGGCCTACCCGTATTCGAACCAGGCCCAACATAACCGGTCCCA

CTCTTCCCCAACCCGCCAAAACCACTACCGGTAGTACCGATCTGCTGATGAAACCCACTGGAGCCGTACACGTGTCCACC

CTGATGCATCATCATTCCCATCCCGCTACTACTCCCATATACCCCGCCACCCCCACCCGGCTGTTCGAACCCACCACCGT

TTCCAAAACCCGTCGAGGACGCGCCTCCGGCAGAGGCGTCCTTGTCACGCACCGCCACGCTCTTCTCTCCCTCCATCTCC

CTGAACCTCTGCAGGTACACCTTGAGGGGCTCCACGTAGTCCTCGAAGCCGAGGGTGGTCATGGCCCACAGCAGATCGTC

GCCGTTGATCGTCTTTCGCTTCTCCCTCTGGCACTTGTCGGATGCCTCGCCGGTGATGAAGCTTATGAACTCCGACACGC

ACTCTTGCACTGTCTCCTTGGCGTCCTTTGAGATCTTCGCGTTTGCCGGCAGCGCCTTCTTCATGATCCGGCTCACGTTC

GCAATCGGCAAGAACCGGTCCTGCTCCCGCGGTGACAGCTCATTGGCCGACGCGTTTCCAGCTCTTTCACCGCCTCCTTC

GGACTCGTTGTCTGAGTCCGCCATTTAAACTTGAAGGTTTTTATTGCTGCAGTATGGAGAAAGGTATGAAGAAGTGGAAA

TTTCTTCACTCTCTCTCTCTCTCCCTTTTAGTGAGAGAAAGTGGCCATAATGATAACTTCTTAGATTAATTTTTCATGAA

ATGAAAGAACTTTTTATCTGTAGAAGAATTAAAAGTACTTTTGAACGCTAAAATTTATACGAAGTTAAGCAAATGAGTCG

GCTACTTGCAAATTCTGTCTCTTCTGATAGAAAAATATTGAGAAATCGAAGTCGGTAAGTATTTCAGAATGCAAACTTTG

AAACTGCACCGAAAGAAACAGAGGAAGAATCGCCGACGTCGAGGAAAAAAAGAAGAACAAACAAAACAGAGGAAGAATCT

ACGGTTTTGAGTCGCGAAAGCTCGAGGCTTTGAAGTTGGATGAAATTGCCTGAAAAGAAAGAGATGAGTAGATGAGCGGA

GGAGAGGGAAGAAACTAGTTATATTGCTTTAAAACATAGAAAAATTGAGTAGTTTTTTTAGTGCAATGAGAAATTGCAGC

ATCA

>JrNF-YB5|Cluster-14922.54864

AAGGCATTAAGGTTTCATAATCATTTCCATAACATCTTTAAAAAAAAGGGTCTAAAAATGACATGAACAAAAAAAACATT

TACAAATATTCTGTTCAAATAGTCATTGTATTCTAAGACACATAAATATATGATGTTACCTGCCATACTTCTGGAGATGA

GTTCAGAATAACCCTAAAATGTCAGTGTCACACATAAGAATAATGTTTCATTATGTTTTGATAACAAGATATTCCTAAAG

TAGGATACCAAATATTGAAATTGGACGAAGATCAGCCAGATGCCTATTCAGTGCCTTGCATAGGAACCATCAGATGTTGG

GCCACCTGAGAACTAGAGTAATTCATGCCTTGTGAGAAAGAACCTTGATGACCAATTTCCACCTGCACATTTGGACTTAA

TTGAACATTTTTCTTAGAAGATGCATCTCCACCCTTTGCTGGCCCCTTGGTGTCACCCTCAATCTCTCTGTATCTTGACA

GGTAAATCTTAAGCGGATCGATGTAGTCTTCAAACCCTAAAGTAGCCATAGCCCAAAGCAAATCGTCCCCATTAATTGTC

TTCCGCTTCTCTCTCTGGCACTTGTCACTGGCCTCGCTGGTGATAAAGCTGATAAACTCAGAGACGCACTCTTGCACGGT

CTCCTTGGCATCCTTAGCGATCTTTCCGTTAGCAGGGAGAGCCTTCTTCATAATCCGGCTAATATTAGCGATAGGGAGGA

ACCGGTCCTGCTCGCGAACATTGGAGCGGGGGCTCTGCTCCCCGCTCTCAAGGCTGCCTCCTCCGTTTGGACTGGTCGGA

GCATCGGCCATCCGAGCCAAAACCCTAAGTTTGGGACCGAGCGCGAGAGAAGGACGGGATGGCTTAAGATCGGACGCTGG

AGAAGGAAAGAGAAAGGAGACGACTGAGATGAGAGACAGATTTATCAGCACCGTCCGTTTCGGTTGATTTTCTTTGGTGG

>JrNF-YB6|Cluster-14922.57314

TAGAGATCCACGTGTTGCGCTCGATGCAATTATTACCCTAACGATCCAACGGACCCCAACACCTCTCCACCTCCAGCTCT

GCAACTCCCACCCACATTCCCGGAGCTCCTCCTTTCCCGGCCGTTTTCCCTATCCTGTCATTCACCTTTCCAACCAATAC

GCTCCAAATCCCTCCCCCTCGAGCCGTATTATCCGATTCCGTGTATAAAAAACTTTTCCCTTCCATTTGGTCCAATTTCC

TTCGAGTTTGTAATTCTTTTTCTTTGCTTTCTGTTATTAAATTTGTTGAGCCTCGAAAAATTGTTGACTTGGTTCTTTTC

AGCTGAAAGTTTTTGTATTTTGGTCCATAATTTGTATACTTAAGTTACATCTGCTCGGGCCTTAAATCTTTTTCAGAATT

CAGTCTATATTTTCTAGGTTCGAAGTTTTGGATTTGGTCTATTTCGATTTTGTAGAACTAGAGAGTTTATTACTGTAATT

TGGGGAATTTTCAGTATATCCATATAATTTCACAGCTTGAAAAATTTTAGACGTGGATCATTTTTTTCCCTGAAACTTAG

TTAGAACTATATGACTTTCTTGCTTAAAATCTGTTGAATTGGGTCTATTTCGTGTAAATTTATCAGAGATCCAATACTTG

ATGGCTGAAAGTTGGTGAATTTGATCCATTTTGTGGAGTTGCATCAGACCCAGAAAAGTTTCAAGCTCAAAGTTGTTGAA

TTGGGTCTAGTTTGTGAAGCTCAACCAGACATATACAAGGCGAAAAGGTCGATGGCTGATTCGGACAACGAATCAGGGGG

GCACAACAACAGCAACAGCGCGAACGGCGACTTCGCTTCTCGAGAGCAAGACAGGTTCCTCCCAATCGCTAACGTGAGCA

GAATCATGAAGAAGGCATTGCCCGCAAACGCCAAAATTTCAAAGGACGCTAAGGAAACCGTGCAGGAATGTGTGTCGGAG

TTTATCAGCTTTATAACTGGCGAGGCCTCGGATAAATGTCAAAGGGAGAAGAGGAAGACGATCAATGGGGATGATTTGCT

GTGGGCGATGACTACTCTGGGGTTTGAGGAGTATGTGGAGCCGCTGAAGATATATCTGCAGAAGTATAGGGAGATGGAGG

GGGAGAAGAGCACGGTGGGGTCCCGACCAGGGGAAAAGGATGGTGGCGGTGGGTCCGGTGGTGGTGGAGCCGCCGCGGGA

GGTGGAGGGAGTGGTGGGGCGGTGAACTCGGCGAGTAGTGGTGGTGCTGGTGGGTTTAATGGGGTTGGGGGAGTCTATGG

TGGGATGTATGGGGGGGTCATGGGCGGTCATCATCATGGACACATGTACGGCTCTGGTGGGTTTCATCATGTGGGTGGTA

TTGGTGGTGGTGTTGTCGGGAAAGATGGTTCAGGCGGTGGCGGCGGTGTTGGAGGGTCCATCGTGAGATCAAGGTAGATG

TGCTTTCATTGTTTAAAGAAGAAGGGTGGATATATAAAATGACTGCGTTGATTTAGGGGTATTTAGTTTGCTCTTAATTC

CTACCGCTAATTATAGCCCAAAACCTTGTGAAGTTTAGAGATCAGATGATCTTATCTGGACCGGGGATGGACCCTATTGG

GTTTTTCGTTAATTTTCTTTCTTTGGACTTTTTGGTGGGGATGTGGTACTTGTTTATGGAATTGTATGTGTAATATGGAG

TAGTATATGTGTAATTGTGGATCAGAAGATACATGATATGATGCATGGTATTAAAAACTGTAACCTCTAATGATAAAGCA

GTAGCTCTGAGGAAAGAGGAAGCTGCATGGAAAATACTTTGTTGTAGTATATGGATTCATTCACTTGTAGAGTTGTTTGT

TGTCCGTGTTGTTCTTAAAAAGGGTTTGGATAAATGATTTGGAGGTTTCCTATGTTTATAGTTTCATGGCATATTGAAAA

TTTGGGAATGCTTTGAACTGACAGTTTTATTTTGAAGTTTCGACAGCTTCTTTCTTTCAGAAAGGGAGGGTTTGCATGCA

TGCATGCATGCATGCATATTTCTTTTGGGGAACGCGGCATGTGTCCAGAGTTTCAGCCATTTGTCTCGATGGTTACGTGT

CTACTAAATAGGGAGCTCTTTAGATTGGCTTCTATATGTCATGAAATCTGTCGTTGCTTACCATATATTTCTATTTTATG

TTTTTCGTGCGGTGGGAGGACTGTAAGTTGGTTAGTACTGCTGTGATTCATTCATTGTCTTGCATGCGGATACATACATT

TATGGAATGGAAGGATTTTGGGAAGTCAGATTTTGTGGTTCTGATCTTCTTCTGGTTTCTGAAGAATAATAGAATTACTT

GCATGTAGAGCTAGGAAACATATAAACAAAACAAAACAAAACAA

>JrNF-YB7|Cluster-14922.58354

AGAGAGAGAGAGAGGGAGAGAGAACCTCCAAATGCCAAATTCTTCTTTGGGGTCAAGGCAAGACATGCAGAAAACTTCAT

CAATCACCAATCATCCAAAACCAAATATAAGTGTGGGCCCTCTTACCAAATCTCCACATATTCCAGCAGCCAACAATGTC

AGGCAACTATAACTTAGAGCAAATTTACAGGGCTCATCAGACTATAAAATCTTGTTCAGGTGACGAGACCAATCCTAAAA

AGGTCTCCCCTCGATTCGTGACAATAACCAAAACAAACGCCCCACATATAGTATTTTGGCTAAAAATAAAATTTATAAAA

ACCACATAAATTAAGCTATTGGTGCTGAAACCATATAATTTAACCCGTCGTACAATCCACAAAGACAGTTAACATAAAGC

TAGAACTTCCTACTCGTTTCCTTGCACAGAATGAACACCCATATGTCGTCCATGTACCCAATATTACTCCTGCCTTGGCA

GGAGTCTGGCAGGGAAGATCTGGATATGGTCCTAACCTTCAGCATCAGCAAATTGGGAGTTCATGTAGTTCAATGACCCC

TGATGAGCAAACTGTGGGTTTTGAGGGGCCAGAGCACCAACCGCATCCCTTTTACCAGATGCATCTCCACTCCTAGCAGA

TCCTTTGGAATCACCCTAATCGAACTCATACCAACAATGTAATTCCTAATATTACTGGAAAGATACTGAGGAAAAAACTA

TAGACCTGTTTGGTCGTGAGTTCGAGAAAACAATTGATTAAAAAAATACTCTCTTTTTATCATGTGATAAACAAAACTTA

CAAATATGATGTCTTCACTTGTTTTACTTTTCCAAAAATGATAAAAAAAGAAAACATCATGCTAATGTTCTGTACAAGCA

GGTTATTCAAATATAACTCTCTGAAAACCCTTTGGTGCCACCATTTTTTACCGTTGTCTTTTTATTTCTTTTGAGGATTT

AAAACATAAGACTGCTTAGAATAACAGGAACCAAACAGTCTCTAGATACTCAAAGTTAGCAGATACTCAAAGTTAGCTAA

ACTAAGTGAATTTATATTAGGGGAAGAGAGTGGGTTTGCTTACCTCTAACTCTCTGTACCTAGCTAGGTACACCTTAAGT

GGCTCGATATAGTCTTCAAACCCCAACGTCGCCATTGCCCACAACAAATCATCGCCATTAATGGTCTTCCTCTTCTCCTT

CTGGCACTTATCGCTCGCCTCGCTGGTGATGAAGCTGATGAATTCGGAGACGCATTCCTGCATGGTGTCCTTGGCATCCT

TGGCGATCTTGCCGTTGGCCGGCAACGCCTTCTTCATGATCCTGCTGATGTTGGCGATCGGCAAATATCGGTCCTGCTCG

CGCACCCCCGCCGAGCGCGGGCTCTGATCGCCGCCGCTCTCGTGGCTCCCACCCGCTGGACTCGCCGGAGCCTCCGCCAT

ATCCGCCCCCAAAAACCCTAGATCACTGTGGAGAGATGAAGAGAAGGAGGCAGAGACGTCGCGGTTTTGTTAGTTTTGTA

TTTGGCGCGCGTTGGGGAGCGACAGAGAGAGAGAGGATTGGTGGAATTGTTTTTTGATGGAGAGAGTGATCGGACGCTGG

AGAAGAATGAGAGAGAGAAAGAGAGTGAGGAAAGAGAGGAAGAGAGTTGGATCAGAGAGCGCGAGCCTTTTGGCCTTGAG

CTTATCACCACCGTTGGTTGCAGTTTCCCATTTACATTTTCGTTTCCTTATTTATAGGGCCTACTATGTCTACT

>JrNF-YB8|Cluster-14922.64236

GGTTATGGAAAGCTGTGGGAATTTTAGTTAGACATCGATCCCATGACTTCAACTGCTACTACTCACAAACCTAGCTAGCT

ACCTTCAATATCCCACATATAATTCCCACAGCTTTCCATACCCTACTGCTCTCTTTGATCCAAAACCCTATATTTACAGC

CAGTACCGTACGTGTTTATCTCTTCATGCACTGTTTCTCCTGTTTTTTTGTGAGGCACATGAGATAGATCCAGATAAGTC

TCAAGCTAGAGAATTTTGACATCTTCTTGGCCTGCCCGGCTTGCTTTATACGTAGTACTGCCAATTTAATTGGTTGTGTG

GCCGCGCCAGCTCGCTCATGGCTGACTCCGAGGACGAGTCTGGAGGAGGGCAAAACAACGCAAACTTCAACGGCGAGTAC

TCTTCCCTGAGAGAGCAGGACAGGCTTTTGCCTATAGCAAACGTGAGCAGGATCATGAAGAAAGCGTTGCCGGCCAACGG

GAAGATCTCCAAGGAAGCTAAAGAAACCGTACAAGAGTGTGTGTCGGAATTCATCAGCTTCATTACCGGAGAAGCCTCCG

ATAAGTGCCAGAGGGAGAAGAGGAAGACCATCAACGGCGACGATCTTCTGTGGGCCATGACAACGCTGGGGTTCGAAGAG

TATGTGGAGCCGTTGAAGATTTATCTGCAGAAGTTCAGAGAGATGGAGGGAGAAAAGAGTAGCAGTACTACTAGTACAGC

TGTGGCCGGCGGAAGGCAAGGGGAGAGGGATATTGGCGGCGATGGCAGTGTTGGGAATAGTGGGAACTCTGGGGTTGGGA

TGATGTATGGTGGGGTGCAATCCACTATGATGATGATGGGTCATAATTATCATGGTGGCCATGACATGTATGGATCATCA

GTCAGTGGAAGTGGTAGTGGAGCCTCCTCTGGAAGGCAAAGGTAGGTCCAATTGTTTCAAGGATAATGCTTTTTTTTTTT

TTTTGGGTATGCATGCATGATAGAAGGATGATGATGATTTCAATTTGATAACTTTGTTGAATGTTTACTTAGTTGATGAG

TCTGGAATATTATGGATGATTACATATATAATATATTAATATATTGGTATGCCATGGTTGATGTTGATTAGAGGGAG

>JrNF-YB9|Cluster-14922.72337

TCTTGAGGTCATAACCCCAAAGAGAGTGTGAGAGATCAGAAACAGGAGATCAATGGAAGATGAGAGCCATGGAAATGAGC

TCGATGGAGGAGGAAGCCCAGAAAGCCCATGCCTAAAGCGAAGCACTAGCAACATTAACATGAAAGAACAAGATCGTTTC

CTCCCTATAGCAAATGTAGGCAGGATCATGAAGAAAGTGATTCCGGCCAACGGAAAAATATCAAAGGACGCAAAAGAGAC

AGTCCAGGAATGTGTTTCTGAGTTCATTAGCTTTGTCACCGGTGAAGCATCTGATAAATGCCAAAGAGAAAAGAGAAAGA

CCATCAATGGCGACGATATCATATGGGCTATCACAACCCTAGGGTTTGAGGACTACGTGAGCCCCCTCAAAACGTACCTC

CAAAAATATAGAGAGATTGAAGGAGAGAAGCTTAGCAATATTCCAAAGCAGCAAAGGCAACAACAACAACACAAACAAGA

ACAAAATATACCGTACGATGATGATACTCATGTATATGGCCTAGACAAGAACGATAATAGAACAGTATATTCTTCCATAA

GTGATCTTATGTCTCAGCCGCCTTTCGTGGCCACCGATCAACCATTCTCATTGCATTTCTCCCCAAGTTCAGTTCAAAAA

CAATTACTGCCACAAGACCAGATTAATGATCATTGGGGCATTGGTAATTAATTAGCTTGAAGA

>JrNF-YC1|Cluster-14922.36868

CAACCCTTAAAATCCCCACCCCACATTCCCTCTTCATAGTTCATCCCTCAAATCCCACCCACCCATCATCATCCCAGAAA

AACACACGCACTTGCACACGCAGAGTCCCCCATTTGGCAGCATTTCTCGCAGACCCATCATCAAGCACCTTTCCATCAAA

CCCACAAATCCCCCCCTCCTACCCATCAATCAATGGAGAACAACAACCAGGCAGCCCAGTCCTCCTCTTACCCTCCCCAA

CCCACCCCTCCTCCAGCAGCTCCATTCCACCACCTCCTCCAACAGCAGCAGCAGCAGCTCCAGATGTTCTGGTCCTACCA

GCGCCAGGAAATCGAGCAGGTTAACGACTTCAAGAACCACCAGCTCCCTCTTGCCCGTATCAAGAAGATCATGAAGGCCG

ACGAGGACGTGCGAATGATCTCCGCCGAGGCTCCCATCTTGTTCGCCAAGGCCTGCGAGCTCTTCATCCTCGAGCTCACC

ATTCGGTCCTGGCTCCACGCCGAGGAGAATAAAAGGAGAACCTTACAGAAGAACGACATCGCGGCGGCGATCACAAGGAC

CGACATATTCGACTTCCTGGTGGATATCGTGCCGAGGGACGAGATTAAGGACGAGGCCGCCGGGCTGGGCGGGATCGTGG

GGCCCCCCGCCAGTGGCGTCCCGTACTACTACCCTCCGATGGGGCAGCCTGCGGGTGGGCCCCACGGGGGGATGATGATT

GGTCGGCCCGCGGGGGCAATGGACCCTTCTGGGGTGTACGTGCAACCCCCCTCAAACGCATGGCAGTCCGTGTGGCAGGG

CCACGCTACCGACGATGGGTCGTATGGGAGCGGAGGGAGCACTGGGCAGGGCAATCTTGACGGCCAAAGTTAAAGTTGCT

TGTTTGAAGATGTGCTGTGGCGCAGTTGTTCCATGTAGTTAAAAAGTAACTTGTTTCGAAGATGTGGGTTGTGGCGCTGT

GGAAAGGTGTCAGGGTGTAGTTGATGCTACTTGGAATTTTTGATTGCTTCTGAATTATAAGATGCACTTGTTGTCAGCTG

TTATTCTTTATGGACGCCTATAGAAACTTGCTATAATTTTAACTTGATCCGATGCAGTTGAACAAATATGCTGCTGTGAC

AACTATTTTAATACTACTACTGTGCAGTCTTCAGTTTAAGGATCCTCGTGTTGCTTATTTGAATGATTATGGCTGTCTCG

TGA

>JrNF-YC2|Cluster-14922.71538

AGAGAGAGAGAGAGAGAGAGAGTGCGGAGAAGAATCGGGAATCGGGACCTCTCAGAGACAGCAGTACCTAAAGTCAATCT

TCTTCCACTACCTCAACTCCACTTCTCTTTTGGGGTTTCAGCTGCCATTGAGAAGACCCGGAGCCGGTGAAACCTGGACC

AATAAAACGCAGCGACTCAGCATCAGAAGTGGTAGCATAAAGAATTTCCTCCAGGCAAACCTAAGCTAGTATGGATCAGT

TAGGGCATGGACAACCCCCAGCAATTGGGGTGGTTGGTAGTGCAGCTCAAGTGTCATATGCCCTCAATCCATATGAATCT

AACCAAATGATTGGGGCCTCCCAACCGGGATCGGTGGGATCCATGCAGTCTCCTAGTCAGACAGCAGGTCTCTCTGCCTC

TTCAGCTCAGCTTGCACAAAACCAACTTGCTTATCAGCATATCCACCACCAACAGCAACAGCAACTGCAGCAACAACTCC

AAAATTTTTGGGCAAATCAGTACCAAGAAATCGAGAAGGTAACCGATTTCAAGAACCATAGCCTTCCTTTAGCAAGGATA

AAGAAGATTATGAAAGCTGATGAGGATGTAAGAATGATATCAGCTGAGGCTCCGGTAATATTTGCCAGAGCATGTGAAAT

GTTTATATTGGAGTTGACCTTGAGATCTTGGAATCATACGGAAGAGAACAAAAGGAGGACACTTCAAAAGAATGACATCG

CAGCAGCAATCACGAGAACTGATATCTTTGATTTCTTAGTTGACATTGTGCCAAGGGAGGATCTGAAAGATGAAGTACTG

GCATCAATCCCAAGAGGAACAATACCTGTTGGGGGGCATGCTGATGCTCTCCCTTATTGCTATATGCCACCTCATCATGC

ACCACCAGTTGGGACTCCTGGGATGATCATGGGTAAGCCTGCAATGGACCCAGCTATGTACGCTCAACAGCCACATCCCT

ATATGGCTCAACAGATGTGGCAACAGGCACCAGAGCAACAGCAATCGCCTTCAGATCATTAGTAGCTGCAGCATAGAAGT

TGAGAAGTGTAAATTTGCTTCTTTTTAATTTGTCGCTGGGTGCTTGTTGAAAACTAGAATGGTGAAGGTGGCCATTTAAA

TGTTTTATAGGAAAAGATCAGTGTAGTTGCTTGACTTTCACTTTTCCTGCCTTCTCTTCTCAAAGTTTGTATCTTCTGTT

TTATATTTAGTTTGTTCTGGTTAAAGTAGGTGATGATGTAGATAACCAAAGCTAATGCTTTACCCACTTATTCTTTGTGG

TATATATGCCTTTTCATTGATTTTAGCATTAGCATCATGGCTGATTCCATGTCCAATTCCTATTCTTTTCATTGATGTTT

TTCTTATGA

>JrNF-YC3|Cluster-14922.50413

GAGAGAGAGAGAGAGAGATTATAAGTATAGTTCCAAAGGCTTTACAGATTTCTTTTGCATTGATTTGTTTTAAGAGTAAT

TCAAGGGGATAACCCCTAGGGGTTGGCTTAAATGGTAAAGATCTTGGTCTTGGAGGTATGATCCCCTCAGGTGTAAGGTT

TAGATCCTTGTGTGCAAACAATTTCTAGAGTCACGTTTCATCGGTGAAAAGCCAATGATTTACTTGGATCCATGTGATGT

GGGCACATAATACGGGTCCAGGGTTTAACCAGATAAAAGACATGTTGCATTTGTACGGTTTCAAGGTTCCTTGTCATTAA

AAAAAAAATGTAATGCATGGCTGGAGTACCAGAGGTTTTGTTCAATAACGATTTGGAGTGTCGACAGCTTATGCTGTTTT

TCTCAAGTTTACATTTTTTGTGTGGCAACTTTGGACAGAAAATTATTAATTGACTTATATCTTTCTAATTCTGTCTTTTC

TGGGTTCTTATTTATACTTAGCATCAGAAGTGGTAGCATAAAGAATTTCCTCCAGGCAAACCTAAGCTAGTATGGATCAG

TTAGGGCATGGACAACCCCCAGCAATTGGGGTGGTTGGTAGTGCAGCTCAAGTGTCATATGCCCTCAATCCATATGAATC

TAACCAAATGATTGGGGCCTCCCAACCGGGATCGGTGGGATCCATGCAGTCTCCTAGTCAGACAGCAGGTCTCTCTGCCT

CTTCAGCTCAGCTTGCACAAAACCAACTTGCTTATCAGCATATCCACCACCAACAGCAACAGCAACTGCAGCAACAACTC

CAAAATTTTTGGGCAACTCAGTATCAAGAGATTGAGCAGGCCTCTGATTTCAAAAACCATAGCCTGCCATTGGCCAGAAT

TAAGAAGATTATGAAGGCCGATGAGGATGTAAGGATGATATCAGCTGAAGCTCCTGTCATATTTGCCAGGGCCTGTGAAA

TGTTCATTCTGGAGTTGACGCTGCGCTCTTGGAATCATACAGAGGAGAACAAAAGAAGGACACTCCAGAAAAATGACATT

GCAGCAGCAATTACAAGGACTGACATATTTGATTTTTTGGTTGATATTGTCCCAAGGGAGGATTTAAAAGATGAAGTGCT

TGCATCCATCCCTAGAGGCAATGCTGCTGTTGGAGGCGATGGTCTTCCCTGCTATTATGTGCCACCTCAGCATGCCCAAC

CGGTTGGTGCTCCAGGGATGATCATGGGCAAGCCTGTAATGGATCAAGCTCTTTACGGCCAACAGATGCGCCCTTACATG

ACTCAGACAATGTGGCCACATCTGCAACAGGAGCAGCCGCCTTCAGATTCTTGAATTTGAGGAATAGGTGACTGATACTT

TGTGTGTCTAATTTGAAAATCGCTTTCGTGCAGTACAAGTTAATTGTATGCTTTTTTTAATATGTAGCTATTACAGCTAA

AAACTAAAAAGTGCTTGTTTTTGGTTATTTGACACATCTTTCCTCTTCGTGTAATAGCACCTTGGATAATTGGATTCCAT

AGATTTTCTCATTTAGTATGTGAAATATGGAAGTTATTCTCGAGCATGACATAATCTATATGCTCTTTACATC

>JrNF-YC4|Cluster-14922.50411

GGCAACTCAGAATCGCAAAAACAGACCTTTTCGTTTCTTTGTTCCTTCCTCTCCTAGGCCTCATTCTTGCAAAGCAGAGG

AACGCCCGTCTGAAAAGGAGGCATAAACTTTTTTGTTATTTAGTTGGATCAGAATGGATCAGCAAGGGCATAGCCAGCCC

ACATCTGTGGGAGTTGTTGGTAGTGGAGCCCAATTGCCATATGCCACAGACCCATATCAGACAAACCAAATGACTGGAGC

ACCGAGTCATGGATCTGTTGTTACATCAGTTGGAGCTATTCAATCTACAAGTCAGCCTGCTGGAGCTCAGCTCCCACAAC

ACCAGCTTGCTTATCAGCACATCCACCAGCAACAACAACAGCAACTTCAGCAACAACTCCAATCTTTTTGGGCAAATCAG

TACCAAGAAATTGAGAAGGCAACTGATTTCAAGAACCATAGCCTTCCCCTAGCAAGGATCAAGAAGATTATGAAGGCCGA

TGAGGATGTAAGGATGATATCAGCTGAAGCTCCTGTCATATTTGCCAGGGCCTGTGAAATGTTCATTCTGGAGTTGACGC

TGCGCTCTTGGAATCATACAGAGGAGAACAAAAGAAGGACACTCCAGAAAAATGACATTGCAGCAGCAATTACAAGGACT

GACATATTTGATTTTTTGGTTGATATTGTCCCAAGGGAGGATTTAAAAGATGAAGTGCTTGCATCCATCCCTAGAGGCAA

TGCTGCTGTTGGAGGCGATGGTCTTCCCTGCTATTATGTGCCACCTCAGCATGCCCAACCGGTTGGTGCTCCAGGGATGA

TCATGGGCAAGCCTGTAATGGATCAAGCTCTTTACGGCCAACAGATGCGCCCTTACATGACTCAGACAATGTGGCCACAT

CTGCAACAGGAGCAGCCGCCTTCAGATTCTTGAATTTGAGGAATAGGTGACTGATACTTTGTGTGTCTAATTTGAAAATC

GCTTTCGTGCAGTACAAGTTAATTGTATGCTTTTTTTAATATGTAGCTATTACAGCTAAAAACTAAAAAGTGCTTGTTTT

TGGTTATTTGACACATCTTTCCTCTTCGTGTAATAGCACCTTGGATAATTGGATTCCATAGATTTTCTCATTTAGTATGT

GAAATATGGAAGTTATTCTCGAGCATGACATAATCTATATGCTCTTTACATC

>JrNF-YC5|Cluster-14922.62047

AGAGAGAGAGAGAGAGAGAGAGTGCGGAGAAGAATCGGGAATCGGGACCTCTCAGAGACAGCAGTACCTAAAGTCAATCT

TCTTCCACTACCTCAACTCCACTTCTCTTTTGGGGTTTCAGCTGCCATTGAGAAGACCCGGAGCCGGTGAAACCTGGACC

AATAAAACGCAGCGACTCAGCATCAGAAGTGGTAGCATAAAGAATTTCCTCCAGGCAAACCTAAGCTAGTATGGATCAGT

TAGGGCATGGACAACCCCCAGCAATTGGGGTGGTTGGTAGTGCAGCTCAAGTGTCATATGCCCTCAATCCATATGAATCT

AACCAAATGATTGGGGCCTCCCAACCGGGATCGGTGGGATCCATGCAGTCTCCTAGTCAGACAGCAGGTCTCTCTGCCTC

TTCAGCTCAGCTTGCACAAAACCAACTTGCTTATCAGCATATCCACCACCAACAGCAACAGCAACTGCAGCAACAACTCC

AAAATTTTTGGGCAACTCAGTATCAAGAGATTGAGCAGGCCTCTGATTTCAAAAACCATAGCCTGCCATTGGCCAGAATT

AAGAAGATTATGAAGGCCGATGAGGATGTAAGGATGATATCAGCTGAAGCTCCTGTCATATTTGCCAGGGCCTGTGAAAT

GTTCATTCTGGAGTTGACGCTGCGCTCTTGGAATCATACAGAGGAGAACAAAAGAAGGACACTCCAGAAAAATGACATTG

CAGCAGCAATTACAAGGACTGACATATTTGATTTTTTGGTTGATATTGTCCCAAGGGAGGATTTAAAAGATGAAGTGCTT

GCATCCATCCCTAGAGGCAATGCTGCTGTTGGAGGCGATGGTCTTCCCTGCTATTATGTGCCACCTCAGCATGCCCAACC

GGTTGGTGCTCCAGGGATGATCATGGGCAAGCCTGTAATGGATCAAGCTCTTTACGGCCAACAGATGCGCCCTTACATGA

CTCAGACAATGTGGCCACATCTGCAACAGGAGCAGCCGCCTTCAGATTCTTGAATTTGAGGAATAGGTGACTGATACTTT

GTGTGTCTAATTTGAAAATCGCTTTCGTGCAGTACAAGTTAATTGTATGCTTTTTTTAATATGTAGCTATTACAGCTAAA

AACTAAAAAGTGCTTGTTTTTGGTTATTTGACACATCTTTCCTCTTCGTGTAATAGCACCTTGGATAATTGGATTCCATA

GATTTTCTCATTTAGTATGTGAAATATGGAAGTTATTCTCGAGCATGACATAATCTATATGCTCTTTACATC

>JrNF-YC6|Cluster-14922.55754

GTCGCTTCAAGGGCATCGAACACACGCACAAAAACATCATCGTCTCCCAACGTTAAAATAAATAAATAAATAAAGAGAAA

AAGGCAGCAGCAAAACTCGGCTTAGCTTAAAGCCCCATCACATATACTCTCTCTCTCTCTCTCAACCCCAAAACTCATCA

ATCTTTGTGGCGGCTTTTCCATTTTCCTATCAGATTGGATCGTTGTCCCCTTTCCCGTCTCTTGGAAGATTTGGATCTGA

GACCGCGTGCGATCTGTGTTTTTTCTTTCTTAAAGAACTCCGCCATGGTTGGGGCCGTACAACAGGAGCAGCAGCAACAG

CAACAGCAACTTCAGATGTTCTGGGCCAACCAAATGCAAGAAATTGAGCAAACAACTGACTTCAAGAATCACAGCCTTCC

TCTTGCTCGGATTAAGAAAATAATGAAAGCTGATGAGGATGTCCGGATGATTTCGGCAGAGGCTCCTGTCATATTTGCAA

AGGCATGTGAAATGTTCATCTTGGAGCTGACTTTGCGCTCTTGGATCCACACAGAAGAAAACAAAAGGAGGACATTACAA

AAGAATGATATTGCAGCTGCCATTTCGAGGACTGATGTCTTTGATTTCTTGGTTGATATCATTCCAAGAGATGAATTGAA

AGAGGAGGGACTTGGGGTGACCAAGGCTACTATTCCAGTAGTGGGTTCACCGGCTGATATCCCATACTACTATGTTCCAT

CACAGCATCCTGTGGGACCTACAGGAATGATCATGGGAAAGCCAGTTGACCAAGCAGCAATGTACGCTTCGCAGCAGCCT

CGAGCACCTATGGCTTTCATGCCATGGCCACAGAGTCAAACTCAGCAGCAGCAGCCACCACAGCAGCAACAAACAGACTC

TTGAATCTTGGTCACTGTGGTCTAGGTAATGTAACAGATGTTTTCAGGCACCCTTTTGACTGTTAGAGATGAATTGCTGG

TAGTGTCTTTTAGCAAGTAGAGTGTTCTTTTTTTCTTCCTAAGTTGGTAGCTTTAATGCTGAGTGGATGATGAGCTTGTG

ATTTGGTACTATGTATGGGCTTTGTAGTCGCCTCTTATTTAAAAGTTTTTCTCAATGTGCATCCTTGGATGTTTTGAAGA

GTACGGGTGAATGGTGATTGGTTGGCTTGAGGAGGAGAGGATACGGAAAGCTTAAATAGTTTTCAAAAATGACCAAAAAT

CTTTACTCATATTCTCATTCAAGAGAATATGAATCTTCTACTCAATTGAGGGCATCCTTGGCTAGCAAGTG

>JrNF-YC7|Cluster-14922.44401

GGCAACTCAGAATCGCAAAAACAGACCTTTTCGTTTCTTTGTTCCTTCCTCTCCTAGGCCTCATTCTTGCAAAGCAGAGG

AACGCCCGTCTGAAAAGGAGGCATAAACTTTTTTGTTATTTAGTTGGATCAGAATGGATCAGCAAGGGCATAGCCAGCCC

CCACCAATGGGAGCAGTTGGTGGTGGATCCCAATTGCCATATGCCATTAACCCATATCAGACGAACCAAATGACCGGGCA

GCTGACTCATCCATCTGTTGGGGCTCAGCCTGCTGGAGTTCAGCTTGCACAACACCAACTTGCTTATCAGCATATCCACC

AGCAACAACAACAGCAACTTCAGCAACAACTCCAATCTTTTTGGGCAAATCAGTACCAAGAAATCGAGAAGGTAACCGAT

TTCAAGAACCATAGCCTTCCTTTAGCAAGGATAAAGAAGATTATGAAAGCTGATGAGGATGTAAGAATGATATCAGCTGA

GGCACCAGTAATATTTGCCAGGGCATGCGAAATGTTCATCTTGGAGTTGACATTGCGATCTTGGAATCACACAGAAGAGA

ACAAAAGGAGGACACTTCAAAAGAATGACATTGCAGCGGCAATCACTAGGACTGATATATTTGATTTCTTAGTTGACATT

GTGCCAAGGGAGGATCTGAAAGATGAAGTACTTGCATCAATCCCTAGAGGAACAATGCCTGTTGGGGGGCATGCTGATGC

TCTCCCTTATTGCTATATGCCACCTCATCATGCACCACCAGTTGGGACTCCTGGGATGATCATGGGTAAGCCTGCAATGG

ACCCAGCTATGTACGCTCAACAGCCACATCCCTATATGGCTCAACAGATGTGGCAACAGGCACCAGAGCAACAGCAATCG

CCTTCAGATCATTAGTAGCTGCAGCATAGAAGTTGAGAAGTGTAAATTTGCTTCTTTTTAATTTGTCGCTGGGTGCTTGT

TGAAAACTAGAATGGTGAAGGTGGCCATTTAAATGTTTTATAGGAAAAGATCAGTGTAGTTGCTTGACTTTCACTTTTCC

TGCCTTCTCTTCTCAAAGTTTGTATCTTCTGTTTTATATTTAGTTTGTTCTGGTTAAAGTAGGTGATGATGTAGATAACC

AAAGCTAATGCTTTACCCACTTATTCTTTGTGGTATATATGCCTTTTCATTGATTTTAGCATTAGCATCATGGCTGATTC

CATGTCCAATTCCTATTCTTTTCATTGATGTTTTTCTTATGA

These are the protein sequences of 33 Walnut NF-Ys，full length

>JrNF-YA1|Cluster-14922.48043

GIGVHTEDDKTHSVS*LAGIMCLPIYLKNK*NVVYP*HNFHLSLKRELCNQRSYIIY*NTGLVLDYHRQIDSPSAVSNTEFIGFQSPSQPLHISGDHYFRSGLGTMHQKQDGTNHQNTVCFQPWWRGIGHSPISADFLGETAASLSPSKNSNDSLGSKTRKLQVEDGLDEGNDVNKKIEITLVSQADGKCDQEQQNAASSKPEAMGQYLTPPTELELLSHSIACASYQVSNPYYGGVMPAYGSQALVHSHCLGGQPTRMALPLEMAEEPVYVNAKQYHGILRRRQSRAKAELEKKLIKFRKPYLHESRHLHAMRRARGSGGRFLNTKKPSGSDANTAPEKGAIGAVSSYTFNPLHSEQSNLSGNMHSSYDHMKVTGIHVPETHQQPTYSNANGNGCYPHHQGFQFSAYHSLSGDRTEVVDCSEKQHERLMVNGAHRALIIK*TFYTTSRYSSSSGVCSFTKKASIDKARLVSGKSFLCSSHVQRK*LL*GFCLKSEQVTFFFFPFVFRV*L*RFATWQYLSFFFFFF

>JrNF-YA2|Cluster-14922.79874

RERERVCVFIYLAHDAKEVA*KLKLIGRGRGIPFPLHLYPFIGCLCVSLLYTRLRLYRHGSYQMGFLCLSGRFKAAAFY*ERFAGSRVNLPNTVNLRNLARTILER*MAAETFYFKQRQGTVHNPIGQLSAPWWSAITSPSVYGEACVQLKPLSMEHPSGGDQLITNNQAARGTEAAARANTTRFTLFPDNCRSSADGQKSQVAISLQSALPEYRGCLELGFGQPMIYAKYPYGDQCYGVFPTCGPQIPGRVMLPLNMTTDDGPIYVNAKQYHGIIRRRQSRAKAVLKNKLTRVRKPYMHESRHLHAMRRPRGCGGRFLNTNILNKRKNGTELNKNGDGQISQATGSQSSEVLQSDSGTLNTSKDANGSGSNISVSEVTSMFSRGDLDYFPINHLMPSVHSLSGKIDGGHGIIMPGKWVAAVDNCCSLKV**QGIGWCWC*MRQSPSLLQPDGEVSLQ*LVLATGFC*GGCPHHVVAKNGNSFLAHVHRGCLLLFLAAFLA*GG*RCLMYFYVL*INGVVDK*VLLAN*IRASMSGYEFVN*RY*SVMLLKFDYLVCLCLTFGVMYPVTSRLFCYLILWKHQALLKNLVFLSSVFEA*CLIPILDLQFEKHFF*RCKGFIIS*SRVLCLGDRMCLE*LSEGVR*YE*VL*FFAVRYKLQYTA

>JrNF-YA3|Cluster-14922.46846

YLCV*VDSKTQDGRHVQVHPNSTIPCFFMSSWIPKLIPKRVERNLLVEGPVAFL*KSEVLTTLRPYEFWTCQETIGIPVHSMPRFTANCPLWWNSDVHRIPPALSENACLKAGSPPQLCHDAKHLGLQLPIQESTTNQLTGQFHPKAGAAGGSNSQDQCISSESDGDEGCVRDVEGQKKPFLLNNPYIICNPSQVAHNYLMVG*YVDYGILFVRYSCKLILLSFQSRVSHAYAEPCLLTTYRLPAIIQPEMVGRVSARVPLPLDFSDDGPIYVNPKQYHGILRRRQSRARLEAKTKVVKTRKPYLHESRHRHALNRVRGSGGRFVSTRKLHNPDPAATDLLSSKTMSEFDSDRFAAGQDTTCSNITLISNINDNNTFQLRDPRLSGISPLMGGSMQCNGGLVSGGTQHCASVVR*DEYKRPRYWSGNSSLALLLYFLYVSYMSCKKLLAYHDVISTKLFHLHVLIREHRYGC*YITFAVPKLFAQVCQAKH*HVLYSHVWHGLERDGGKCLTMVCRIYRRHYALVYIPYTWVFAYSCDQ*NYVY**KKKLWILCSSRISLFLSWLMLWLQFLLSYLFSSLYLTKCLYL**LL*FPIPGCFCYRE

>JrNF-YA4|Cluster-14922.46847

SLSLALSVIYFSSCRGSFIGLES*LISCFCCLLGACGV*NHSDDDDGKIVLVLLIPKRVERNLLVEGPVAFL*KSEVLTTLRPYEFWTCQETIGIPVHSMPRFTANCPLWWNSDVHRIPPALSENACLKAGSPPQLCHDAKHLGLQLPIQESTTNQLTGQFHPKAGAAGGSNSQDQCISSESDGDEGCVRDVEGQKKPFLLNNPYIICNPSQVAHNYLMSRVSHAYAEPCLLTTYRLPAIIQPEMVGRVSARVPLPLDFSDDGPIYVNPKQYHGILRRRQSRARLEAKTKVVKTRKPYLHESRHRHALNRVRGSGGRFVSTRKLHNPDPAATDLLSSKTMSEFDSDRFAAGQDTTCSNITLISNINDNNTFQLRDPRLSGISPLMGGSMQCNGGLVSGGTQHCASVVR*DEYKRPRYWSGNSSLALLLYFLYVSYMSCKKLLAYHDVISTKLFHLHVLIREHRYGC*YITFAVPKLFAQVCQAKH*HVLYSHVWHGLERDGGKCLTMVCRIYRRHYALVYIPYTWVFAYSCDQ*NYVY**KKKLWILCSSRISLFLSWLMLWLQFLLSYLFSSLYLTKCLYL**LL*FPIPGCFCYRE

>JrNF-YA5|Cluster-14922.50230

FLFLFFFLMYSNLDVQIVSINTEGKQSCLKVTCNLIIYIFCILELVL*FRNSSHNT*KKAKVFEMFLIVISNLWHGGLLLENAMVIR*SMIRNCVLDLWS*GMLVYCTRLYVYS**CN*QNRFTSVVKSNFYCHFLFPIGIEFP*F**FNS*RMHI*FVYVQACGCARTHTHTSGCN*KQV*VFLFYIIKNPSLFLIILKFSKQ*FEPMQNLCKKESVISFPYSASPYFLGCPSWENSAESHVEQSSTSRILSMKMGVSPQHFHNTKQLNFQFQDQDSSSTQSTCESYPEVVSMGERNPCVQGIVSPQSGFIETKGKPVGGIIKSGSPIGTRDFVFSPSQLDYSQSIARIPFHYADPYFGGLLASAYGQQSIIHPSQMIGVAPTRVPLPLDLIDDEPIYVNAKQYHAILRRRQYRARLEAQNKVIKDRKPYLHESRHLHALKRARGSGGRFVNTKKLQESKQTPMSNGLDVSGFAQPLLTRDMAESEVHLPENYRDRAPTTSCSDVTSASNSDDIFQQPEFRFSGYPSSFGRTMQVHLVDMHDGGGGGKHHLR*EMLPTTLIQQFVFHFWRR*LLEF*REVILGSFAIYNFTSLSP*LVKNQV*TVTGYTLFWHLGIN*HLKTTTCQVGDH*QYRNCSLCS*YAFVCGNSLPTFSSL*WQKFLLKAA*KIKKLS

>JrNF-YA6|Cluster-14922.72325

FFFFFKKKFLV*IYWGSGDWD*SDWVGNGHFLFIPADMVTN*RLSFLTILSLPEALDR*ICLHGWFSLSLYLSANLWLA*QQRESICKVFLVSGVP*FLFHFY*GAIFHYAYAVTTVIMIIEIILCARRLFAFSQLM*FSFWWSYKLHRNKTLVLHETNISHD*FARIWNGLTCICHGQGYPPFLGMPHARMPLPLEMAQEPVYVNAKQYQGILRRRQARAKAELEKKLIKVRKPYLHESRHQHAMRRARASGGRFAKKSNVDAANHEAKEKGIASGPALSSQSASSSGSEPLLTDSAEIWNSSHGQQEGRQDASEAQNYVNGDNRYQNHEVLPVSSYLHMGGRGEEGDCSGQQWGSISSNQASQRRLAIQ*TPVGRELGARIVDWTPP*WNGISPCESVTDSILGNLYARRQIILGFNIVHVTWMKSGEVERLLKRFPVLYFSLFFHFCSLYKGRD*LADSKFAF*TLGLMYKPSC*LDRRLDDLSLCCLC*SFHRI*FELCGTLSFSNIDFD*LVDEIVDPTIFCLSKPT

>JrNF-YA7|Cluster-14922.32667

RERERVCVFIYLAHDAKEVA*KLKLIGRGRGIPFPLHLYPFIGCLCVSLLYTRLRLYRHGSYQMGFLCLSGRFKAAAFY*ERFAGSRVNLPNTVNLRNLARTILER*MAAETFYFKQRQGTVHNPIGQLSAPWWSAITSPSVYGEACVQLKPLSMEHPSGGDQLITNNQAARGTEAAARANTTRFTLFPDNCRSSADGQKSQVAISLQSALPEYRGCLELGFGQPMIYAKYPYGDQCYGVFPSCGPQISGRVMLPLNMSTDDGPIYVNAKQYHGIIRRRQSRAKAVLENKLTRVRKPYMHESRHLHAMRRPRGCGGRFLNTKNSNKGMSGTKVNKAGDGLISQPTGSASSEVLQSDSGGALNSSRDANRSVSNISVSEVTSMFSRRDLDHFPINHLIPSVHSLSDMISGVRGIVMPSKWVAAPNCCNLKV*QQVIKWSWC*LHQSPSLLQPDGEVSLQ*LVLATGFC*GGCPHHVVAKNGNSFLAHVHRGCLLLFLAAFLA*GG*RSLM*MYCRLNGVGDK*VCFTN*IRASKSG*VLKCDV*V*YLVRLCLTFGLMYCRTV*LCCYLMPPCFSQEHGIRVVIHI*STIMHYAILDL*F*ETLSKDAKN*T

>JrNF-YA8|Cluster-14922.32809

RERERERLQSR*ASTCERPKRLEFRSAASRKL*MMPAKPGNEDGRLEHGGQTVLQSATYSQPWWRGVGSNVSLGDNASKSTSMEHLNVSVGNGAMQLKVNGPLDDGAKFNKETRDGNIGKEHRDNNRVPSLTPKMVEHLDPNSQMELVGHSIVLTSYPYSDQQYGGMFASYGPQAMIPQLYGMHHARMPLPLEMEEEPVYVNAKQYHGILRRRQSRAKAELEKKVIKVRKPYLHESRHLHAMRRARGCGGRFLNTKKLDNLSDPPSEEGMNMGAKPSMGPTISSGSKCFFNNGNGNSGSSLNQHKGSLPIVHNTHGAHCFSKGNSSSNGLSSTYHSLFSDGKEVDCLGQQRESMQVNGVAHGALPIK*HSLKVNQKVFAASSFGRNPRVSQAIHSWLKKNKFTCLNSR*ELGDKYDSLPLWSEVHTFFFLSLLV*NAQSS*LIK*EMVMYV*NFNNLGMEVSEV*CLFMTRVC*PDGVMLTML*AYLSLSAFIAI*RVLDDNKDAKHR*EVHPINL*LNGGLVAYNSSPSSHRSVEIRGWEEIKKAW

>JrNF-YA9|Cluster-14922.60069

SGKIGGYRWWGCVFLLYIADLKSLCSVVWLQFK*NKSSRSRKSETCTDVENKQGKSFQFWIGRERDCLYGFQDSRGETCPSSYQQHYSSLLLHVLVDPKE*LMLNEWKGTCLLLRIL*ILHQIQKTDNFDPTHILKLPRKQLKCNFVHSMTHSAVPFPSWWNPDEQKFLSSLSKNASMKAGSAPRLCHDAKHLGLQLADQESSTIQLTGQSRHKVDIMGGSNCQDQCVSSESDGDGSCVKRIVEGQMKPVYLLNNPDIIINPSQISHHHSMVG*SFEILFVRYAFKLMSLLSFQARVPYPYAEPCFSGLLTNYGQQTIIQPQMVGIAPARVPLPLDLMDDGPIFVNPKQYHGILRRRQSRAKLEAQNKVVKTRKPYLHESRHRHALNRVRGSGGRFVSVRKLQNPDSSATSNTHCSSDCVDLHPSKKMSEFHSHQFQDVASLTTCSDIKHVSNSNNGNGSFRRFSRPMQCNCNGGLVSGGTQHSASVVQ*DMGNKQDR*SVSSVRQFILGSVTLLSMFPACLPKCATFATSY*DLY*FRLHGVMKDIDMVL*EDCAFNSTLLIHHLCCCYQFCARIGYYQREFEVSEYGSFLDSVWPSLKLNLAQVVFSGFKTVFQPIWCLSSLVRVVMTCTELDPIRVPITVSITSHFRLCADAEVILYDST

>JrNF-YA10|Cluster-14922.95251

GGGCPYYLC*NS*SATNILLAFCSLPHD*MFVKWVYEGLFHFICFLTLFIFIFILPLLLLFSTFLVLMITF**ESFSSPVYQMLIFNDVCTLKV*RHG*HLTKQGYFNVIHFSNEMGRNCDICWILYYFFYK*KRIAGCYIIYLLRFQTYCS*YAVDVMSCFKLWIIPRGMIDYRLIADSSMTCKIASFLVCHCMKMCFLCDSMRYFLLKCPQIHHPQIIGMTPTRVPLPLDLTEDEPIYVNSKQYHAILRRRQYRAKLEAQNKLIKDRKPYLHESRHLHALKRARGSGGRFLNTKKLQEDASIASCSDVISASNSDNILQPPEFRFNCYPSPVGGTMRGHSVDMHGGGGGGKQHLHLPSTLQ*EMWQTTHIQQSLFHFWRWLLEGKSSLAILLFSVLLLVT*ACQKTKHKPLLAMYHFRIGAYKHLKTNVIMLSR*LLILDSNIFAPLSKT*WEKK*TDYGSLLFHYSWET

>JrNF-YA11|Cluster-14922.46822

LLPKASANFTHAGLEPSCALDFQPVTSTHRLSSIPTLVANRRRNGKPDR*VPGHYSSTRRNYKQF*PTWGRVGDAIRALVLPLQCERGRVHSN*KLD*PLSDSLRDQNSRV*LYQE*RHLHFYKEFEGMQQKSEKPNRLDPRSHAIQPPAVYTEPWWRNIGYNPISPAVTGGNVSNSSSLECPDTGSDSNDGQSLSNNEPNEEGDDATKESQNTASSRSAGNYGQHHQNMQHVASTAPSMRNECLTQPAQLELVGHSIACASNPYQDPYHGGMLAAYGHQPYGYPPFLGMPHARMPLPLEMAQEPVYVNAKQYQGILRRRQARAKAELEKKLIKVRKPYLHESRHQHAMRRARASGGRFAKKSNVDAANHEAKEKGIASGPALSSQSASSSGSEPLLTDSAEIWNSSHGQQEGRQDASEAQNYVNGDNRYQNHEVLPVSSYLHMGGRGEEGDCSGQQWGSISSNQASQRRLAIQ*TPVGRELGARIVDWTPP*WNGISPCESVTDSILGNLYARRQIILGFNIVHVTWMKSGEVERLLKRFPVLYFSLFFHFCSLYKGRD*LADSKFAF*TLGLMYKPSC*LDRRLDDLSLCCLC*SFHRI*FELCGTLSFSNIDFD*LVDEIVDPTIFCLSKPT

>JrNF-YA12|Cluster-14922.82902

RERERERLQSR*ASTCERPKRLEFRSAASRKL*MMPAKPGNEDGRLEHGGQTVLQSATYSQPWWRGVGSNVSLGDNASKSTSMEHLNVSVGNGAMQLKVNGPLDDGAKFNKETRGTVASKSDGNIGKEHRDNNRVPSLTPKMVEHLDPNSQMELVGHSIVLTSYPYSDQQYGGMFASYGPQAMIPQLYGMHHARMPLPLEMEEEPVYVNAKQYHGILRRRQSRAKAELEKKVIKVRKPYLHESRHLHAMRRARGCGGRFLNTKKLDNLSDPPSEEGMNMGAKPSMGPTISSGSKCFFNNGNGNSGSSLNQHKGSLPIVHNTHGAHCFSKGNSSSNGLSSTYHSLFSDGKEVDCLGQQRESMQVNGVAHGALPIK*HSLKVGCLLK*FTSSVLMTLEFSCNRERSNFVSYERLLCR*TRRCSPHPALGETLVFLRQFILGLKKTSLLVLILGENWETNMTRCHYGLKFIHFSSFPS*CKMLKVPNLSSRKWLCMCKISTI*VWRFQKCDACL*QEFASLMVLC*QCYELISLSLLLLQFDEC*MTIKMQSTDRRCILSIYD*MEVWLPITAALHPIGRLR*GVGRKLRKPG

>JrNF-YA13|Cluster-14922.52088

LLPKASANFTHAGLEPSCALDFQPVTSTHRLSSIPTLVANRRRNGKPDR*VPGHYSSTRRNYKQF*PTWGRVGDAIRALVLPLQCERGRVHSN*KLD*PLSDSLRDQNSRV*LYQE*RHLHFYKEFEGMQQKSEKPNRLDPRSHAIQPPAVYTEPWWRNIGYNPISPAVTGGNVSNSSSLECPDTGSDSNDGQSLSNNEPNEEGDDATKESQNTTYSRSAGNYGQEHQNVQHVTSTAPSMRDECLTQPAQLELVGHSIACASNPYQDPYYGGMMAAYGHQPYGYPPFLGMPHARMPLPLEMAQEPVYVNAKQYQGILRRRQARAKAELEKKLIKVRKPYLHESRHQHAMRRARGSGGRFAKKGDVDSSNHDAKEKGIGSGPALSSQSASSSGSEPLLTDSAETWNSSHGQQGGRHDASEAQNYVNGGDHYQNHNGLKASSYLHAGERGEEGDCSGQQWGSISSKQSSQRRLAIQ*IPAEVGCQYGGLEPHPDGTGCISCISVDSILGNLYARRQIILGFYIMHVTWMKGWRRDDEWAMLWLLKRFPVFHPILFFHMAVCTELEIRRLKVCLLNYGLM*KPSCLIGGLIIFVYF*VNVAHRI*IVAYLAKL*RLRNI*FSSTSA*PNGCNC*PSRNACYVPSLHAYTSFRDTFAQTPSYNLYS

>JrNF-YA14|Cluster-14922.84458

LLLSTKRGRGSALILLFYS*EFVLAKPYRFWVLQEYPAHDFVPLLSLWFLCFTTPTYGSCWRNVSVL*SGFYFLSCLSI*FLFFIKWVLCIFFP*NFGWRLWKSCAFFFFFLKYRSSSYSYGFELIYGYF*QNLLIFLSF*FPLY*CSCGLYTKLRNGPDCIMVYLISSRSLVYIEDKL*SINYLFIIFHILFNLYLTRLAGNFGCNLNCRFAGSRVNLPNTVNLRNLARTILER*MAAETFYFKQRQGTVHNPIGQLSAPWWSAITSPSVYGEACVQLKPLSMEHPSGGDQLITNNQAARGTEAAARANTTRFTLFPDNCRSSADGQKSQVAISLQSALPEYRGCLELGFGQPMIYAKYPYGDQCYGVFPTCGPQIPGRVMLPLNMTTDDGPIYVNAKQYHGIIRRRQSRAKAVLKNKLTRVRKPYMHESRHLHAMRRPRGCGGRFLNTNILNKRKNGTELNKNGDGQISQATGSQSSEVLQSDSGTLNTSKDANGSGSNISVSEVTSMFSRGDLDYFPINHLMPSVHSLSGKIDGGHGIIMPGKWVAAVDNCCSLKV**QGIGWCWC*MRQSPSLLQPDGEVSLQ*LVLATGFC*GGCPHHVVAKNGNSFLAHVHRGCLLLFLAAFLA*GG*RCLMYFYVL*INGVVDK*VLLAN*IRASMSGYEFVN*RY*SVMLLKFDYLVCLCLTFGVMYPVTSRLFCYLILWKHQALLKNLVFLSSVFEA*CLIPILDLQFEKHFF*RCKGFIIS*SRVLCLGDRMCLE*LSEGVR*YE*VL*FFAVRYKLQYTA

>JrNF-YA15|Cluster-14922.40699

YLCV*VDSKTQDGRHVQVHPNSTIPCFFMSSWIPKLIPKRVERNLLVEGPVAFL*KSEVLTTLRPYEFWTCQETIGIPVHSMPRFTANCPLWWNSDVHRIPPALSENACLKAGSPPQLCHDAKHLGLQLPIQESTTNQLTGQFHPKAGAAGGSNSQDQCISSESDGDEGCVRDVEGQKKPFLLNNPYIICNPSQVAHNYLMSRVSHAYAEPCLLTTYRLPAIIQPEMVGRVSARVPLPLDFSDDGPIYVNPKQYHGILRRRQSRARLEAKTKVVKTRKPYLHESRHRHALNRVRGSGGRFVSTRKLHNPDPAATDLLSSKTMSEFDSDRFAAGQDTTCSNITLISNINDNNTFQLRDPRLSGISPLMGGSMQCNGGLVSGGTQHCASVVR*DEYKRPRYWSGNSSLALLLYFLYVSYMSCKKLLAYHDVISTKLFHLHVLIREHRYGC*YITFAVPKLFAQVCQAKH*HVLYSHVWHGLERDGGKCLTMVCRIYRRHYALVYIPYTWVFAYSCDQ*NYVY**KKKLWILCSSRISLFLSWLMLWLQFLLSYLFSSLYLTKCLYL**LL*FPIPGCFCYRE

>JrNF-YA16|Cluster-14922.20995

NSKHEDGSFTATFS*HQAIEFSIPRSGFVLNSINW*ILSCSG*YAGKLCLWTRNSFTTIRMY*N*GEACWRYHQICLTNWCSGFFLLAFTE*LQPISCKLMPLFR*L*GEMWISTCSMFPVSGSHSIPPC*SIFWWFIVCCLWFTIYL*RHG*HLTKQGYFNVIHFSNEMGRNCDICWILYYFFYK*KRIAGCYIIYLLRFQTYCS*YAVDVMSCFKLWIIPRGMIDYRLIADSSMTCKIASFLVCHCMKMCFLCDSMRYFLLKCPQIHHPQIIGMTPTRVPLPLDLTEDEPIYVNSKQYHAILRRRQYRAKLEAQNKLIKDRKPYLHESRHLHALKRARGSGGRFLNTKKLQEDASIASCSDVISASNSDNILQPPEFRFNCYPSPVGGTMRGHSVDMHGGGGGGKQHLHLPSTLQ*EMWQTTHIQQSLFHFWRWLLEGKSSLAILLFSVLLLVT*ACQKTKHKPLLAMYHFRIGAYKHLKTNVIMLSR*LLILDSNIFAPLSKT*WEKK*TDYGSLLFHYSWET

>JrNF-YA17|Cluster-14922.27809

VSEIGQDVDHL*PRPSIADQS*FHFQLPLFPMTAIFSFVTRVFHIWNKGFRLLSIHGLPMFKLCFLFHFQLPLFSVTVIFSFVTRVFHIWNKGFRLLSIHGLPMFKLCFLFHFQLPLFSVTVIFSFVTRVVHKWNGFRTNKKKCLNCAFGPNAKFKQLFSILFLFYGPLLGPSQTLCHCFFYVNLL*NTARKIC*LTPPGIPE*GENIDLHIHVI*DTRTSQMQCCAKVWMECFLYPRIKILTGRCSLLYEHVCHKCRVSG*YSNLFFISDGNIGKEHRDNNRVPSLTPKMVEHLDPNSQMELVGHSIVLTSYPYSDQQYGGMFASYGPQAMIPQLYGMHHARMPLPLEMEEEPVYVNAKQYHGILRRRQSRAKAELEKKVIKVRKPYLHESRHLHAMRRARGCGGRFLNTKKLDNLSDPPSEEGMNMGAKPSMGPTISSGSKCFFNNGNGNSGSSLNQHKGSLPIVHNTHGAHCFSKGNSSSNGLSSTYHSLFSDGKEVDCLGQQRESMQVNGVAHGALPIK*HSLKVNQKVFAASSFGRNPRVSQAIHSWLKKNKFTCLNSR*ELGDKYDSLPLWSEVHTFFFLSLLV*NAQSS*LIK*EMVMYV*NFNNLGMEVSEV*CLFMTRVC*PDGVMLTML*AYLSLSAFIAI*RVLDDNKDAKHR*EVHPINL*LNGGLVAYNSSPSSHRSVEIRGWEEIKKAW

>JrNF-YB1|Cluster-14922.21265

LHTCFAYR*TCTHIHASISTSI*QPENRSSKATCLCLRGPN*A*SPHYRDRYMADNRGNNLSIREGFKYHFDGASSTASGEDAPMKEQDQLQLPIANVGRIMKQILPPNAKISKEAKETVQECVSEFISFVTGEASDKCHREKRKTVNGDDICCAMATLGFDDYGEPLRRYLEKYRELEEERAQQGKFCNSTEENNMKHGNNL*YSYIYL*TF*C*S*YLRHFVMFT**VGGGGGEG*F*VLLVCKLIIDYGSSIDHCS*LRYYMKLIKFLCPFFWINYEVNL*HEA

>JrNF-YB2|Cluster-14922.29372

RVLARMADAPTSPNGGGSLESGEQSPRSNVREQDRFLPIANISRIMKKALPANGKIAKDAKETVQECVSEFISFITSEASDKCQREKRKTINGDDLLWAMATLGFEDYIDPLKIYLSRYREVIF*I*LFYVHLSEFLVAAKKKRENEKKEKTWVSFVLQL*HPPGVFFFFFL

>JrNF-YB3|Cluster-14922.32115

YPSVPSPRFPSHFSFSISHARTHRGTP*NSPPRTPSLSRVLVPPRGLGFLVLTDDVVL*LVLCTRVLARMADAPTSPNGGGSLESGEQSPRSNVREQDRFLPIANISRIMKKALPANGKIAKDAKETVQECVSEFISFITSEASDKCQREKRKTINGDDLLWAMATLGFEDYIDPLKIYLSRYREIEGDTKGPAKGGDASSKKNVQLSPNVQVEIGHQGSFSQGMNYSSSQVAQHLMVPMQGTE*ASG*SSSNFNIWYPTLGISCYQNIMKHYSYV*H*HFRVILNSSPEVWQVTSYIYVS*NTMTI*TEYL*MFFLFMSFLDPFF*RCYGNDYETLMP

>JrNF-YB4|Cluster-14922.39672

*CCNFSLH*KNYSIFLCFKAI*LVSSLSSAHLLISFFSGNFIQLQSLELSRLKTVDSSSVLFVLLFFLDVGDSSSVSFGAVSKFAF*NTYRLRFLNIFLSEETEFASSRLICLTSYKF*RSKVLLILLQIKSSFIS*KINLRSYHYGHFLSLKGRERESEEISTSSYLSPYCSNKNLQV*MADSDNESEGGGERAGNASANELSPREQDRFLPIANVSRIMKKALPANAKISKDAKETVQECVSEFISFITGEASDKCQREKRKTINGDDLLWAMTTLGFEDYVEPLKVYLQRFREMEGEKSVAVRDKDASAGGASSTGFGNGGGFEQPGGGGGVYGSSSGMGMMMHQGGHVYGSSGFHQQIGTTGSGFGGLGKSGTGYVGPGSNTGRPR*LFSLIVNVGPLSYLSNILNIYPSEGNSTVIELFMNLYVFFLKINFTHQSQFIFIFYIYIYFSNSV*ECFL*SIR**IVTDEKNFSFL*S*ILLDFCFCKYGVEIEFKVTRGCM*SVDKYSSGFF*FSYIVKTKGYGLMIVIVNHKV*DVISMRICAK*YSFLFLICGFLS

>JrNF-YB5|Cluster-14922.54864

TKENQPKRTVLINLSLISVVSFLFPSPASDLKPSRPSLALGPKLRVLARMADAPTSPNGGGSLESGEQSPRSNVREQDRFLPIANISRIMKKALPANGKIAKDAKETVQECVSEFISFITSEASDKCQREKRKTINGDDLLWAMATLGFEDYIDPLKIYLSRYREIEGDTKGPAKGGDASSKKNVQLSPNVQVEIGHQGSFSQGMNYSSSQVAQHLMVPMQGTE*ASG*SSSNFNIWYPTLGISCYQNIMKHYSYV*H*HFRVILNSSPEVWQVTSYIYVS*NTMTI*TEYL*MFFLFMSFLDPFF*RCYGNDYETLMP

>JrNF-YB6|Cluster-14922.57314

*RSTCCARCNYYPNDPTDPNTSPPPALQLPPTFPELLLSRPFSLSCHSPFQPIRSKSLPLEPYYPIPCIKNFSLPFGPISFEFVILFLCFLLLNLLSLEKLLTWFFSAESFCILVHNLYT*VTSARALNLFQNSVYIF*VRSFGFGLFRFCRTREFITVIWGIFSISI*FHSLKNFRRGSFFSLKLS*NYMTFLLKIC*IGSISCKFIRDPILDG*KLVNLIHFVELHQTQKSFKLKVVELGLVCEAQPDIYKAKRSMADSDNESGGHNNSNSANGDFASREQDRFLPIANVSRIMKKALPANAKISKDAKETVQECVSEFISFITGEASDKCQREKRKTINGDDLLWAMTTLGFEEYVEPLKIYLQKYREMEGEKSTVGSRPGEKDGGGGSGGGGAAAGGGGSGGAVNSASSGGAGGFNGVGGVYGGMYGGVMGGHHHGHMYGSGGFHHVGGIGGGVVGKDGSGGGGGVGGSIVRSR*MCFHCLKKKGGYIK*LR*FRGI*FALNSYR*L*PKTL*SLEIR*SYLDRGWTLLGFSLIFFLWTFWWGCGTCLWNCMCNME*YMCNCGSEDT*YDAWY*KL*PLMIKQ*L*GKRKLHGKYFVVVYGFIHL*SCLLSVLFLKRVWINDLEVSYVYSFMAY*KFGNALN*QFYFEVSTASFFQKGRVCMHACMHAYFFWGTRHVSRVSAICLDGYVSTK*GAL*IGFYMS*NLSLLTIYFYFMFFVRWEDCKLVSTAVIHSLSCMRIHTFMEWKDFGKSDFVVLIFFWFLKNNRITCM*S*ETYKQNKTKQ

>JrNF-YB7|Cluster-14922.58354

*T**AL*IRKRKCKWETATNGGDKLKAKRLALSDPTLFLSFLTLFLSLILLQRPITLSIKKQFHQSSLSLSLPNARQIQN*QNRDVSASFSSSLHSDLGFLGADMAEAPASPAGGSHESGGDQSPRSAGVREQDRYLPIANISRIMKKALPANGKIAKDAKDTMQECVSEFISFITSEASDKCQKEKRKTINGDDLLWAMATLGFEDYIEPLKVYLARYRELEVSKPTLFP*YKFT*FS*L*VSANFEYLETVWFLLF*AVLCFKSSKEIKRQR*KMVAPKGFQRVIFE*PACTEH*HDVFFFYHFWKSKTSEDIIFVSFVYHMIKREYFFNQLFSRTHDQTGL*FFPQYLSSNIRNYIVGMSSIRVIPKDLLGVEMHLVKGMRLVLWPLKTHSLLIRGH*TT*TPNLLMLKVRTISRSSLPDSCQGRSNIGYMDDIWVFILCKETSRKF*LYVNCLCGLYDGLNYMVSAPIA*FMWFL*ILFLAKILYVGRLFWLLSRIEGRPF*DWSRHLNKIL*SDEPCKFALSYSCLTLLAAGICGDLVRGPTLIFGFG*LVIDEVFCMSCLDPKEEFGIWRFSLPLSLS

>JrNF-YB8|Cluster-14922.64236

VMESCGNFS*TSIP*LQLLLLTNLASYLQYPTYNSHSFPYPTALFDPKPYIYSQYRTCLSLHALFLLFFCEAHEIDPDKSQAREF*HLLGLPGLLYT*YCQFNWLCGRASSLMADSEDESGGGQNNANFNGEYSSLREQDRLLPIANVSRIMKKALPANGKISKEAKETVQECVSEFISFITGEASDKCQREKRKTINGDDLLWAMTTLGFEEYVEPLKIYLQKFREMEGEKSSSTTSTAVAGGRQGERDIGGDGSVGNSGNSGVGMMYGGVQSTMMMMGHNYHGGHDMYGSSVSGSGSGASSGRQR*VQLFQG*CFFFFFGYACMIEG***FQFDNFVECLLS**VWNIMDDYIYNILIYWYAMVDVD*RE

>JrNF-YB9|Cluster-14922.72337

LEVITPKRV*EIRNRRSMEDESHGNELDGGGSPESPCLKRSTSNINMKEQDRFLPIANVGRIMKKVIPANGKISKDAKETVQECVSEFISFVTGEASDKCQREKRKTINGDDIIWAITTLGFEDYVSPLKTYLQKYREIEGEKLSNIPKQQRQQQQHKQEQNIPYDDDTHVYGLDKNDNRTVYSSISDLMSQPPFVATDQPFSLHFSPSSVQKQLLPQDQINDHWGIGN*LA*R

>JrNF-YC1|Cluster-14922.36868

QPLKSPPHIPSS*FIPQIPPTHHHPRKTHALAHAESPIWQHFSQTHHQAPFHQTHKSPPPTHQSMENNNQAAQSSSYPPQPTPPPAAPFHHLLQQQQQQLQMFWSYQRQEIEQVNDFKNHQLPLARIKKIMKADEDVRMISAEAPILFAKACELFILELTIRSWLHAEENKRRTLQKNDIAAAITRTDIFDFLVDIVPRDEIKDEAAGLGGIVGPPASGVPYYYPPMGQPAGGPHGGMMIGRPAGAMDPSGVYVQPPSNAWQSVWQGHATDDGSYGSGGSTGQGNLDGQS*SCLFEDVLWRSCSM*LKSNLFRRCGLWRCGKVSGCS*CYLEFLIASEL*DALVVSCYSLWTPIETCYNFNLIRCS*TNMLL*QLF*YYYCAVFSLRILVLLI*MIMAVS*

>JrNF-YC2|Cluster-14922.71538

RERERESAEKNRESGPLRDSST*SQSSSTTSTPLLFWGFSCH*EDPEPVKPGPIKRSDSASEVVA*RISSRQT*ASMDQLGHGQPPAIGVVGSAAQVSYALNPYESNQMIGASQPGSVGSMQSPSQTAGLSASSAQLAQNQLAYQHIHHQQQQQLQQQLQNFWANQYQEIEKVTDFKNHSLPLARIKKIMKADEDVRMISAEAPVIFARACEMFILELTLRSWNHTEENKRRTLQKNDIAAAITRTDIFDFLVDIVPREDLKDEVLASIPRGTIPVGGHADALPYCYMPPHHAPPVGTPGMIMGKPAMDPAMYAQQPHPYMAQQMWQQAPEQQQSPSDH**LQHRS*EV*ICFFLICRWVLVEN*NGEGGHLNVL*EKISVVA*LSLFLPSLLKVCIFCFIFSLFWLK*VMM*ITKANALPTYSLWYICLFIDFSISIMADSMSNSYSFH*CFSYG

>JrNF-YC3|Cluster-14922.50413

EREREIISIVPKALQISFALICFKSNSRG*PLGVGLNGKDLGLGGMIPSGVRFRSLCANNF*SHVSSVKSQ*FTWIHVMWAHNTGPGFNQIKDMLHLYGFKVPCH*KKNVMHGWSTRGFVQ*RFGVSTAYAVFLKFTFFVWQLWTENY*LTYIFLILSFLGSYLYLASEVVA*RISSRQT*ASMDQLGHGQPPAIGVVGSAAQVSYALNPYESNQMIGASQPGSVGSMQSPSQTAGLSASSAQLAQNQLAYQHIHHQQQQQLQQQLQNFWATQYQEIEQASDFKNHSLPLARIKKIMKADEDVRMISAEAPVIFARACEMFILELTLRSWNHTEENKRRTLQKNDIAAAITRTDIFDFLVDIVPREDLKDEVLASIPRGNAAVGGDGLPCYYVPPQHAQPVGAPGMIMGKPVMDQALYGQQMRPYMTQTMWPHLQQEQPPSDS*I*GIGD*YFVCLI*KSLSCSTS*LYAFFNM*LLQLKTKKCLFLVI*HIFPLRVIAPWIIGFHRFSHLVCEIWKLFSSMT*SICSLH

>JrNF-YC4|Cluster-14922.50411

ATQNRKNRPFRFFVPSSPRPHSCKAEERPSEKEA*TFLLFSWIRMDQQGHSQPTSVGVVGSGAQLPYATDPYQTNQMTGAPSHGSVVTSVGAIQSTSQPAGAQLPQHQLAYQHIHQQQQQQLQQQLQSFWANQYQEIEKATDFKNHSLPLARIKKIMKADEDVRMISAEAPVIFARACEMFILELTLRSWNHTEENKRRTLQKNDIAAAITRTDIFDFLVDIVPREDLKDEVLASIPRGNAAVGGDGLPCYYVPPQHAQPVGAPGMIMGKPVMDQALYGQQMRPYMTQTMWPHLQQEQPPSDS*I*GIGD*YFVCLI*KSLSCSTS*LYAFFNM*LLQLKTKKCLFLVI*HIFPLRVIAPWIIGFHRFSHLVCEIWKLFSSMT*SICSLH

>JrNF-YC5|Cluster-14922.62047

RERERESAEKNRESGPLRDSST*SQSSSTTSTPLLFWGFSCH*EDPEPVKPGPIKRSDSASEVVA*RISSRQT*ASMDQLGHGQPPAIGVVGSAAQVSYALNPYESNQMIGASQPGSVGSMQSPSQTAGLSASSAQLAQNQLAYQHIHH7QQQQQLQQQLQNFWATQYQEIEQASDFKNHSLPLARIKKIMKADEDVRMISAEAPVIFARACEMFILELTLRSWNHTEENKRRTLQKNDIAAAITRTDIFDFLVDIVPREDLKDEVLASIPRGNAAVGGDGLPCYYVPPQHAQPVGAPGMIMGKPVMDQALYGQQMRPYMTQTMWPHLQQEQPPSDS*I*GIGD*YFVCLI*KSLSCSTS*LYAFFNM*LLQLKTKKCLFLVI*HIFPLRVIAPWIIGFHRFSHLVCEIWKLFSSMT*SICSLH

>JrNF-YC6|Cluster-14922.55754

RFKGIEHTHKNIIVSQR*NK*INKEKKAAAKLGLA*SPITYTLSLSLNPKTHQSLWRLFHFPIRLDRCPLSRLLEDLDLRPRAICVFSFLKNSAMVGAVQQEQQQQQQQLQMFWANQMQEIEQTTDFKNHSLPLARIKKIMKADEDVRMISAEAPVIFAKACEMFILELTLRSWIHTEENKRRTLQKNDIAAAISRTDVFDFLVDIIPRDELKEEGLGVTKATIPVVGSPADIPYYYVPSQHPVGPTGMIMGKPVDQAAMYASQQPRAPMAFMPWPQSQTQQQQPPQQQQTDS*ILVTVV*VM*QMFSGTLLTVRDELLVVSFSK*SVLFFFLSW*L*C*VDDELVIWYYVWAL*SPLI*KFFSMCILGCFEEYG*MVIGWLEEERIRKA*IVFKNDQKSLLIFSFKRI*IFYSIEGILG*QV

>JrNF-YC7|Cluster-14922.44401

ATQNRKNRPFRFFVPSSPRPHSCKAEERPSEKEA*TFLLFSWIRMDQQGHSQPPPMGAVGGGSQLPYAINPYQTNQMTGQLTHPSVGAQPAGVQLAQHQLAYQHIHQQQQQQLQQQLQSFWANQYQEIEKVTDFKNHSLPLARIKKIMKADEDVRMISAEAPVIFARACEMFILELTLRSWNHTEENKRRTLQKNDIAAAITRTDIFDFLVDIVPREDLKDEVLASIPRGTMPVGGHADALPYCYMPPHHAPPVGTPGMIMGKPAMDPAMYAQQPHPYMAQQMWQQAPEQQQSPSDH**LQHRS*EV*ICFFLICRWVLVEN*NGEGGHLNVL*EKISVVA*LSLFLPSLLKVCIFCFIFSLFWLK*VMM*ITKANALPTYSLWYICLFIDFSISIMADSMSNSYSFH*CFSYG

These are the protein sequences of 33 Walnut NF-Ys, conserved regions only

>JrNF-YA1

PVYVNAKQYHGILRRRQSRAKAELEKKLIKFRKPYLHESRHLHAMRRARGSGGRF

>JrNF-YA2

PIYVNAKQYHGIIRRRQSRAKAVLKNKLTRVRKPYMHESRHLHAMRRPRGCGGRF

>JrNF-YA3

PIYVNPKQYHGILRRRQSRARLEAKTKVVKTRKPYLHESRHRHALNRVRGSGGRF

>JrNF-YA4

PIYVNPKQYHGILRRRQSRARLEAKTKVVKTRKPYLHESRHRHALNRVRGSGGRF

>JrNF-YA5

PIYVNAKQYHAILRRRQYRARLEAQNKVIKDRKPYLHESRHLHALKRARGSGGRF

>JrNF-YA6

PVYVNAKQYQGILRRRQARAKAELEKKLIKVRKPYLHESRHQHAMRRARASGGRF

>JrNF-YA7

PIYVNAKQYHGIIRRRQSRAKAVLENKLTRVRKPYMHESRHLHAMRRPRGCGGRF

>JrNF-YA8

PVYVNAKQYHGILRRRQSRAKAELEKKVIKVRKPYLHESRHLHAMRRARGCGGRF

>JrNF-YA9

PIFVNPKQYHGILRRRQSRAKLEAQNKVVKTRKPYLHESRHRHALNRVRGSGGRF

>JrNF-YA10

PIYVNSKQYHAILRRRQYRAKLEAQNKLIKDRKPYLHESRHLHALKRARGSGGRF

>JrNF-YA11

PVYVNAKQYQGILRRRQARAKAELEKKLIKVRKPYLHESRHQHAMRRARASGGRF

>JrNF-YA12

PVYVNAKQYHGILRRRQSRAKAELEKKVIKVRKPYLHESRHLHAMRRARGCGGRF

>JrNF-YA13

PVYVNAKQYQGILRRRQARAKAELEKKLIKVRKPYLHESRHQHAMRRARGSGGRF

>JrNF-YA14

PIYVNAKQYHGIIRRRQSRAKAVLKNKLTRVRKPYMHESRHLHAMRRPRGCGGRF

>JrNF-YA15

PIYVNPKQYHGILRRRQSRARLEAKTKVVKTRKPYLHESRHRHALNRVRGSGGRF

>JrNF-YA16

PIYVNSKQYHAILRRRQYRAKLEAQNKLIKDRKPYLHESRHLHALKRARGSGGRF

>JrNF-YA17

PVYVNAKQYHGILRRRQSRAKAELEKKVIKVRKPYLHESRHLHAMRRARGCGGRF

>JrNF-YB1

KEQDQLQLPIANVGRIMKQILPPNAKISKEAKETVQECVSEFISFVTGEASDKCHREKRKTVNGDDICCAMATLGFDDYGEPLRRYLEKYRE

>JrNF-YB2

REQD- RFLPIANISRIMKKALPANGKIAKDAKETVQECVSEFISFITSEASDKCQREKRKTINGDDLLWAMATLGFEDYIDPLKIYLSRYRE

>JrNF-YB3

REQD-RFLPIANISRIMKKALPANGKIAKDAKETVQECVSEFISFITSEASDKCQREKRKTINGDDLLWAMATLGFEDYIDPLKIYLSRYRE

>JrNF-YB4

REQD-RFLPIANVSRIMKKALPANAKISKDAKETVQECVSEFISFITGEASDKCQREKRKTINGDDLLWAMTTLGFEDYVEPLKVYLQRFRE

>JrNF-YB5

REQD-RFLPIANISRIMKKALPANGKIAKDAKETVQECVSEFISFITSEASDKCQREKRKTINGDDLLWAMATLGFEDYIDPLKIYLSRYRE

>JrNF-YB6

REQD-RFLPIANVSRIMKKALPANAKISKDAKETVQECVSEFISFITGEASDKCQREKRKTINGDDLLWAMTTLGFEEYVEPLKIYLQKYRE

>JrNF-YB7

REQD-RYLPIANISRIMKKALPANGKIAKDAKDTMQECVSEFISFITSEASDKCQKEKRKTINGDDLLWAMATLGFEDYIEPLKVYLARYRE

>JrNF-YB8

REQD-RLLPIANVSRIMKKALPANGKISKEAKETVQECVSEFISFITGEASDKCQREKRKTINGDDLLWAMTTLGFEEYVEPLKIYLQKFRE

>JrNF-YB9

KEQD-RFLPIANVGRIMKKVIPANGKISKDAKETVQECVSEFISFVTGEASDKCQREKRKTINGDDIIWAITTLGFEDYVSPLKTYLQKYRE

>JrNF-YC1

QQQLQMFWSYQRQEIE--QVNDFKNHQLPLARIKKIMKADEDVRMISAEAPILFAKACELFILELTIRSWLHAEENKRRTLQKNDIAAAITRTDIFDFLVDIVPRDEIK

>JrNF-YC2

QQQLQNFWANQYQEIE--KVTDFKNHSLPLARIKKIMKADEDVRMISAEAPVIFARACEMFILELTLRSWNHTEENKRRTLQKNDIAAAITRTDIFDFLVDIVPREDLK

>JrNF-YC3

QQQLQNFWATQYQEIE--QASDFKNHSLPLARIKKIMKADEDVRMISAEAPVIFARACEMFILELTLRSWNHTEENKRRTLQKNDIAAAITRTDIFDFLVDIVPREDLK

>JrNF-YC4

QQQLQSFWANQYQEIE--KATDFKNHSLPLARIKKIMKADEDVRMISAEAPVIFARACEMFILELTLRSWNHTEENKRRTLQKNDIAAAITRTDIFDFLVDIVPREDLK

>JrNF-YC5

QQQLQNFWATQYQEIE--QASDFKNHSLPLARIKKIMKADEDVRMISAEAPVIFARACEMFILELTLRSWNHTEENKRRTLQKNDIAAAITRTDIFDFLVDIVPREDLK

>JrNF-YC6

QQQLQMFWANQMQEIE--QTTDFKNHSLPLARIKKIMKADEDVRMISAEAPVIFAKACEMFILELTLRSWIHTEENKRRTLQKNDIAAAISRTDVFDFLVDIIPRDELK

>JrNF-YC7

QQQLQSFWANQYQEIE--KVTDFKNHSLPLARIKKIMKADEDVRMISAEAPVIFARACEMFILELTLRSWNHTEENKRRTLQKNDIAAAITRTDIFDFLVDIVPREDLK
