# Supplementary material for: Identification and characterization of NF-Y gene family in walnut (Juglans regia L.)
Source: BMC Plant Biol. 2018 Oct 23;18:255. doi: 10.1186/s12870-018-1459-2 (PMC6199752; doi:10.1186/s12870-018-1459-2)
Supplement: Supplementary file 7 — Primers involved in this article. (DOC 32.0 kb) [file 12870_2018_1459_MOESM7_ESM.doc]

**Additional file 7:** Primers involved in this article.

Primers involved in this article.

JrFT1:

Forward primer (5’-3’)： GCATTGGTTGGTGACTGATATTC

Reverse primer (5’-3’)： CCATAAGCATACGCACACTTCT

JrFT2:

Forward primer (5’-3’)： GGTGGAAGAAGAAGATGACTGAT

Reverse primer (5’-3’)： GATAGAACGAGATATGTGGTTGAGA

JrCO1:

Forward primer (5’-3’)： GCGGTGGTGGCAATAACTG

Reverse primer (5’-3’)： CTCATCTTCATCCTCTTCGTCAAT

JrCO2:

Forward primer (5’-3’)： AGTGCCTTGTCTGTGAGTGT

Reverse primer (5’-3’)： GCTTCCTGGTCTTGGTTCTTG

JrCO3:

Forward primer (5’-3’)： ACGAGAACAGTAGAGAAGTGCTA

Reverse primer (5’-3’)： TTCATCAGGAGAGTGGTGGATAT

JrNF-YA11:

Forward primer (5’-3’)： CTACCGTTACCATTGTTGCTATTG

Reverse primer (5’-3’)： CCTGATTCGTCCGCCACTA

JrNF-YA12:

Forward primer (5’-3’)： CAACAACCAACATACTCCAATGC

Reverse primer (5’-3’)： AGTCGTTCGTGCTGCTTCT
